# Hot-Carrier Injection and Millisecond Charge Separation from a Robust Heteroleptic Iron(II) Chromophore Immobilized on TiO_2_

**DOI:** 10.1021/jacs.5c22325

**Published:** 2026-05-07

**Authors:** Thomas Whittemore, Marvin Schmalle, Evgenia Ryndin, Mark Spitler, Elias H. P. Brohmer, Sven Rau, Linda Zedler, Evgeny O. Danilov, Felix N. Castellano, Stephan Kupfer, Gerald Meyer, Dieter Sorsche

**Affiliations:** † Department of Chemistry, 2331 University of North Carolina at Chapel Hill, Chapel Hill, North Carolina 27599, United States; ‡ Institute of Physical Chemistry, 9378 Friedrich Schiller University Jena, Lessingstraße 4, Jena 07743, Germany; § Leibniz Institute of Photonic Technology, 40096 Research Department Spectroscopy and Imaging, Albert-Einstein-Str. 9, Jena 07745, Germany; ∥ 9189 Institute for Inorganic Chemistry 1 Albert Einstein Allee 11 Ulm University, Ulm 89081, Germany; ⊥ Department of Chemistry, 6798 North Carolina State University, Raleigh, North Carolina 27695-8204, United States

## Abstract

The synthesis, spectroscopic characterization, computational analysis, and photoelectrochemical behavior of a new iron-based chromophore, [(Cpy)_2_Fe­(deeb)]­(PF_6_)_2_ **(Fe­(Cpy)**_**2**_ **(deeb))**, where Cpy is 1-methyl-3-(2-pyridyl)­imidazole and deeb is 4,4’-(CO_2_CH_2_CH_3_)_2_-2,2’-bipyridine, is reported. Electrochemically reversible waves assigned to a metal-centered E^o^(Fe^III/II^) = +0.48 and a ligand-centered E^o^(Fe^2+/+^) = −1.47 V vs Fc^+/0^ reduction were evident in cyclic voltammetry measurements. The combination of a strong σ-donor and a π-acceptor lowered the energy of the metal-to-ligand charge-transfer (MLCT) excited state relative to the metal-centered state. Two MLCT transitions appear in the visible region at 424 and 580 nm. TDDFT calculations revealed that the lower-energy band was well formulated as Fe­(II)→deeb, and the higher-energy transition was charge transfer to both the deeb and Cpy ligands. Resonance Raman spectroscopy supports these findings showing enhanced deeb vibrational modes with 532 nm excitation, both deeb and Cpy modes with 473 nm excitation, and exclusively Cpy with 405 nm excitation. Ultrafast spectroscopy reveals a short-lived (∼2 ps) MLCT excited state and a longer-lived (∼20 ps) metal-centered state. Efficient methods to deprotect the ester groups and anchor the complex to mesoporous TiO_2_ (anatase) thin films in high surface coverages, **Fe­(Cpy)**_**2**_ **(dcb)|TiO**_**2**_ σ = 3 × 10^–8^ mol/cm^2^, were established. Pulsed light excitation of **Fe­(Cpy)**_**2**_ **(dcb)|TiO**_**2**_ resulted in rapid excited state injection (*k*_*inj*_ > 10^8^ s^–1^) and formation of a charge-separated state, **Fe** ^**III**^ **(Cpy)**_**2**_ **(dcb)|TiO**_**2**_ **(e),** which persists on the millisecond time scale before returning cleanly to the ground state with second-order kinetics. Injection yields measured 50 ns after light excitation were found to double from Φ = 0.15 with green (532 nm) light to 0.30 with blue (457 nm) light excitation. Incident photon-to-current efficiency (% IPCE) measurements as a function of excitation wavelength in a 0.5 M LiI/I_2_/CH_3_CN electrolyte provide clear evidence for band-selective “hot carrier” injection from the remote Cpy-localized excited state. Collectively, the spectroscopic and photoelectrochemical data indicate that a semiconductor can intercept hot electrons from iron chromophores even when the excited-state dipole is oriented away from the surface-anchoring ligand.

## Graphical abstract

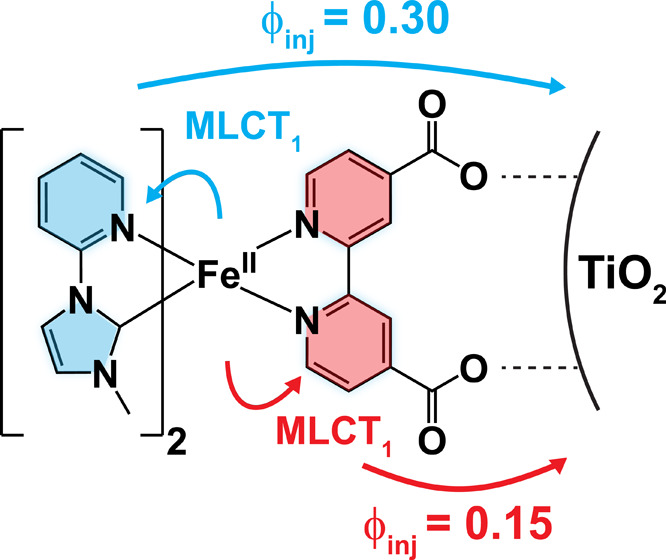

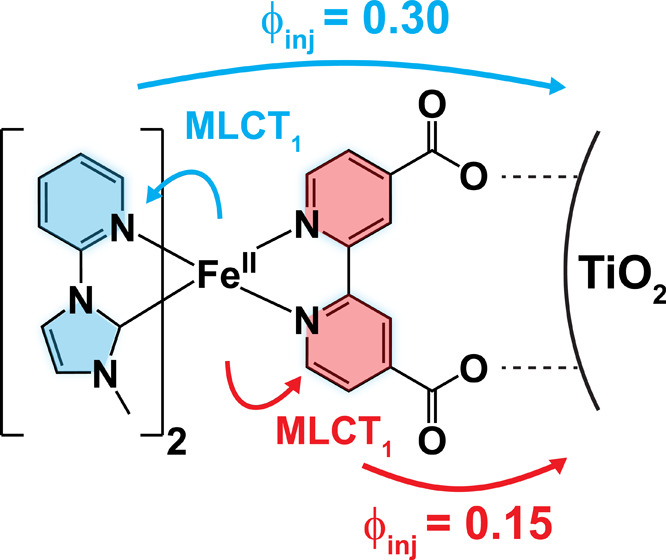

## Introduction

Developing a sustainable energy economy based on renewable resources remains one of today’s most pressing global challenges. Solar energy, the primary low-entropy energy source on Earth, lies at the heart of this endeavor. Efficient conversion of solar energy into electricity^1–4^ or chemical products^5–8^ represents both a fundamental challenge and a technological hurdle. At the molecular level, absorption of a solar photon by a molecule creates an electronic excited state that is assumed to retain the nuclear geometry of the ground state, in accordance with the Franck–Condon principle.^9^ Relaxation to a thermally equilibrated excited state involves nuclear motion and a loss of free energy. Absorption of photons whose energy exceeds the energetic gap between the ground state and the thermally equilibrated excited state is lost thermally as heat. These considerations led Shockley and Queisser to establish a theoretical maximum efficiency of ∼33% for solar energy conversion when charge extraction occurs from a thermally equilibrated excited state.^10^ Later, Ross and Nozik proposed a “hot carrier flat-panel solar cell” that promised efficiencies of up to 66% by harvesting energy from nonequilibrated excited states before relaxation occurs.^11^ While the solar cell they envisioned remains unrealized, there is growing evidence for hot-carrier electron transfer in molecular,^12–16^ heterogeneous,^17–19^ and interfacial systems.^20–25^ In photophysical studies, photon emission from higher excited states—termed “anti-Kasha” behavior—is also directly relevant to these hot-carrier processes.^26–28^ Taken together, the literature data suggest that exceeding the Shockley–Queisser limit may be attainable.

Transition metal (TM) complexes—particularly those with a (dπ)^6^ configuration and metal-to-ligand charge transfer (MLCT) excited states—offer a promising platform to study and optimize hot-carrier transfer. Complexes of the form [M­(LL)_3_]^2+^, where LL is a bidentate chelating diimine ligand, typically display strong charge-transfer absorption bands in the visible region. Through synthetic modification, these absorption features can be tuned to desired wavelengths.^4^ Heteroleptic complexes containing two or three distinct LL ligands can exhibit multiple MLCT bands, enabling wavelength-selective excitation to specific ligands with distinct spatial and electronic properties (see Scheme 1).^29^ It is well-known that in such systems, the excited state rapidly localizes on the ligand that is most easily reduced. Achieving exclusive charge transfer to a single ligand is synthetically demanding, and overlapping MLCT bands can hinder selective excitation. For example, ruthenium complexes bearing three different ligands have been synthesized and characterized, and the charge transfer band to each ligand overlap in wavelength, preventing quantitative ligand-specific photoreduction.^30^ More commonly, ruthenium complexes are designed with two identical ligands and one distinct ligand to preserve some selectivity.^31^

**Scheme 1:**
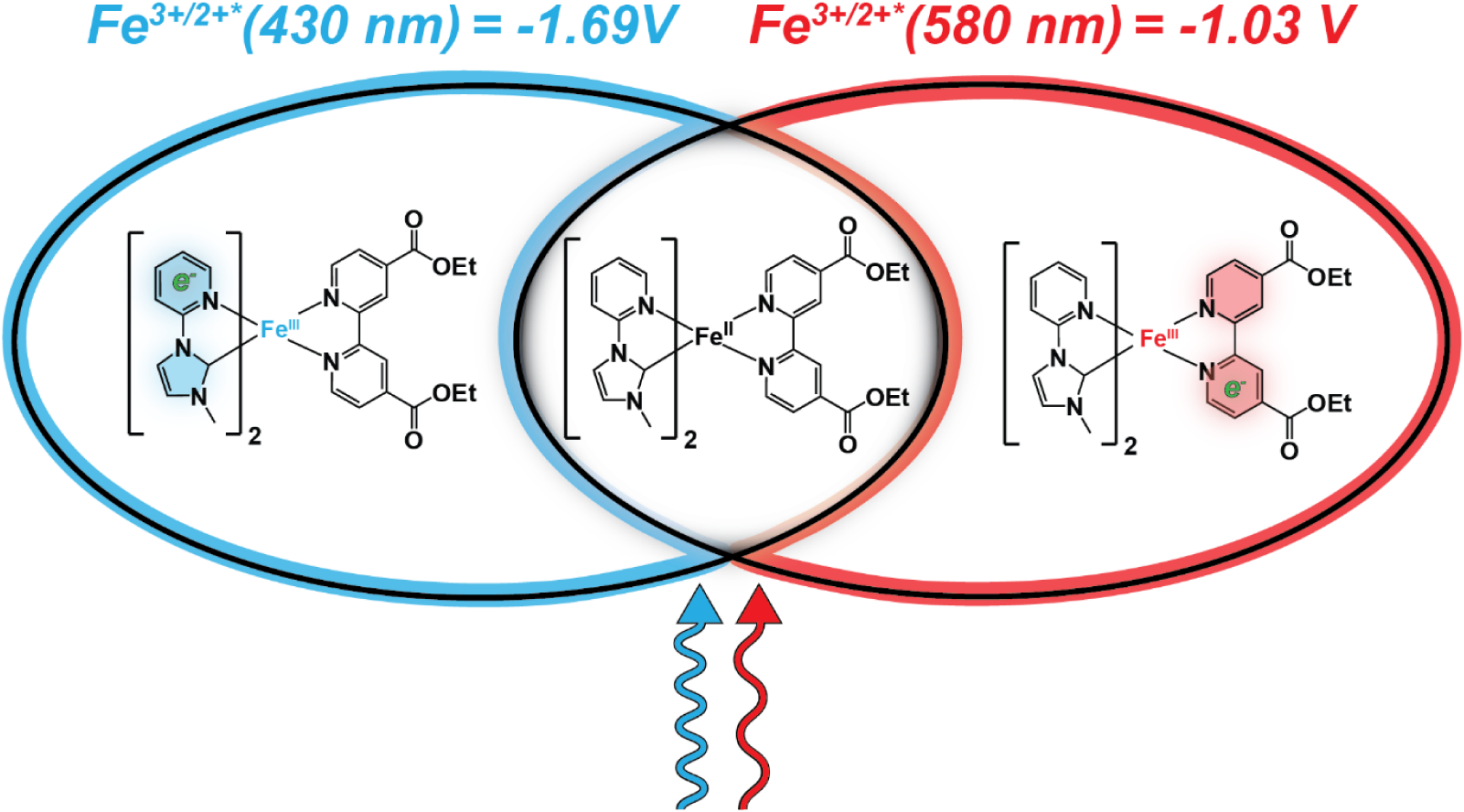
Metal-to-Ligand Charge Transfer (MLCT) Accessible with Blue and Red Light that Generate Excited States with Reduction Potentials that Vary over 600 mV

The upper electronic excited states of [Ru­(LL)_3_]^2+^ TM complexes are typically depopulated on the picosecond time scale.^32^ Consequently, their practical utilization in photoredox chemistry requires subpicosecond electron transfer. Recently, computational investigations have shown that electron transfer from nonrelaxed excited states of Fe­(II) complexes can occur.^33,34^ Although only a limited number of iron complexes have been explored experimentally for light-to-electrical energy conversion, all reported examples with two absorbance bands in the visible region exhibit distinct “band-selective” efficiencies.^35–37^ Plots of the incident photon-to-current efficiency (% IPCE) versus the excitation wavelength, i.e., a photocurrent action spectrum, demonstrate light excitation into one absorption band yields a more efficient photocurrent than does excitation into another. In contrast, among hundreds of Ru complexes that have been characterized such band-selective behavior is rare.^24^ The two most widely studied Fe­(II) complexes employed in solar cells are shown in Scheme 2. Ferrere and Gregg first reported band-selective behavior for **Fe-1** and McCusker and Jakubikova subsequently provided new insights into the profound impact of electrolyte ions have on the electronic structure.^36,38,39^ Later, Wärnmark, Gros, and coworkers showed that **Fe-2** exhibits two visible charge-transfer bands with very similar extinction coefficients, nevertheless, in the photocurrent action spectrum of the higher-energy band generated about 30% more photocurrent than excitation of the lower-energy band.^37,40^

**Scheme 2:**
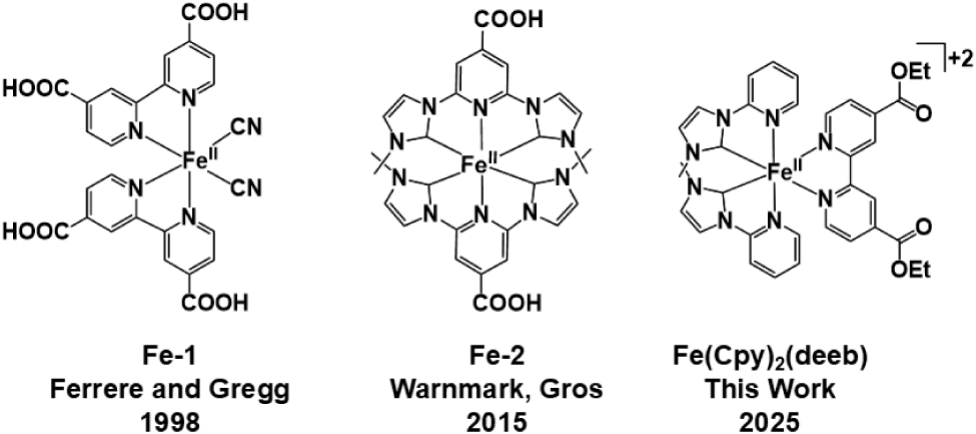
Previously Reported Iron Complexes **Fe-1** and **Fe-2** and the **Fe­(Cpy)**_**2**_ **(deeb)** Complex Reported in This Work that Display Band-Selective Hot Injection

The pronounced tendency for iron to undergo “band-selective” electron transfer—unlike the relatively rare cases observed for ruthenium^24^—may be due to several factors. McCusker has highlighted the importance of the iron 3d orbitals in the metal–ligand orbital overlap, which lack radial node(s) found in the 4d and 5d orbitals of Ru and Os.^41^ Additionally, electroabsorption (Stark) spectroscopy has revealed a greater extent of excited-state charge transfer in iron complexes relative to Ru and Os, likely due to weaker metal–ligand coupling in the ground state.^42^ The greater extent of charge transfer with a smaller spin–orbit coupling constant may also lead to less mixing of spin states, as appears to be the case in this study.^43^ The choice of iron in this study is further motivated by widespread interest in earth-abundant metals for photoredox applications.^44–46^ Examples of earth-abundant transition-metal photosensitizers include complexes of Ti,^47,48^ Zr,^49,50^ Cr,^51,52^ and Cu,^53,54^ but special attention has been devoted to Fe^55–60^ due to the shared periodicity to Ru, low cost, and high natural abundance. The recent development of a photoluminescent iron complex marks a promising advance in this area.^61^ Although *N*-heterocyclic carbene (NHC) ligands have been successfully employed to prolong the excited-state lifetimes of iron complexes,^62^ the key differences in interfacial electron transfer for complexes of iron relative to complexes of ruthenium are not well understood.

Herein, we report a heteroleptic iron­(II) complex bearing two unique chelating ligands, [(Cpy)_2_Fe­(deeb)]­(PF_6_)_2_, abbreviated (**Fe­(Cpy)**_**2**_ **(deeb)**) and shown in Scheme 1. This complex exhibits exceptional stability in both its one-electron reduced and one-electron oxidized states and incorporates strongly σ-donating N-heterocyclic carbene (NHC) ligands, which create a ground- and excited-state electronic structure comparable to polypyridyl ruthenium­(II) complexes. Owing to its heteroleptic design, **Fe­(Cpy)**_**2**_ **(deeb)** displays broad, panchromatic absorption characterized by two distinct MLCT bands: a lower-energy transition assigned to Fe­(II) → deeb and a higher-energy band assigned predominantly to Fe­(II) → Cpy, Scheme 1. The spectral purity of these transitions was probed by computational analysis and resonance Raman spectroscopy as is discussed further herein. Importantly, these two charge transfer transitions differ in excited-state reduction potential by more than 600 meV. Consequently, excitation of a solution of **Fe­(Cpy)**_**2**_ **(deeb)** with both blue and red light generates a population of charge transfer excited states with unique structural and energetic properties. Specifically, blue light promotes an electron to the Cpy ligand (Fe^3+/2+*^ = −1.69 V), and red light promotes an electron to the deeb ligand (Fe^3+/2+*^ = −1.03 V). Iron-based chromophores have been demonstrated to perform electron transfer catalysis,^63,64^ and the ability to provide reducing equivalents at two distinct potentials may enhance catalysis with preferential transfer to spatially arranged acceptors. To the best of our knowledge, this report provides the first experimental evidence of “hot” interfacial electron transfer from a chomophoric ligand that is not directly anchored to the semiconductor surface. The results highlight how energy alignment between the donor excited state and the semiconductor acceptor levels—rather than excited-state lifetime or the spatial orientation of the excited-state dipole—predominantly govern the efficiency of hot carrier electron transfer. The principles established from this report offer new design strategies and implementation of iron or first-row transition metal chromophores for use for interfacial electron transfer applications.

## Results

### Synthesis and Structural Characterization

A major drawback for the development of advanced iron chromophores is the lack of strategies to synthesize heteroleptic iron­(II) complexes. Here, we expand the scope of a recently reported approach by introducing the ester-protected anchoring ligand 4,4’-(CO_2_CH_2_CH_3_)_2_-2,2’-bipyridine, deeb.^65^ Reaction of the precursor and deeb in acetone leads to the formation of the new complex [(Cpy)_2_Fe­(deeb)]­(PF_6_)_2_, (**Fe­(Cpy)**_**2**_ **(deeb)**), in 58% yield after recrystallization from acetontrile/diethyl ether as a dark-green solid. This synthetic approach yields different isomers based on the relative orientation of the Cpy ligands. As before, we find a *C*_2_ symmetric isomer with the two pyridine moieties of the Cpy ligand *trans* relative to each other (N,N-axial) to be the dominant species. We also identify a second isomer where one Cpy ligand is flipped, placing one carbene *trans* to the pyridine of the respective other Cpy ligand (C,N-axial). Unlike in our previous report, we now find evidence for a third isomer with both carbene ligands *trans* to each other (C,C-axial, *C*_2_ symmetry), based on an additional set of signals in the ^1^H NMR and ^13^C NMR data (see figure S1 for proposed structures and figures S2–S7 for corresponding NMR spectra). This third isomer has not been accessible with stronger donating NN-chelate bibenzimidazole ligands.^65^ High-resolution mass spectrometry data additionally confirmed the composition of the complexes and can be found in the SI (Figure S8). The observation of these isomers and what determines their formation and relative distribution is a challenging aspect of the Cpy system, which will be discussed elsewhere. Relevant insights regarding the effect of different substitution patterns in Cpy-type ligands were previously reported by Gros and coworkers.^66–68^ All experiments reported here were carried out with an isomer ratio of about 10:4:1 after recrystallization (cp. Figure S5).

Single crystals suitable for X-ray diffraction analysis were obtained through crystallization from an acetonitrile solution layered with diethyl ether. The crystalline samples were generally of poor quality. Sufficiently crystalline samples reveal the predominant *C*_2_-symmetric N,N-axial isomer of **Fe­(Cpy)**_**2**_ **(deeb)** crystallized in the orthorhombic space group *Aba*2 (Figure 1). However, note that other isomers were likely cocrystallized in an irregular, disordered fashion as is evident from the unusually large and anisotropic thermal ellipsoids found for the Cpy ligands, as well as residual electron densities indicating the respective alternative orientations of Cpy ligands. A ^1^H NMR spectroscopic analysis of the redissolved crystalline sample also indicated that all isomers were still contained in the bulk crystalline sample.

**Figure 1:**
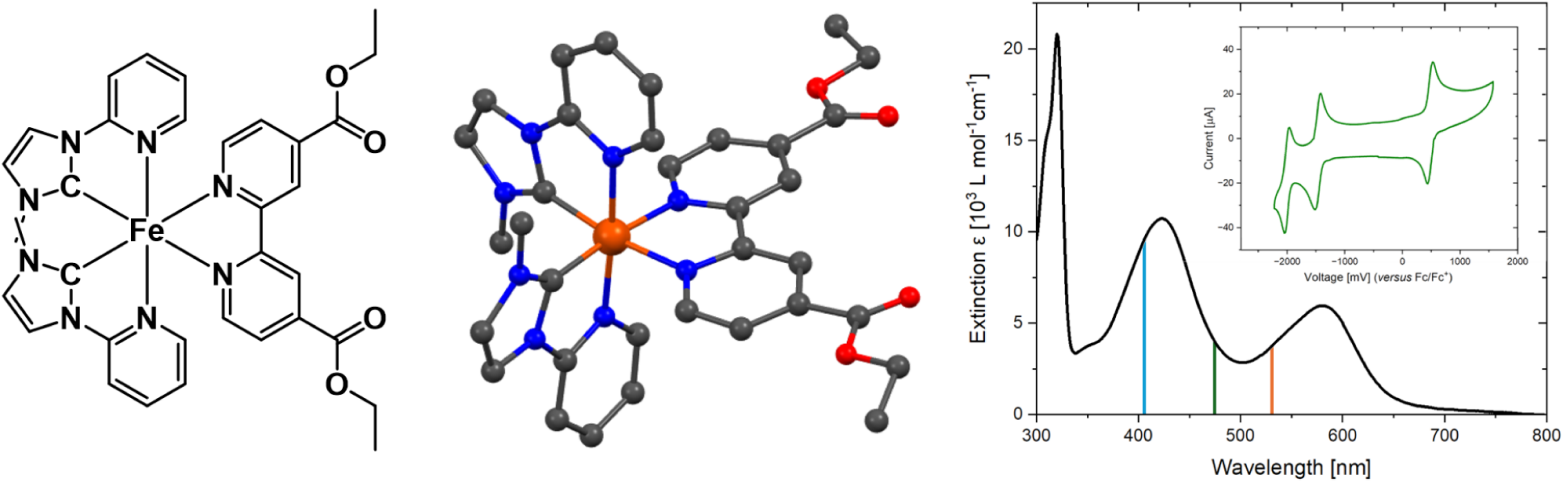
Schematic depiction (left) and solid-state structure (center) of **Fe­(Cpy)_2_(deeb)** showing the C_2_-symmetric cation crystallized as its (PF_6_)_2_ salt; hydrogen atoms, counterions, and cocrystallized solvent molecules are omitted for clarity; right: steady-state UV–vis spectrum of **Fe­(Cpy)_2_(deeb)** in acetonitrile, inlay showing the cyclic voltammogram.

The solid-state structure confirms the expected composition of the heteroleptic complex **Fe­(Cpy)**_**2**_ **(deeb)** containing two Cpy ligands and one deeb ligand. In the C_2_-symmetric isomer the two Cpy ligands are oriented such that the two pyridine moieties bind to iron *trans* relative to each other (N,N-axial). Consequently, the two nitrogen donors of the deeb ligand are oriented *trans* relative to the NHC ligands. The two iron–carbon bonds are 1.920(8) Å and 1.930(9) Å, and the respective Fe–N bonds are 1.998(7) Å and 1.977(7) Å long which is well within the range reported for iron complexes with bidentate NHC-pyridine ligands.^37,62,69,70^ The Fe–N bonds with the deeb ligand are 1.982(6) Å and 1.947(6) Å which is also within the usual range for bipyridine iron­(II) complexes. These bonds are, however, significantly shorter than the Fe–N bonds with 2,2’-bibenzimidazole reported in a previous study.^65^ The deeb ligand shows a marginal deviation from planarity which may be attributed to packing effects. The ethyl ester substituents are virtually coplanar with the 2,2’-bipyridine as characterized by torsion angles of 5.34° and 8.54° between the carboxylate functions and the respective pyridyl rings. A review of the crystallographic literature reveals that the ester (or carboxylic acid) groups are nearly coplanar in ten different complexes bearing deeb (or dcb) ligands.^71–75^ This configuration may result from attractive C–H—O dipole–dipole interactions between the 3- and 5-positions of the pyridines and the oxygen atoms in the ester group. The lack of a resonance enhancement of the ester (or carboxylic acid) groups in the resonance Raman spectra of the solvated complex suggests that these groups may adopt a more orthogonal geometry in fluid solution. Relevant crystal structure data can be found in the Supporting Information (Figures S10 and S11, Tables S 1 and S2).

### Electrochemistry

Cyclic voltammetry reveals three distinct redox processes in the potential range between −2.5 V and + 1.5 V (vs Fc^+/0^) for **Fe­(Cpy)**_**2**_ **(deeb)** (Figure 1, right, see also Figure S11). Note that the cyclic voltammogram represents the isomer mixture as described above. A fully reversible oxidation at +0.48 V (vs Fc^+/0^) was assigned to the Fe^III/II^ redox process, which was shifted to more positive potential by about 200 mV compared to the previously reported 2,2’-bibenzimidazole complex (Table 1),^65^ suggesting that the iron­(II) center in **Fe­(Cpy)**_**2**_ **(deeb)** is more electron-deficient. In this context, the observed shift reflects exchange of the electron-rich, strongly electron-donating 2,2’-bibenzimidazole ligand with the electron-deficient deeb ligand. The two well-resolved, and clearly separated reversible reduction processes at −1.47 V (vs Fc^+/0^) and at −2.00 V (vs Fc^+/0^) are attributed to the reductions of the deeb and one Cpy ligand, respectively.

**Table 1:** Reduction Potentials of Fe­(Cpy)_2_(deeb) and Reference Complexes^a^

Complex^b^	E^o^(M^2+/+^)	E^o^(M^III/II^)
**Fe(Cpy)**_**2**_ **(deeb)**	–1.47 V (rev)	+0.48 V (rev)
**Ru-1**	–1.43 V (rev)	+0.88 V (rev)
**Fe-1**	–1.47^c^ (rev)	+0.35^c^ (rev)
**Fe-2**	–1.71 V^d^ (irr)	+0.45^d^ (rev)

a All measurements in a 0.1 M TBAPF_6_ acetonitrile solution in acetonitrile versus the pseudo-reference Fc^+/0^. The reductions at positive applied potentials are metal (M^III/II^)-based and the reductions at negative potentials are ligand (M^2+/+^) based.

b The complexes shown in Scheme 2 and Ru-1 is [Ru­(dtb)_2_(deeb)]­(PF_6_)_2_ where dtb is 4,4”-(tert-butyl)­2-bpy and deeb is 4,4”-(CO_2_CH_2_CH_3_)_2_-bpy.

c from refs. 36,38 reported values referenced versus SCE, values calculated with Fc^+/0^ couple at +0.40 V versus SCE.^76^.

d from ref. 77.

The first reduction of **Fe­(Cpy)**_**2**_ **(deeb)** compares well with values reported for related ruthenium­(II) complexes bearing a deeb ligand.^78^ Our own data obtained for **Ru-1** compares well with **Fe­(Cpy)**_**2**_ **(deeb)** (Figures S12 and S13, Table S3) and indicate that the ligand reduction potential is insensitive to the identity of the divalent metal (Ru vs Fe). The second reduction process for **Fe­(Cpy)**_**2**_ **(deeb)** appearing at −2.00 V fits well with our previously reported data as well as the data reported by Monari, Cebrian, and Gros et al.^67^ on the tris-homoleptic complexes of the Cpy ligand. This strongly supports assignment of this redox event to the reduction of the Cpy ligand, i.e., Cpy ^± 0/‑1^. However, it is worth noting that the electrochemical reversibility reported here is unusual for this type of carbene/imine reduction.

### Steady-State Absorption

Acetonitrile solutions of **Fe­(Cpy)**_**2**_ **(deeb)** reveal two distinct absorption maxima in the visible, one in the blue at 424 nm (∼11 × 10^3^ L mol^–1^ cm^–1^), and one in the red at 580 nm (∼6 × 10^3^ L mol^–1^ cm^–1^), as well as a sharp intense absorption in the UV region at 320 nm (21 × 10^3^ L mol^–1^ cm^–1^) (Figure 1 right and Figure S14). Based on their energy and extinction coefficients, the two visible light absorption bands are attributed to metal-to-ligand charge transfer (MLCT) processes, while the UV absorption likely refers to an intraligand π-π* transition. Considering the well-resolved redox processes discussed above, it is reasonable to assign the two distinct visible light absorption bands to two distinct MLCT processes, namely transferring a negative charge formally from iron to either the deeb ligand upon absorption at long wavelengths, or to the Cpy ligand upon absorption at short wavelengths. These assignments were further evaluated and confirmed through resonance Raman spectroscopy and theoretical calculations, as detailed below.

### Theoretical Calculations

TDDFT simulations were performed to elucidate the underlying electronic structure of **Fe­(Cpy)**_**2**_ **(deeb)** (C_2_ isomer, N,N-axial). Representative data are shown below, and additional data can be found in the SI (Figures S15–S 21, Tables S 4–S13). The lower energy 580 nm absorption was attributed to two MLCT transitions to the lowest energy π_deeb_* of the deeb ligand, S_3_ and S_4_ at 596 and 539 nm in Figure 2A and B. The higher energy absorption band in the visible region at 424 nm was also of MLCT character, the red flank was related to two dipole-allowed transitions to the deeb ligand (into S_8_ and S_9_; 479 and 478 nm), and the blue flank was assigned to two dipole-allowed transitions to the Cpy ligands (into S_14_ and S_18_; 430 and 400 nm). Finally, TDDFT predicted an intraligand transition of the deeb ligand at 323 nm (into S_31_), which was assigned to the experimental absorption band at 320 nm. Note that three different hybrid functionals with medium to a low amount of exact exchange (B3LYP with 20%, TPSSh with 10%, and B3LYP10 with 10%) were utilized to investigate the ground and excited state properties of **Fe­(Cpy)**_**2**_ **(deeb)**. All methods gave fully consistent results, while merely the predicted excitation energies are coherently hypsochromically shifted with increasing Hartree–Fock exchange.

**Figure 2:**
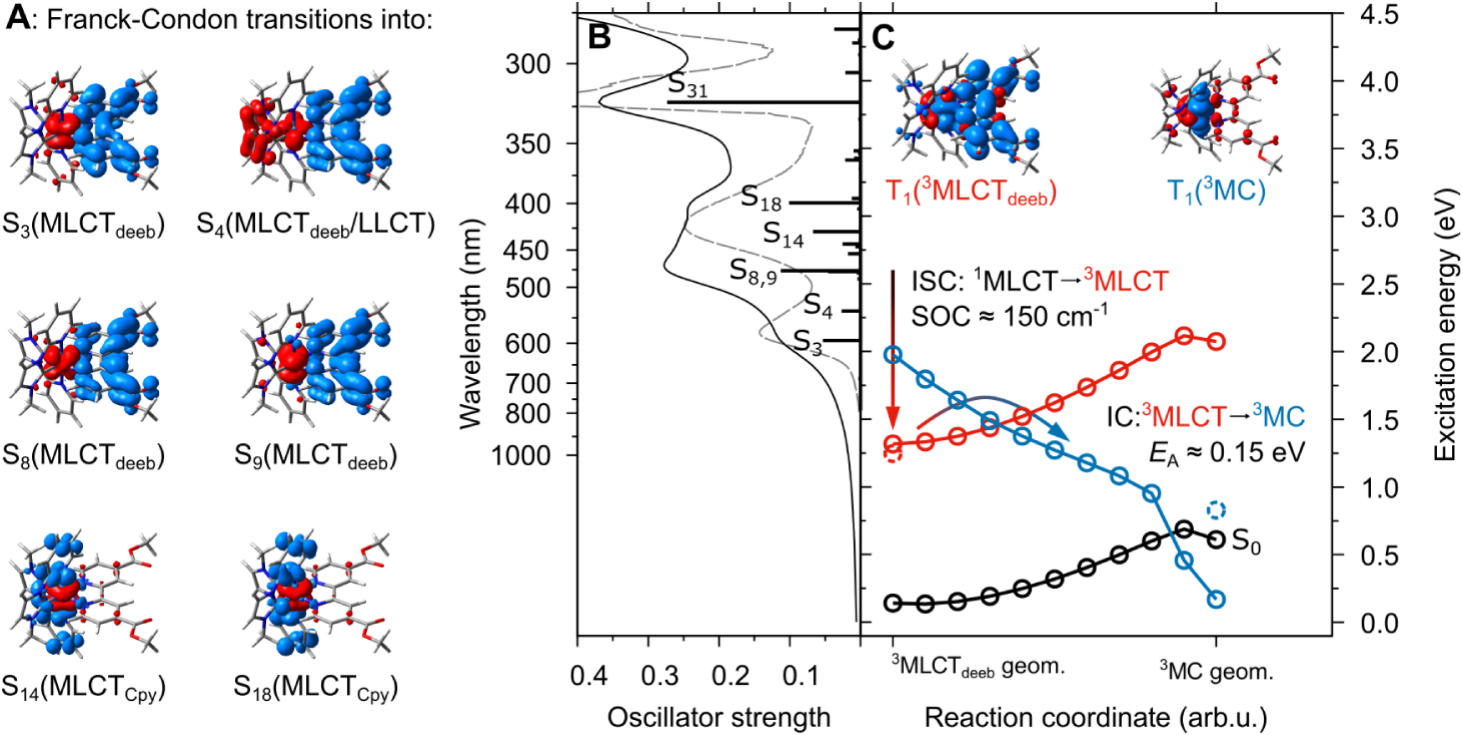
(**A**) Dipole-allowed transitions involved in the UV–vis absorption spectrum of **Fe­(Cpy)_2_(deeb)** computed by TDDFT. (**B**) Charge density difference plots (CDD; charge transfer occurs from red to blue) and the simulated (black, solid; TD-B3LYP10/def2-svp) and measured (dashed, gray) absorption spectrum in acetonitrile; key transitions are labeled. (C) Excited state relaxation processes through intersystem crossing (ISC) along the singlet-to-triplet MLCT gateway states and the associated spin–orbit couplings (SOCs) as well as internal conversion from the fully relaxed lowest energy ^3^MLCT_deeb_ state (red) to the equilibrated low-energy ^3^MC state (blue); spin densities are given as inset. The respective potential energy curves—connecting the two triplet equilibrium structures (^3^MLCT_deeb_ and ^3^MC)—are obtained along a linear interpolated internal coordinate with a 0.15 eV activation barrier.

### Vibrational Spectroscopy

Resonance Raman (rR) spectroscopy was applied to unambiguously assign the nature of the electronic transitions within the Franck–Condon regime in combination with the performed TDDFT simulations. Resonance Raman spectra provide insight into the structure of the Franck–Condon absorption point, thereby defining the initial geometry for electron transfer in the electronically excited state(s) upon excitation within the two visible charge transfer bands of **Fe­(Cpy)**_**2**_ **(deeb)** at 532, 473, and 405 nm, i.e., in resonance with the initial ^1^MLCT transition (Figure 3 A–C). At 532 nm, MLCT transitions are probed, which involve the deeb ligand, i.e., with the MLCT_deeb_ states S_3_ and S_4_ 9at 596 and 539 nm, see Figure 2).

**Figure 3:**
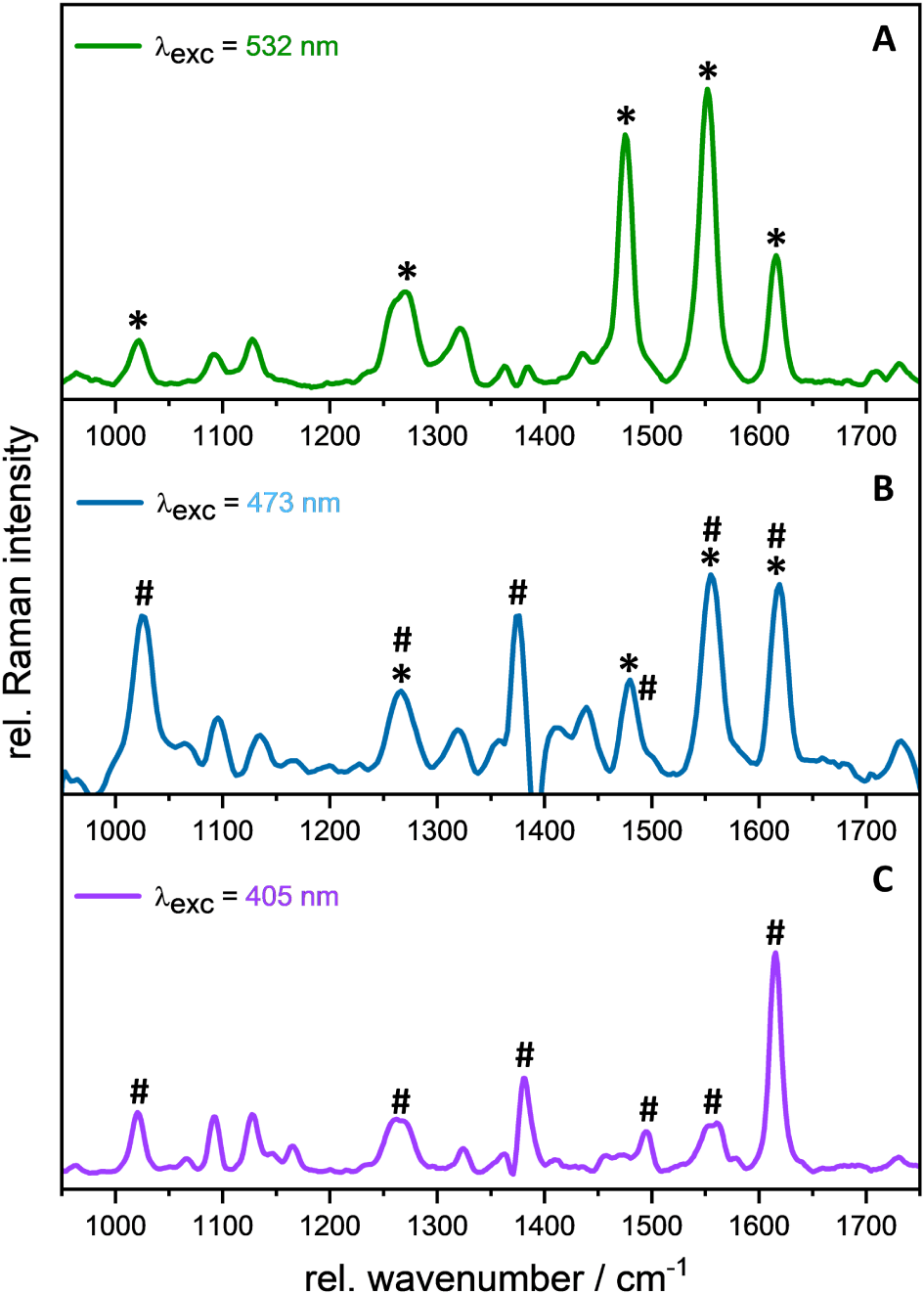
Resonance Raman spectroscopy of **Fe­(Cpy)_2_(deeb)** in acetonitrile at the excitation wavelengths 532 (A), 473 (B), and 405 nm (C). Raman bands assigned to the deeb ligand are marked with an asterisk (*), while those corresponding to the Cpy ligand are labeled with a hash symbol (#).

The rR spectrum recorded at this excitation wavelength reveals a set of modes at 1271, 1476, 1551, and 1615 cm^–1^ characteristic of the coordinated deeb ligand (Figure 3A, bands are marked with an asterisk).^79,80^ Increasing the excitation energy from 532 to 473 nm resulted in the resonance enhancement of Cpy-associated bands at 1026 and 1384 cm^–1^ due to the involvement of both ligand spheres in the initial photoexcitation process (Figure 3B). This finding is in full agreement with the quantum chemical simulations, which predicted a set of two MCLT_deeb_ transitions at 479 and 478 nm (S_8_ and S_9_), while two dipole-allowed MLCT_NHC_ transitions were localized at 430 and 400 nm (S_14_ and S_18_), respectively. The rR spectrum of **Fe­(Cpy)**_**2**_ **(deeb)**, excited at 405 nm mainly resembled the off-resonance Raman spectrum of the solid homoleptic [Fe­(Cpy)_2_(MeCN)_2_]^2+^ complex^65^ which served as a reference compound for the respective characteristic ^1^MLCT signals (Figure 3C, bands are marked with a hash symbol, Figure S22) with modes at 1615, 1562, 1498, 1384, 1095, and 1026 cm^–1^. Thus, the highest energy excitation of **Fe­(Cpy)**_**2**_ **(deeb)** leads to the population of the π* orbital of the Cpy ligand.

### Excited-State Dynamics

Transient absorption difference spectra measured after pulsed 400 and 620 nm light excitation of **Fe­(Cpy)**_**2**_ **(deeb)** dissolved in neat acetonitrile are presented in Figure 4. These two wavelengths were selected as they excite the blue flank of the higher-energy Fe­(II) → Cpy charge transfer band and the low-energy flank of the Fe­(II) → deeb absorption band with minimal overlap. Two pronounced bleaches, centered at about 424 and 580 nm were present with both excitation wavelengths. Kinetic data recorded at the bleach maxima were well described by a biexponential kinetic model, revealing to one significant figure a 2 ps and a 20 ps component (Figure S23). A weak positive absorption between 650 and 750 nm was present at the earliest observation times that decayed to the initial amplitude with first-order kinetics (τ = 2 ps, Figure S23).

**Figure 4:**
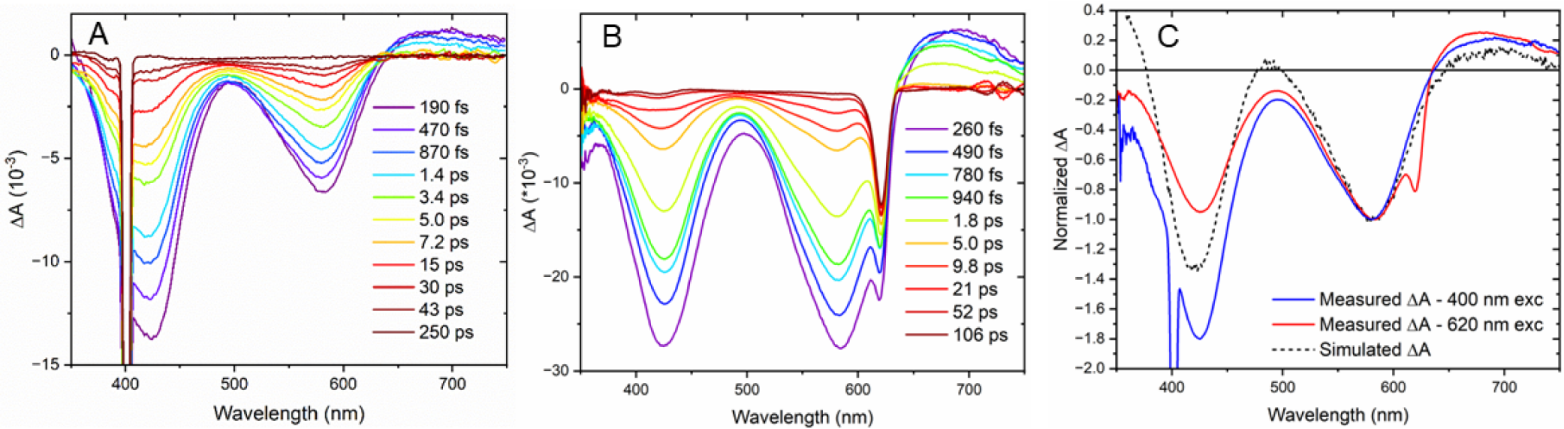
Absorption difference spectra measured at the indicated delay times after pulsed a) 400 nm and b) 620 nm light excitation of **Fe­(Cpy)_2_(deeb)** in neat CH_3_CN. c) The absorption difference spectrum measured 490 fs after 400 nm (blue) and 620 nm (red) pulsed light excitation of **Fe­(Cpy)_2_(deeb)**; overlaid in black dashes is a simulated difference spectrum of the MLCT excited state: △A­(modeled) = (Abs­(**[Fe­(Cpy)_2_(deeb)]^3+^**) + Abs­(**[Fe­(Cpy)_2_(deeb)]^+^**)) – Abs­(**[Fe­(Cpy)_2_(deeb)]^2+^**).

In order to evaluate the nature of the electronic processes associated with excited state relaxation pathways, the MLCT absorption was modeled from the spectra of the oxidized and reduced complex generated in a spectroelectrochemical cell that were summed and then subtracted from the ground state; i.e., ΔAbs­(MLCT) = (Abs­(**[Fe­(Cpy)**_**2**_ **(deeb)]** ^**3+**^) + Abs­(**[Fe­(Cpy)**_**2**_ **(deeb)]** ^**+**^)) – Abs­(**[Fe­(Cpy)**_**2**_ **(deeb)]** ^**2+**^). This simulated spectrum accurately modeled the long-wavelength positive absorption band; hence, the 2 ps component was assigned to an MLCT excited state. The simulation did not reproduce the expected excited-state absorption centered about ∼380 nm as is expected for an MLCT of a polypyridyl ruthenium complex. Accordingly, we attribute the absence of the ESA to the presence of a MC state,^81^ which is expected to have a large bleach in this region as is discussed below.

The existing literature of homoleptic Fe­(II) complexes that display spin-crossover behavior provides some insight into the difference spectra expected when an iron complex converts between spin states.^82–84^ In general, the spectra of low-spin (singlet) and high-spin (triplet or quintet) states are similar; however, the population of antibonding orbitals in high-spin states results in longer Fe-ligand bonds and hence an extinction coefficient that is at least a factor of 10 smaller.^82^ Therefore, to a first approximation, the population of MC states is expected to give rise to a ground state bleach without any positive absorption bands. On this basis, the 20 ps component was assigned to the MC state.

A surprising result was that the relative bleach amplitudes were excitation wavelength dependent (Figure 4). With 400 nm excitation, the high-energy band was roughly twice as intense as the low-energy band, whereas with 620 nm excitation, the two bleaches had roughly the same amplitudes. The spectra measured with 400 nm excitation after the MLCT excited state had decayed, mirrored the ∼2:1 amplitudes of the ground state and were hence consistent with the stated expectations for an MC state. This cannot, however, explain the spectra measured with 620 nm light.

A complete explanation for the dramatically different spectra measured after pulsed 400 and 620 nm excitation will require additional study that goes beyond the scope of this work focused on band-selective excited state electron transfer. We do note however that our simplified “expected“ absorbance spectrum of the MC state was based on previous studies of *homoleptic* Fe­(II) complexes.^82–84^ Since heteroleptic Fe­(II) complexes with two separate charge transfer bands in the visible region are rare, spectral data relevant to this complex was not found in the literature. The initial assumption that the decreased extinction coefficient in the MC state would be proportionally the same for the Fe­(II) → Cpy (420 nm) and the Fe­(II) → deeb (580 nm) must be in error. To be fully consistent with the experimentally measured spectra, the extinction coefficient for the Fe­(II) → Cpy must be approximately twice that of Fe­(II) → deeb in the MC state generated with 620 nm light. However, we cannot rule out the presence of additional excited states.

At a minimum, the variable excitation wavelength data indicate that two different low-energy states are created with blue and red light, which are presumably associated with the excited state relaxation pathways from the ^3^MLCT_Cpy_ (at 400 nm excitation) vs the ^3^MLCT_deeb_ (at 620 nm excitation) states. Biphasic kinetics have been previously reported for a range of Fe-NHC complexes, and assignment of the constituent excited states needs to be done with care.^85–87^ Intersystem crossing (ISC) is reported to occur in less than 100 fs for polypyridyl complexes of iron^86,88^ suggesting a full conversion of all ^1^MLCT states to their triplet counterparts within the time resolution of our instrument. It is also known that polypyridyl complexes of iron can decay to an ^5^MC state that can live for hundreds of picoseconds to nanoseconds;^87^ such a long-lived state was not observed here, suggesting population of only the comparatively short-lived ^3^MC state, consistent with a prior report.^65^ The ultrafast dynamics and spectral data can be explained by a model wherein the two MLCT excited states formed by selective excitation of the two visible absorption bands are independent of one another and are connected only through the ground state. Interestingly, the most comprehensive ultrafast study of Fe­(II) N-heterocyclic carbene complexes by Persson, Yartsev, and coworkers^85^ reported similar behavior, i.e., a short-lived MLCT state and a ∼20 ps MC state. Their mechanistic analysis indicated two different underlying excited state relaxation mechanisms: a consecutive model in which one state feeds another, and a parallel model in which the states are populated with light and decay independently of one another. Damrauer used variable light excitation to characterize the ultrafast behavior of Fe­(II) polypyridyl complexes.^89^ They too argued against direct nonradiative passage between excited states, which is very much in line with the parallel model and the data reported here. The heteroleptic nature of **Fe­(Cpy)_2_(deeb)** with two unique charge transfer bands in the visible region allows this novel behavior to be clearly observed. Additional ultrafast spectroscopic characterization will certainly provide a more detailed mechanistic model of excited state relaxation in this interesting complex.

Further support for the assignment of the underlying excited state relaxation processes in **Fe­(Cpy)**_**2**_ **(deeb)** was provided by (scalar-relativistic) TDDFT simulations. Initially, upon excitation within the Franck–Condon geometry (S_0_ equilibrium structure), the population transfer from the accessible ^1^MLCT states (i.e., S_3_, S_4_, S_8_, S_9_, S_14_ and S_18_) to the triplet manifold was investigated. The respective spin–orbit couplings (SOCs) suggest that efficient intersystem crossing (ISC) proceeds through both singlet and triplet MLCT gateway states. Among these states, SOCs of up to 150 cm^–1^ were predicted. Such a magnitude of SOCs is typical for MLCT states in 3d transition metal complexes, while slightly larger couplings are observed for the heavier 4d and 5d analogs.^90–94^ Substantially smaller SOCs are predicted in **Fe­(Cpy)**_**2**_ **(deeb)** between these ^1^MLCT states and other triplet states in energetic proximity (i.e., ^3^IL and ^3^MC states). Notably, upon 400 nm excitation, efficient ISC from the ^1^MLCT_Cpy_ states (S_14_ and S_18_) to the respective ^3^MLCT_Cpy_ states occurs (differing only in the d-orbital occupation). Unfortunately, our attempts to optimize these high-lying ^3^MLCT_Cpy_ states were unsuccessful. Therefore, we focus our computational analysis on the excited state relaxation pathways following excitation into the lowest-energy absorption band at 580 nm and subsequent ISC along singlet and triplet MLCT_deeb_ pathways. Of particular interest was the lowest energy triplet (T_1_) state within the Franck–Condon point, which was consistently predicted by TDDFT, as well as using unrestricted (u)­DFT, to be of ^3^MLCT_deeb_ character; this behavior was further verified with all three utilized functionals. Upon structural relaxation, this state was stabilized from 1.45 eV in the S_0_ structure to 1.25 eV (uDFT)—in full agreement with the respective TDDFT energy in the ^3^MLCT_deeb_ equilibrium (1.32 eV). Notably, the major contribution to this energetic relaxation was related to outer-sphere solvent relaxation, while only minor structural rearrangement occurred within the inner sphere. Within the Franck–Condon structure, two additional ^3^MLCT_deeb_ states (T_2_ and T_3_, only differing in the d-orbital configuration) are predicted to be lower in energy than the lowest energy ^3^MC state (T_4_) at 1.94 eV. Optimization of the lowest energy ^3^MC state (T_4_ in S_0_ geometry) led to a weakened coordination of both Cpy ligands, in particular, the axial Fe–N­(py) bonds to the Cpy ligands elongate from 1.993 within the Franck–Condon geometry (S_0_) to 2.320 Å within the structurally relaxed ^3^MC state.

This pronounced structural alteration—associated with the singly populated σ_z^2^_ * orbital (or d_z^2^_ within a simple ligand field theory picture)—lowers the ^3^MC energy from 1.94 eV (T_4_ within S_0_) to 0.83 eV (T_1_ in ^3^MC) as obtained by uDFT, Figure 2C. The standard B3LYP functional as well as the TPSSh functional predict comparable uDFT energies, while these two hybrid functionals provided an even less reliable description of the ^3^MC at the TDDFT level of theory. Subsequently, we approximated the activation energy associated with the population transfer from the equilibrated lowest energy ^3^MLCT_deeb_ state (at 1.25 eV), obtained upon ISC and subsequent relaxation, to the ^3^MC minimum (at 0.83 eV). To this aim, a linear-interpolated internal coordinate (LIIC) was constructed connecting the two triplet state structures using both uDFT and TDDFT calculations (Figure 2C). Thus, such LIIC accounts for all occurring structural changes, while in case of the present ^3^MLCT_deeb_→^3^MC relaxation the most prominent structural changes are related to the elongation of the axial Fe-Cpy bond lengths. Of note, all structural changes are occurring simultaneously along such LIIC; thus, complex (stepwise) chemical transformations and the associated barriers cannot be described appropriately by means of such interpolated coordinate. Based on the potential energy curves obtained along the LIIC, we approximate the barrier for the ^3^MLCT_deeb_→^3^MC process to ∼0.15 eV. Finally, the TA signatures of both the ^3^MLCT_deeb_ as well as of the ^3^MC states were simulated. According to TDDFT, and in agreement with the time-resolved measurements, the simulated signature of both species displayed a pronounced ground state bleach in the visible region with an excited state absorption feature in the red region. In the ^3^MLCT_deeb_, an intraligand excited state absorbance was predicted in the infrared region (into T_7_). In contrast, the ESA band computed in the range of 650 and 750 nm in the case of the ^3^MC state was associated with a ^3^LMCT_deeb_ transition (T_5_, at 1.35 eV or 916 nm). The major difference between the simulated ^3^MLCT_deeb_ and ^3^MC signatures is the ESA below 400 nm, which was only present in the case of the ^3^MLCT_deeb_ species, see T_35_ and T_37_. Notably, a similar ESA was observed experimentally at early delay times. Thus, the shorter-lived species (2 ps component) was assigned to the ^3^MLCT_deeb_ state, which further decays with a barrier of approximately 0.15 eV to the ^3^MC species with a 20 ps lifetime. Finally, the small energy gap between the equilibrated ^3^MC state and the singlet ground states, i.e., merely 0.22 eV based on (u)­DFT, leads to a rapid excited state deactivation.

### Saponification and Sensitization

Overnight reactions of **Fe­(Cpy)**_**2**_ **(deeb)** and TiO_2_ thin films in acetonitrile solutions yielded only weakly colored materials. Alternative methods for hydrolysis of the ester groups of **Fe­(Cpy)**_**2**_ **(deeb)** were therefore developed and are detailed in the SI.^95^ It is noteworthy that under optimal hydrothermal conditions in water or in the presence of base, a clean conversion of the ester groups into the carboxylic acids was evidenced by ^1^H NMR spectroscopy, showing the complete loss of ethyl ester signals and the appearance of free ethanol in solution (Figures S25 and S26). The conversion of **Fe­(Cpy)**_**2**_ **(deeb)** to **Fe­(Cpy)**_**2**_ **(dcb)** through gentle heating in neat water highlights the remarkable robustness of the central coordination sphere in **Fe­(Cpy)**_**2**_ **(deeb)** (Figure S26). Of interest, harsher conditions are generally necessary to saponify ethyl esters on bipyridyl ligands for complexes of ruthenium.^31^

Further credence for the presence of the dicarboxylic acid complex was provided by proton-responsive absorption spectra.^31^ The addition of excess base revealed a clear blue-shift of the low energy absorption band. The subsequent addition of acid led to a red-shift (Figure S27). These observations were in full agreement with the assignment of the low energy absorption as MLCT to the bipyridine ligand. Deprotonation forms the dianionic dicarboxylate complex, which destabilizes the π* orbitals and increases the HOMO–LUMO gap, leading to a blue-shift of the low energy absorption. Note that a corresponding and smaller spectral shift was also observed for the high-energy absorption, resulting in a measurable shoulder. This observation agrees well with the TDDFT calculations and suggests Fe→deeb transitions also contribute to the low energy flank of the higher energy absorption band.

Sensitization of TiO_2_ with **Fe­(Cpy)**_**2**_ **(dcb)** under optimized conditions resulted in 3 × 10^–8^ mol/cm^2^ surface coverages that corresponded to an absorbance maximum greater than 2, indicating that 99% of the incident light was harvested. A comparative study of the fraction of the light absorbed, i.e., the absorptance spectrum, and the photocurrent action spectrum are described further below. Pulsed 488 nm light excitation of **Fe­(Cpy)**_**2**_ **(dcb)|TiO**_**2**_ in a 0.1 M LiClO_4_ acetonitrile solution resulted in the formation of a millisecond long-lived transient, Figure 5, left. The normalized difference spectra were within experimental error the same indicating that a single state was formed. The spectral data were fully consistent with the formation of an interfacial charge-separated state; i.e., **Fe** ^**II**^ **(Cpy)**_**2**_ **(dcb)|TiO**_**2**_ + hv → **Fe** ^**III**^ **(Cpy)**_**2**_ **(dcb)|TiO**_**2**_ **(e** ^–^). We note that the quasi-reversible electrochemistry measured in electrolyte solutions was also evident for the complex anchored to conductive tin-doped indium oxide (ITO), enabling cyclic voltammetry (Figure S28) and spectroelectrochemical measurements (Figure S29). We also note that the injected electron has a low extinction coefficient in this spectra range.^4^ The transient spectra returned cleanly to baseline on a millisecond time scale. UV–vis absorption spectra measured before and after pulsed laser excitation revealed no evidence for permanent photochemistry which further highlights the remarkable chemical robustness of this iron complex.

**Figure 5:**
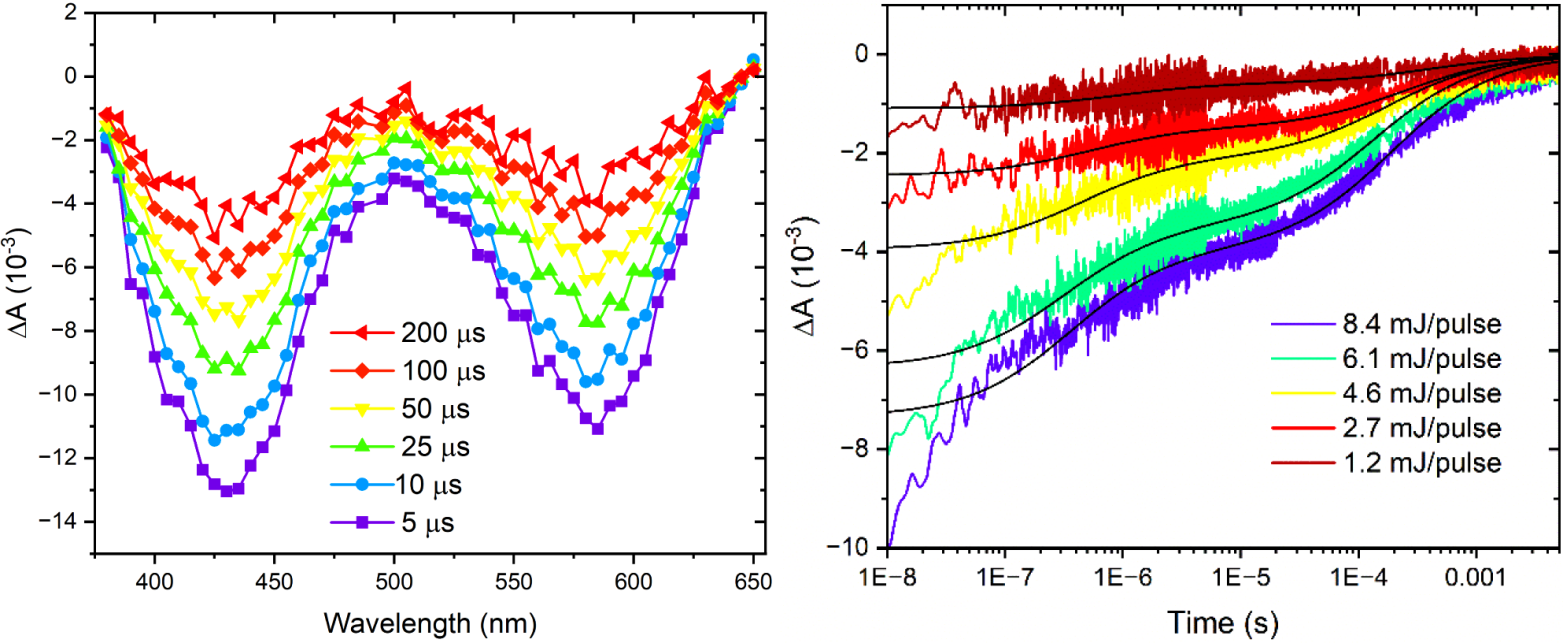
Transient absorption difference spectra measured after pulsed 488 nm light excitation of an **Fe­(Cpy)_2_(deeb)|TiO_2_** thin film submerged in 0.1 M LiClO_4_ acetonitrile (left). The recombination kinetics were monitored at 600 nm as a function of laser fluence (right). Overlaid on this data are fits to a bi-second-order kinetic model, eq 1.

Kinetic data acquired at an observation wavelength of 600 nm were recorded as a function of the 488 nm excitation irradiance. The resulting kinetic data were nonexponential and were well described by a bisecond-order equal-concentration kinetic model (eq 1),^96^ where Δε is the change in extinction coefficient (−4000 M^–1^ cm^–1^ at 600 nm), *l* is the path length (5 μm), ΔA_o_ is the initial amplitude, and A_2_ is the amplitude of the slower component, Figure 5, right. The extracted rate constants, *k*_1_ and *k*_2_ varied by about 3 orders of magnitude and were insensitive to the excitation irradiance, and hence the number of initial charge-separated states formed, within experimental error (Figure S30). Note that conversion of the observed rate constants to units of concentration is nontrivial due to the unknown optical path length in the mesoporous thin films.

1 $$\Delta A\left(t\right)=\frac{\Delta A_{0}-\Delta A_{2}}{1+\left(k_{1}/\Delta \epsilon l\right)t\left(\Delta A_{0}‐\Delta A_{2}\right)}+\frac{\Delta A_{2}}{1+\left(k_{2}/\Delta \epsilon l\right)t\left(\Delta A_{2}\right)}$$

The quantum yield for injection (Φ_inj_) for **Fe­(Cpy)**_**2**_ **(dcb)|TiO**_**2**_ was determined spectroscopically 50 ns after 457, 488, and 532 nm light excitation. It was found that Φ_inj_ = 0.30 ± 0.05 for 457 and 488 nm light excitation, but excitation into the lower energy band with green 532 nm light resulted in a bleach with about half the magnitude, resulting in Φ_inj_ = 0.15 ± 0.02 (Figure 6, left). This behavior was qualitatively consistent with the observed band-selective photocurrent action spectra described below. Details of the procedure used to determine Φ_inj_ can be found in the SI.

**Figure 6:**
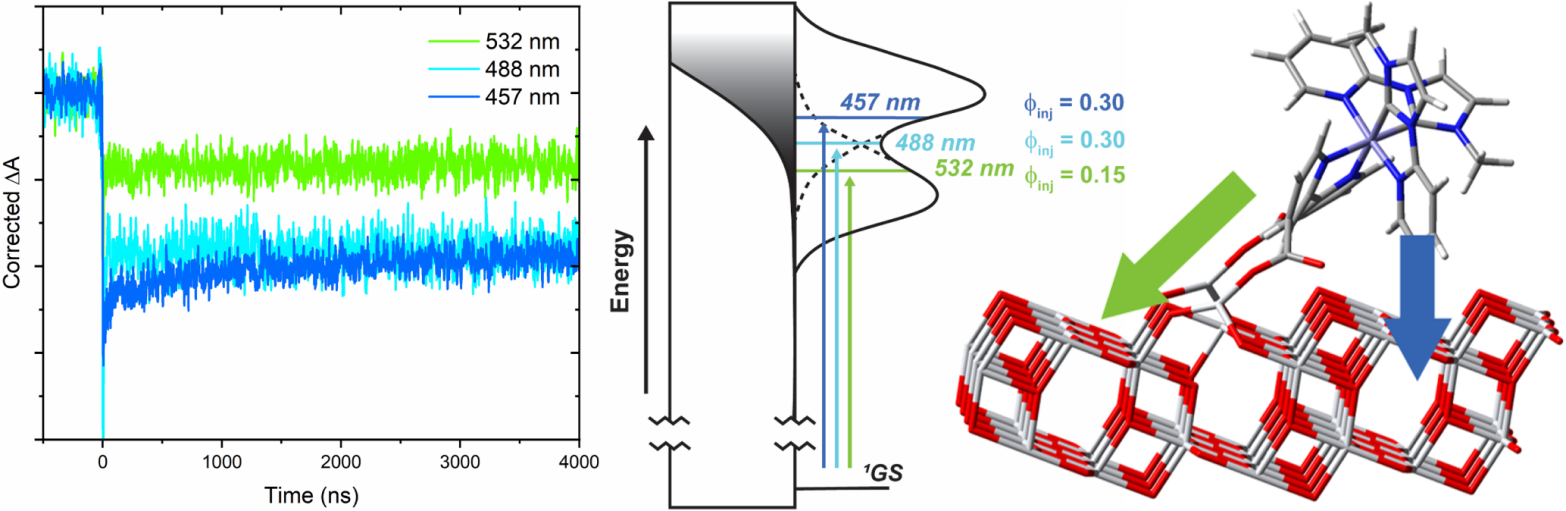
Left: Absorption change monitored at 555 nm after pulsed light excitation of **Fe­(Cpy)_2_(dcb)|TiO_2_** in a 0.1 M LiClO_4_ acetonitrile with 457 nm (dark blue), 488 nm (light blue), 532 nm (green) light. The magnitude of the kinetics was divided by the absorptance of **Fe­(Cpy)_2_(dcb)|TiO_2_** at each indicated wavelength to account for an increase in signal because of greater light absorption. Middle: An energy diagram showing that blue light excitation results in the population of higher-energy excited states that have a better overlap with the acceptor states of TiO_2_ than does green light excitation. The measured injection quantum yield (Φ_inj_) are given for each excitation wavelength. Right: Tight-binding model of **Fe­(Cpy)_2_(dcb)|TiO_2_** on the (101) face of anatase TiO_2_. The arrows shown represent injection from the MLCT_dcb_ (green) or the MLCT_Cpy_ (blue) to the TiO_2_.

### Photoelectrochemical Data

The incident photon-to-current efficiency (% IPCE) was measured as a function of the excitation wavelength, a photocurrent action spectrum, for **Fe­(Cpy)**_**2**_ **(dcb)|TiO**_**2**_ and **Fe­(Cpy)**_**2**_ **(dcb)|SnO**_**2**_ in a 0.05 M LiI, 0.05 M I_2_ acetonitrile electrolyte solution, Figure 7. The sensitized materials were photoexcited through the FTO substrate to minimize competitive light absorption by the electrolyte (Figure S31). Significant steady-state photocurrents were observed that were found to be reproducible and independent of whether the sample was scanned from high energy to low energy or from low energy to high energy. The photocurrent magnitude at 420 nm for **Fe­(Cpy)**_**2**_ **(dcb)|TiO**_**2**_ was about 10-fold larger than that for **Fe­(Cpy)**_**2**_ **(dcb)|SnO**_**2**_. Control experiments with unsensitized SnO_2_ revealed a small contribution from the SnO_2_ that was subtracted from the data provided in Figure 7. The significantly lower photocurrent for SnO_2_ relative to TiO_2_ has previously been shown to result from more rapid recombination of the injected electron with the oxidized complex.^97^ The photocurrent action spectra were in poor agreement with the absorptance spectra. When the amplitude of the IPCE action spectra was normalized to the absorptance measured at 424 nm, the photocurrent measured with excitation of the lower-energy band (580 nm) had a smaller magnitude than was expected based on the fraction of absorbed incident light, Figure 7. The IPCE is lower than the Φ_inj_ values measured spectroscopically, indicating that a fraction of the injected electrons recombine with the Fe^III^ center.

**Figure 7:**
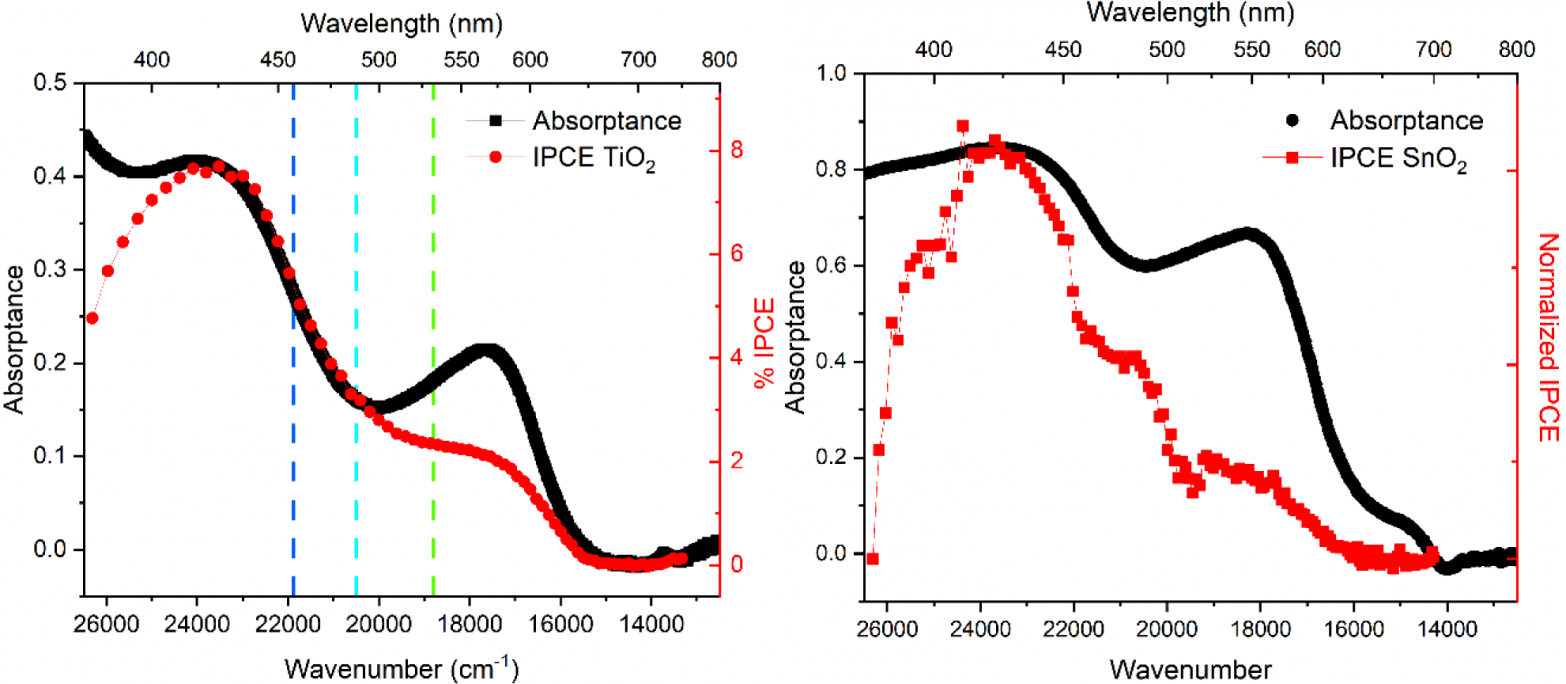
Incident photon-to-current-efficiency (% IPCE) versus the excitation wavelength in a 0.5 M LiI, 0.05 M I_2_ acetonitrile solution (red circles) on TiO_2_ (left) and SnO_2_ (right). Superimposed on this data are the absorptance spectra of the same sensitized thin film submerged in a neat acetonitrile solution (black squares). The dashed lines on the TiO_2_ spectra correspond to the laser irradiation wavelengths used to determine injection quantum yields.

Photocurrent density versus voltage (J-V) plots for **Fe­(Cpy)**_**2**_ **(dcb)|TiO**_**2**_ (black squares) and **N3|TiO**_**2**_ (red circles), where **N3** is *cis*-[Ru­(dcbpy)_2_(NCS)_2_] in a in a 0.5 M LiI, 0.05 M I_2_ acetonitrile solution (black squares) were measured under one-sun (100 mW/cm^2^) AM 1.5 illumination, Figure 8. No attempt was made to optimize the performance of these solar cells with TiCl_4_ treatments, blocking layers, or electrolyte additives.^77,98–100^ Instead, the photoelectrochemical performance of **Fe­(Cpy)**_**2**_ **(dcb)|TiO**_**2**_ was compared to **N3|TiO**_**2**_ in the standard iodide/iodine electrolyte employed for the photocurrent action spectra. A photodiode was used to quantify the fraction of the air-mass 1.5 simulated sunlight absorbed by the chromophore (Figure S32). This fraction was then used to normalize the short-circuit current observed for each sample. It was found that **Fe­(Cpy)**_**2**_ **(dcb)|TiO**_**2**_ exhibited a short-circuit photocurrent that was about 30% that of **N3|TiO**_**2**_. On average the short circuit photocurrent for **Fe­(Cpy)**_**2**_ **(dcb)|TiO**_**2**_ was about one-third that of **N3|TiO**_**2**_ (98 ± 8% for **N3|TiO**_**2**_ and 31 ± 8% for **Fe­(Cpy)**_**2**_ **(dcb)|TiO**_**2**_). First insights with respect to the structure of the surface-immobilized **Fe­(Cpy)**_**2**_ **(dcb)** were obtained by computational modeling at the tight-binding level of theory (see Supporting Information for details). These simulations where both (deprotonated) carboxylic anchoring groups are bound to an anatase (101) surface suggest a prominent leaning of the iron complex onto the semiconductor by means of one **Cpy** ligand. The shortest Cpy-TiO_2_ distances (hydrogen–titanium) are merely 1.882 and 1.859 Å within the singlet and the ^**3**^ **MLCT**_**dcb**_ structures, respectively. Thus, it is tempting to speculate that these short distances provide sufficient electronic coupling for efficient charge injection from the energetically high-lying ^**3**^ **MLCT**_**Cpy**_ states (Φ_inj_ = 0.30) to the anatase TiO_2_.

**Figure 8:**
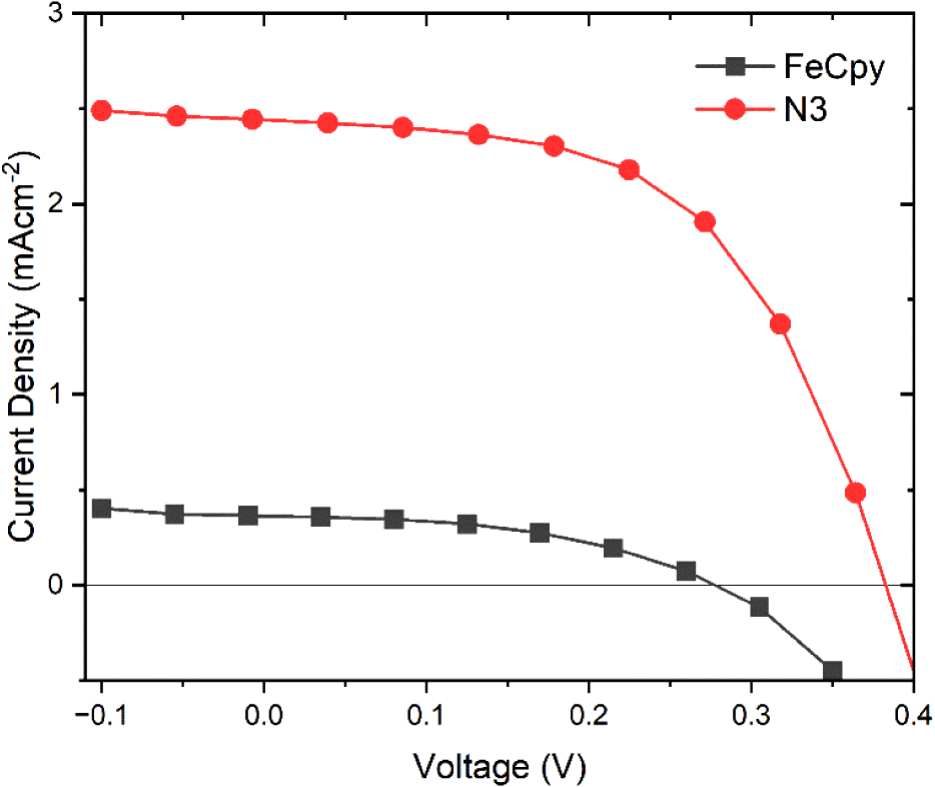
Photocurrent density versus voltage (J-V) plots for **Fe­(Cpy)_2_(dcb)|TiO_2_** (black squares) and **N3|TiO_2_** (red circles) in a 0.5 M LiI, 0.05 M I_2_ acetonitrile solution. The lines connecting the data points are to guide the eye.

## Discussion

This study identified a robust, heteroleptic, iron-based chromophore with a diimine linking ligand that is suitable for use in dye-sensitized solar cells. **Fe­(Cpy)**_**2**_ **(deeb)** converts sunlight into electrical current with about one-third the efficiency of a gold-standard complex *cis*-Ru­(dcb)_2_(NCS)_2_, **N3**. Moreover, **Fe­(Cpy)**_**2**_ **(deeb)** exhibits fully reversible metal- and ligand-based electron transfer, both in solution and when bound to a metal oxide showing high stability in the adjacent one-electron oxidized and reduced states necessary for applications that require oxidative and reductive excited state quenching. The well-resolved MLCT absorption bands allow selective excitation for fundamental photophysical and photoelectrochemical studies. Two particularly interesting aspects of this research deserve further discussion: 1) wavelength-dependent “hot carrier” electron transfer; and 2) the photogeneration of long-lived interfacial charge-separated states using an iron chromophore.

Electron transfer from electronically and/or vibrationally “hot” excited states has direct relevance to exceeding the Schockley-Queisser conversion limit for solar energy conversion discussed in the Introduction section.^10,11^ Consequently, there is continued interest in intercepting hot electronic excited states before they relax to a thermally equilibrated state. In molecular donor–acceptor complexes, hot electron transfer is rare but is known.^14^ At semiconductor interfaces, and particularly dye-sensitized TiO_2_ interfaces,^20^ hot interfacial electron transfer is precedented and the results disseminated herein provide new insights and show that such processes can occur from “remote” excited states that are only weakly coupled to a semiconductor surface.

The long lifetime of the interfacial charge-separated state generated under visible light excitation, **Fe** ^**II**^ **(Cpy)**_**2**_ **(dcb)|TiO**_**2**_ + hv → **Fe** ^**III**^ **(Cpy)**_**2**_ **(dcb)|TiO**_**2**_ **(e** ^–^), is essential for applications in regenerative and photoelectrosynthesis solar cells, where oxidation of a redox mediator or catalyst must compete kinetically with the unwanted recombination reaction. While such long-lived charge separation is well established for benchmark systems like **N3|TiO**_**2**_, rapid (<10 ns) recombination to the oxidized chromophore has been reported to depopulate nearly 90% of the initially formed charge-separated states within 10 ns for iron complexes.^101^ The reason for this remains speculative and may result from the presence of lower lying metal centered electronic states that facilitate rapid geminate recombination. Of note, the highest-efficiency Fe-NHC chromophores previously reported for photoelectrochemical applications are equipped with at least 4 NHC binding moieties;^35,102^ Fe­(Cpy)_2_(dcb)|TiO_2_ provides the first example of photosensitization from an iron complex with only 2 NHC binding moieties. A study investigating the photophysical behavior of iron complexes with NHC ligands can be found in the recent literature.^62^

### Hot Carrier Electron Transfer (Band-Selective Injection)

Evidence for “hot injection” at dye-sensitized TiO_2_ (anatase) surfaces is compelling and can be divided into two broad classifications based on the number of visible absorbance bands. In the first class, variable wavelength light excitation into a *single absorption band* reveals more efficient electron injection on the blue edge of the band than on the red edge, a behavior often attributed to injection from a vibrationally hot excited state. Using azulene, Piotrowiak and coworkers showed that blue light excitation resulted in electron injection that was nine times faster than that measured with lower energy excitation.^21^ Likewise, visible light excitation on the blue edge of the MLCT absorbance of [Ru­(ina)­(NH_3_)_5_]^2+^ (where ina is isonicotinic acid, pyridine-4 carboxylic acid) resulted in an injection yield of 0.30 while lower energy excitation yielded 0.15. Interestingly, the injection yields increased when the amine ligands were deuterated (ND_3_) consistent with an inverse isotope effect of 0.7.^103^

In the second class, the sensitizing complex has two absorption bands and light excitation of the higher energy band results in more efficient injection than does the lower energy band, behavior that has been termed “band selective” injection that is most relevant to this study. The first example of band-selective injection was reported by Ferrere and Gregg with the iron-based chromophore *cis*-Fe­(dcb)_2_(CN)_2_, **Fe-1** in Scheme 1.^36^ Like other complexes with the general structure *cis-*[M­(bpy’)_2_X_2_], two symmetry-allowed MLCT bands were observed in the visible region and light excitation of the higher energy band resulted in an incident photon-to-current efficiency (% IPCE) of 11% while the lower energy band yielded only 2% in regenerative solar cells with the standard iodide/iodine redox mediator. The authors concluded that electron injection from an ultrashort-lived, upper excited state competed kinetically with relaxation to a metal centered state. They assumed that the metal-centered excited state did not transfer an electron to the surface. Interestingly, this same band-selective injection was observed with SnO_2_ which has a conduction band edge that is expected to be ∼500 mV more positive than TiO_2_.^4^ Concerns that coordination of the ambidentate cyano ligand(s) to the TiO_2_ surface might contribute to the higher photocurrent efficiency of the upper excited state^104^ were dismissed after subsequent studies by Jakubikova revealed that such interactions were not significant under the experimental conditions employed.^33,38^ More recently, Wärnmark and coworkers have shown that the higher energy absorption band of **Fe-2** gives rise to about twice the photocurrent of the lower energy band even though they absorb about the same fraction of incident light.^37^

The results disseminated herein indicate some similarities, and an important difference, between this prior research and that reported here for **Fe­(Cpy)**_**2**_ **(deeb).** The photocurrent action spectrum of **Fe­(Cpy)**_**2**_ **(dcb)|TiO**_**2**_ displayed an IPCE of 7% for the higher energy band and 2% for the lower energy band. In qualitative agreement with these photocurrent data, the quantum yields for electron injection measured 50 ns after pulsed laser excitation were twice as large with blue light compared to green excitation. The photocurrent measured with a sensitized SnO_2_ photoelectrode was about 10-fold smaller than that measured on TiO_2_, yet showed a similar band-selective injection. Indeed, for both oxide materials, the photocurrent was most optimal at 400 nm, a wavelength where resonance Raman spectra and TDDFT computational analysis show enhancements of only the Cpy modes. Hence, though the dogma in the DSSC community is that the charge transfer dipole should be oriented toward a ligand directly linked to the surface,^4,105^ the data reported here indicates more efficient hot injection from a ligand remote to the semiconductor surface. While remote injection from long-lived excited states is well documented,^106–108^ this data reveals that electronic coupling between the semiconductor and a remote ligand is sufficient to enable hot interfacial electron transfer before excited state relaxation occurs on the ps time scale. Our initial computational study indicates a prominent leaning of **Fe­(Cpy)**_**2**_ **(dcb)** that positions a Cpy ligand less than 1.9 Å from the semiconductor surface, similar to that of the dcb ligand.

### Long-Lived Interfacial Charge Separation

Ultrafast recombination of the injected electron with the oxidized chromophore is a key drawback noted for iron­(II) complexes relative to those based on organic dyes or second-row transition metal complexes.^101,109^ A long-lived **Fe** ^**III**^ **(Cpy)**_**2**_ **(dcb)|TiO**_**2**_ **(e** ^–^) is needed for efficient oxidation of the redox mediator in regenerative cells and for oxidation of substrates in photoelectrosynthetic cells.^37,77^ For state-of-the-art chromophores (like **N3**) the injection yield is unity and recombination occurs on the milli- to micro- second time scale.^110–112^ In contrast, rapid, picosecond recombination of >80% of the injected electrons has been reported for **Fe-2|TiO**_**2**_ and that fraction that escapes rapid recombination recombine on millisecond time scales.^77,101,113^ The data reported for **Fe-1|TiO**_**2**_ do not provide absolute injection yields, yet appear to indicate that after blue light excitation >50% of the injected electrons recombine within 12 ns and greater than 80% for red light excitation within this time period.^38^ The reason(s) for such rapid recombination are unknown and are the subject to ongoing studies, yet the presence of low-lying metal centered excited states may provide channels for rapid back electron transfer, thereby lowering the yield of useful charge-separated states. In this context, **Fe­(Cpy)**_**2**_ **(dcb)|TiO**_**2**_ has a good quantum yield for excited state injection (Φ_inj_ = 0.3) with a charge-separated lifetime of milliseconds. Importantly, our studies were blind to recombination on the ps time scale and it is possible that a larger concentration of charge-separated states are formed before the time resolution of our instrument.

With regard to the charge recombination mechanism, excited state electron transfer yields an injected electron and an oxidized chromophore in equal numbers. A rate law first-order in injected electrons and first-order in oxidized chromophores, second-order overall, is hence reasonably expected.^114^ The fits of the kinetic data to a second-order model reported here and in previous studies support this expectation.^96^ A sum of two rate constants were needed to fit all the kinetic data, behavior that may result from an underlying distribution of rate constants. Under the experimental conditions utilized, there was no significant recombination over the first 10 μs and a significant fraction of the charge-separated states lived to the millisecond time scale. Proposed mechanisms for charge recombination at any dye-sensitized TiO_2_ interface have progressed over time and a fully robust model is not yet known. At issue is the rate limiting step. Early modeling by Nelson et al. fixed the oxidized chromophore at the injection site and allowed the injected electron to undergo a random walk in the TiO_2_ interior through Ti­(IV/III) trap states.^115^ A time-of-flight model proposed later allowed the injected electrons to access the conduction band before trapping at a Ti­(III) site.^116^ Both these models indicate that the observed recombination kinetics are due to slow transport of the injected electron back to the oxidized chromophore. However, Moia and coworkers have since shown that lateral self-exchange electron transfer, sometimes called “hole hopping” between adjacent chromophores, allows the oxidizing equivalent to be transported away from the injection site.^117^ Chromophores with small self-exchange rate constants and low chromophore surface coverages have been shown to enhance the lifetime of the interfacial charge-separated state.^118,119^ Hence both the transport of the injected electron and the oxidized chromophore are thought to impact the lifetime of the interfacial charge-separated states. Because the TiO_2_ material was kept constant across these studies, it is evident that charge recombination following photoexcitation of this iron complex is comparable to that observed for optimal ruthenium-based complexes.

## Conclusion

The ligand architecture uniquely positions **Fe­(Cpy)**_**2**_ **(deeb)** as a prototype for the systematic study of interfacial photophysics that have so far been dominated by polypyridyl Ru^II^ complexes and stable organic dyes. The two intense absorption bands in the visible region are well formulated as MLCT transitions to the Cpy (420 nm) and deeb (580 nm) ligands. Importantly, the synthetic route reported allows for coordination of any bidentate ligand – a remarkable degree of freedom not found in other useful Fe chromophores. Furthermore, **Fe­(Cpy)**_**2**_ **(deeb)** generates photocurrent equal to that of state-of-the-art iron­(II) chromophores when employed in a DSSC, motivating further studies of this complex in DSSCs under optimized conditions. The MLCT lifetime of 2 ps was shown to be sufficient for hot carrier injection, even though it occurred from a ligand that was not directly linked to the semiconductor surface – behavior that, to our knowledge, has not been previously reported. Indeed, such efficient hot injection stands in sharp contrast with the accepted dogma for thermally equilibrated excited states wherein light-induced charge transfer toward the surface anchoring functional group is viewed as critically important for efficient charge separation. Collectively, the data presented herein contradict this dogma and highlight that energy alignment between the donor excited state and the semiconductor acceptor levels—rather than excited-state lifetime or the spatial position of the excited-state dipole—predominantly governs the efficiency of hot carrier electron transfer relevant to exceeding the Shockley–Queisser limit. Importantly, the lifetime of the charge-separated state with this new iron complex is very similar to that for optimal complexes based on ruthenium.

## Supplementary Material



## Notes

Experimental details on analytical methods (NMR, Mass Spectrometry, XRD) and spectroscopic instrumentation (nanosecond and ultrafast TAS, photoelectrochemical methods); synthesis and characterization of the heteroleptic Fe­(II) chromophore; computational details of DFT and TDDFT calculations using B3LYP, B3LYP10, and TPSSh; kinetic analysis with overlaid fits to experimental data; supplemental spectra (UV–vis) and voltammograms (solution phase and surface bound) (PDF)
