# Supplementary material for: Hot-Carrier Injection and Millisecond Charge Separation from a Robust Heteroleptic Iron(II) Chromophore Immobilized on TiO2
Source: J Am Chem Soc. 2026 May 7;148(19):19643–58. doi: 10.1021/jacs.5c22325 (PMC13195673; doi:10.1021/jacs.5c22325)
Supplement: Supplementary file 1 [file ja5c22325_si_001.pdf]

Supporting Information

Hot-carrier injection and millisecond charge separation from a robust heteroleptic iron(II) chromophore immobilized on TiO_2

Authors:
Thomas Whittemore, Marvin Schmalle, Evgenia Ryndin, Mark Spitler, Elias H. P. Brohmer, Sven Rau, Linda Zedler, Evgeny O. Danilov, Felix N. Castellano, Stephan Kupfer*, Gerald Meyer*, and Dieter Sorsche*

Content

1. Instrumentation and Methods 3
 2.1 Cyclic Voltammetry 3
 2.2 High Resolution Mass Spectrometry (HRMS) 3
 2.3 NMR Spectroscopy 3
 2.4 UV/Vis Spectroscopy 3
 2.5 XRD Measurements 3
 2.6 Computational Details 3
 2.7 Resonance and non-resonant Raman Spectroscopy 5
 2.8 Transient Absorption Spectroscopy 6
 2.9 Photoelectrochemistry 7
  Solar Cell Assembly 7
  Photoelectrochemical Measurements 7
  Solution Spectroelectrochemistry 7
  Thin Film Spectroelectrochemistry 7
 2.10 Quantum Yield of Injection Measurements 7
2. Synthesis 8
 3.1 General Methods and Materials 8
 3.3 Fabrication of Slides 8
 3.4 Synthesis of $Fe(Cpy)_2(deeb)$ 8
 3.4 Synthesis of Ru-1 9
 3.5 Mass Spectrometry 13

3.6 Crystallography 14
3. Cyclic Voltammetry 18
4. Steady-state UV/Vis Spectroscopy 20
5. Computational Results 21
5.1 B3LYP 21
5.2 B3LYP10 27
5.3 TPSSh 35
6. Raman Spectroscopy 40
7. TAS Excited-State Lifetime Kinetic Fits 41
8. Solution Spectroelectrochemistry 41
9 Deprotection of deeb 41
9.1 Method 1 41
9.2 Method 2 42
10. Solution Phase Spectroelectrochemistry 43
11. ITO Electrochemistry 44
12. ITO Spectroelectrochemistry 44
13. TAS Fluence Dependent Recombination Rate Constants 45
14. DSSC illustration 45
15. Photodiode Illustration 46
16. References 47

1. Instrumentation and Methods

2.1 Cyclic Voltammetry

All solution measurements were performed in a nitrogen glove box at room temperature with a scan rate of 100 mV/s. 10 mL of a 0.1M $[nBu_4N][PF_6]$ solution was used as the electrolyte with an analyte concentration of 1 mM. A silver wire inside a frit was used as the reference electrode, a platinum wire was used as the counter electrode and glassy carbon electrode was used as the working electrode.

2.2 High Resolution Mass Spectrometry (HRMS)

HRMS was performed in the mass spectrometry service department of Ulm University. For MALDI measurements this was done using a Fourier Transform Ion Cyclotron Resonance (FT-ICR) mass spectrometer solariX (Bruker Daltonics) equipped with a 7.0 T superconducting magnet and interfaced to an Apollo II Dual ESI/MALDI source. For all MALDI measurements trans-2-[3-(4-tert-butylphenyl)-2-methyl-2-propenylidene]malononitrile (DCTB) was used as the matrix. Spectra were analyzed with Compass Data Analysis Viewer Version 4.4.

2.3 NMR Spectroscopy

NMR spectroscopy was performed either on a Bruker Avance 600 MHz or Bruker Avance NEO 400 MHz spectrometer. The shift values are given in ppm and are referenced to the corresponding solvent residual peaks.

2.4 UV/Vis Spectroscopy

Steady state UV-vis absorption spectroscopy was measured on a Horiba Duetta with EzSpec device placed in a nitrogen atmosphere glovebox. Optical quartz glass cuvettes with a pathlength of 10 mm were used.

2.5 XRD Measurements

Crystals suitable for X-ray crystallography were mounted using a MicroLoop and Perfluoropolyalkyl ether (viscosity 1800 cSt). X-ray diffraction intensity data were measured at 150 K on a Bruker D8 Quest single crystal diffractometer with a PHOTON II detector using Mo - Kα radiation (wavelength $\lambda = 0.71073$ Å). Structure solution and refinement was carried out using the SHELXL package[1, 2] via Olex2. Corrections for incident and diffracted beam absorption effects were applied using multi-scan refinements. Structures were solved by direct methods and refined against F^2 by the full-53 matrix least-squares technique. The hydrogen atoms were included at calculated positions with fixed thermal parameters. All non-hydrogen atoms were refined anisotropically unless otherwise mentioned. MERCURY was used for structural representations.[3]

2.6 Computational Details

If not stated otherwise, quantum chemical simulations were performed using the Gaussian 16[4] package to investigate structural and electronic properties of **Fe(Cpy)$_2$(deeb)**, while the computational focus was set exclusively on its C_2 isomer. Initially, the fully relaxed equilibrium structure of the singlet (S_0) and triplet (T_1) ground state structures were obtained at the density functional level of theory without applying symmetry restrictions. In case of triplet multiplicity, two T_1-equilibrium structures were obtained, namely for the lowest energy triplet metal-to-

ligand charge transfer ($^3MLCT_{deeb}$) state as well as for the lowest energy triplet metal-centered (^3MC) state. Furthermore, the lowest energy quintet state, i.e. of ^5MC character, was fully relaxed. Initial structures were adapted from our recent joint synthetic-spectroscopic-theoretical investigation on a structurally related iron(II) N-heterocyclic carbene complex.[5] All density functional theory (DFT) simulations were performed using three hybrid functionals with medium to low amount of Hartree-Fock exchange, namely using the standard B3LYP[6, 7] functional (with 20% exact exchange), the so-called B3LYP10 functional (a modified version of B3LYP with 10% exact exchange) as well as employing the TPSSh[8, 9] functional. All DFT calculations were carried out in combination with the all-electron def2-SVP basis set and including D3 dispersion correction with Becke-Johnson damping.[10] Implicit solvent effects (acetonitrile: ε = 35.688) were taken into account on the ground state properties by the solute electron density (SMD) variant of the integral equation formalism of the polarizable continuum model (equilibrium procedure).[11, 12]

Subsequently, a vibrational analysis was carried out for each optimized ground state structure (i.e., S_0 and T_1) to verify that a minimum on the 3N-6-dimensional potential energy (hyper-)surface (PES) was obtained.

In the following, the Franck-Condon photophysical properties of the Fe(II) complex were investigated by means of time-dependent DFT (TDDFT). To this aim, the 100 lowest singlet-singlet transitions as well as the 50 lowest spin-forbidden singlet-triplet transitions were obtained within the DFT-optimized S_0 geometries. Therefore, the same XC functional (i.e. B3LYP, B3LYP10 and TPSSh) and basis set as for the preliminary ground state calculation was employed. Our computational focus was set on the $^{1/3}MLCT$ transitions from the iron to the aromatic ligands as well as on $^{1/3}MC$ (metal-centered) states of the $3d^6$ metal ion. Hybrid functionals with medium to low amount of exact exchange are capable of providing a balanced description of locally excited states, e.g. MC and intra-ligand states, as well as of charge transfer states, e.g. MLCT, ligand-to-metal charge transfer (LMCT) and ligand-to-ligand charge transfer (LLCT) states, of 3d transition metal complexes as benchmarked with range-separated and meta-GGA functionals and multiconfigurational methods.[5, 13-16] The non-equilibrium procedure of solvation was applied for the calculation of the excitation energies within the Franck-Condon. This procedure is well adapted for processes where only the fast reorganization of the electronic distribution of the solvent is important.

To evaluate scalar-relativistic effects and their impact on the ISC pathways within the Franck-Condon region, scalar-relativistic TDDFT calculations were performed utilizing Orca 6.0.1[17] using the scalar-relativistic zeroth-order regular approximation (SR-ZORA). DFT and TDDFT calculations were performed exclusively using the B3LYP10 XC functional. All other atoms were described using the respective def2-TZVP basis sets (with the corresponding SARC/J auxiliary basis set).[18] The 50 lowest singlet-singlet and singlet-triplet excitations were obtained, while SOCs were obtained at the SR-ZORA-TDDFT level of theory. The effects of interaction with acetonitrile were taken into consideration at the CPCM level of theory.

Additionally, the excited state properties were evaluated within the relaxed triplet ground state structures, i.e. the lowest energy relaxed $^3MLCTdeeb$ and ^3MC character, as well as stemming from the lowest energy 5MC state of $Fe(Cpy)_2(deeb)$. Once more, all subsequent simulations have been performed in parallel using the B3LYP, B3LYP10 and TPSSh functionals. To this aim, the 200 lowest spin and dipole-allowed triplet-triplet transitions were simulated at the TDDFT level of theory to model the excited state absorption as measured by means of transient absorption (TA) spectroscopy. TA spectra were predicted as difference spectra based on the excited state absorption (ESA) stemming from triplet-triplet simulation (within

^{3}MLCTdeeb and ^{3}MC equilibria, T1) and the singlet-singlet transitions within the Franck-Condon point (i.e. ground state bleach, GSB). In a similar fashion, the TA signature associated with the 5MC state was obtained in combination with GSB from the Franck-Condon transitions. Thereby, a relative population of 1:1 was assumed for the excited state vs. the ground state species. This procedure was applied previously and is capable to model the TA spectra at long delay times.[14, 19-21]

Furthermore, the potential energy landscape connecting the fully relaxed ^{3}MLCTdeeb and ^{3}MC photo-intermediates was investigated along linear-interpolated internal coordinates (LIICs). Along the LIIC, energies of the singlet ground state (S_0) as well as of the triplet states of interest were predicted based on restricted and unrestricted DFT as well as based on TDDFT (singlet-triplet excitations). Such comparably simple LIICs typically provide a suitable description in case of intra- and intermolecular electron and energy transfer processes, where minor structural rearrangements between the equilibrated structures occur.[21-28] However, a drawback of linear-interpolated coordinates is that all structural changes are forced to occur simultaneously and not sequentially. Noteworthy, the B3LYP10 functional provides the best alignment between the unrestricted DFT and TDDFT description of the lowest energy triplet state upon stretching of the coordination environment (see Figures S16, S18 and S21). Therefore, and while all three functionals provide qualitatively a fully consistent picture of the photophysics of **Fe(Cpy)$_2$(deeb)**, we decided to focus on the results obtained by means of the B3LYP10 functional within the main text.

Finally, the doubly deprotonated **Fe(Cpy)$_2$(dcb)** was immobilized by both carboxylate anchoring groups onto an anatase (101) surface in order to obtained initial insights regarding the structural properties at the dye-semiconductor interface, while the anatase (101) surface is typically considered the most stable surface.[29] To model such TiO_2 surface the atomic simulation environment (ASE)[30] was utilized; based on the generated cell an anatase cluster was extracted. In particular, the dimensions of anatase cluster were chosen *i)* to allow immobilization of the **Fe(Cpy)$_2$(dcb)** complex while allowing an additional interaction of the **Cpy** ligands with surface, *ii)* to avoid vacancies at cluster's edges near the binding sites, *iii)* to yield a low total charge of the cluster (i.e. balancing Ti^{4+} and O^{2-}), and, *iv)* by restricting the number of atoms in order to lower the computational demand. This resulted in a $[Ti_{52}O_{102}]^{4+}$ cluster with an anchored **Fe(Cpy)$_2$(dcb)** as shown in Figure 6C. The structure was relaxed at the GFN0-xTB level of theory in gas phase within singlet as well as triplet Orca 6.0.1[17, 31, 32]. The anatase cluster was kept frozen except for the two Ti atoms connected to the carboxylate groups. A frequency analysis was carried out and verified that local minima structures were obtained.

Both, the immobilized singlet as well as the triplet species feature partially twisted **dcb** ligand with dihedral angles of $\angle_{NCCN}$ = 22.7° and 17.0°. Thereby, the partial planarization of the **dcb** ligand from the singlet to the triplet species reflects the $^3MLCT_{dcb}$ configuration with a singly occupied π^*_{dcb} orbital.

All calculated equilibrium structures as well as high resolution images (charge density differences and spin densities) are available from the free online repository Zenodo.[33]

2.7 Resonance and non-resonant Raman Spectroscopy

Resonance Raman (rR) spectra were obtained using a single longitudinal mode diode laser at 405 nm (TopMode-405-HP, Toptica, Germany), a 473 nm and a 532 nm diode-pumped solid-state laser (HB-Laser, Germany). Raman scattered light was collected with an IsoPlane 160

spectrometer (Princeton Instruments, USA) equipped with gratings of 2400, 1200, and 600 grooves/mm and a 30 μm entrance slit, ensuring high spectral resolution. Long-pass optical filters (Semrock, USA) matched to the excitation wavelengths were used to selectively detect Raman signals and suppress Rayleigh scattering. The laser power at the sample position was maintained at 5 mW to minimize thermal effects and prevent sample degradation. Acetonitrile served as an internal standard, with its characteristic Raman band at 1370 cm^{-1} used to calibrate both the Raman shift axis and the relative intensity of the spectra. For spectral processing, rR spectra were background corrected, and the solvent spectrum was subtracted.

Nonresonant FT-Raman spectra (λ_{exc} = 1064 nm) of the solid homoleptic $[Fe(Cpy)_2(NCMe)_2]^{2+}$ complex which serves as reference compound were recorded using a MultiRAM (Bruker) with a fiber-coupled diode pumped solid-state laser (DENICAFC LC-3/ 40, KLASTECH-Karpushko Laser Technologies). The laser power was maintained at 10 mW. Each spectrum was obtained by averaging 500 scans. A single IR 352 objective with a 16 mm working distance was used to focus the laser on the sample and collect the scattered light. The signal was detected using a nitrogen-cooled Ge-diode detector (Bruker D418-T), with a spectral resolution of 4 cm^{-1}.

2.8 Transient Absorption Spectroscopy

Transient absorption spectroscopy with nanosecond time resolution was performed in 1 cm path length quartz cuvette. Pulsed excitation was accomplished using a RADIANT X30 tunable laser system: Q-switched, pulsed (10Hz) Nd:YAG laser (Quantel laser By Lumibird, Q-smart 450mJ) tripled to 355 nm, coupled with an OPO module (410–2500 nm tuning range). The laser fluence at the samples was, unless stated otherwise, 3 mJ/pulse (488 nm) at 10 Hz. A 450 W xenon arc lamp aligned perpendicular to the laser served as the probe beam. Two shutters placed between the arc lamp/laser and the sample were opened in 30 ms intervals to limit PMT fatigue and sample degradation. The probe light was focused onto the sample, collimated after the sample, and focused onto an Oriel Cornerstone 260 monochromator optically coupled to a Hamamatsu R928 photomultiplier tube. The transient signal was recorded with a Teledyne Lecroy Wavesurfer 4024HD, 200 MHz digital oscilloscope (with variable bandwidth filters to improve the signal-to-noise ratio). The laser flashlamps, Q-switch, shutters, and oscilloscope trigger delays were controlled with a Berkley Nucleonics Corp. Model 577 Digital Delay Generator. The overall instrument response time was ~15 ns. Single-wavelength kinetics were generated as the average of 150 laser shots to achieve adequate signal-to-noise ratios. Transient absorption spectra measured over a 380–700 nm range were generated from single-wavelength measurements taken every 5 nm.

Ultrafast TA measurements were performed at the North Carolina State University Imaging and Kinetic Spectroscopy (IMAKS) laboratory using a Helios transient absorption spectrometer (Ultrafast Systems).[34] Briefly, the beam from a 1 kHz Ti:sapphire Coherent Libra regenerative amplifier (4 mJ, 100 fs (fwhm) at 800 nm) was split into a pump and a probe beam. The pump beam was directed into an OperA Solo parametric amplifier (Coherent) to generate a 405 nm excitation (pump) source. The residual portion of the 800 nm beam was delayed in a 6.6 ns optical delay stage (Newport) before being focused into a CaF_2 crystal to generate a white light continuum (350-750 nm). The pump (800 μm diameter) and probe (200 μm) beams were focused onto the center of the surface functionalized ITO film, rotated 45° with respect to the probe beam.

2.9 Photoelectrochemistry

2.9.1 Solar Cell Assembly

A sandwich cell was used for all photoelectrochemical measurements. The working photoelectrode was made with TiO_2 nanoparticles dyed with **Fe(Cpy)$_2$(deeb)** bound to FTO glass. The counter photoelectrode was a platinum-coated piece of FTO. Counter electrodes were fabricated in house; two small holes were drilled into the FTO glass to allow a capillary for electrolyte to be added to the cell and the platinum was doped on the slide by coating the FTO with a minimal amount 30 mM solution of H_2PtCl_6 and heating at 380 degrees Celsius for 30 minutes. A 60 micron thick piece of Meltonix by Solaronix with a 0.29 cm^2 hole punched out of it was placed between the two slides and heated at 130 degrees Celsius for 8 minutes to seal the cell. 0.5 M LiI/I_2 in acetonitrile was used as the redox electrolyte.

2.9.2 Photoelectrochemical Measurements

A two-electrode setup was used to measure photoelectrochemical data for assembled dye-sensitized solar cells with **Fe(Cpy)$_2$(deeb)** employed as the dye. An Oriel Cornerstone 260 Monochromator by Newport was optically coupled to a 100 mW xenon white light lamp. Light intensity was detected by a Thor Labs DET100A2 biased detector photodiode array. The generated current was detected by a Gamry Interface 1010B potentiostat connected to a Teledyne Lecroy WaveSurfer Oscilloscope. The reported spectra were held to a 0 V potential for all measurements

2.9.3 Solution Spectroelectrochemistry

A Pine honeycomb cell was used for all solution-phase spectroelectrochemical measurements. A Gold honeycomb spectroelectrochemical electrode was used as both the working and counter electrode. The reference electrode was a Ag/Ag^+ pseudoreference. All experiments were performed in 0.1 M $LiClO_4$ acetonitrile.

2.9.4 Thin Film Spectroelectrochemistry

A Pine WaveNow potentiostat was coupled to an Avantes Avalight-DHc light source with attached Avantes Starline AvaSpec-2048 detector for spectroelectrochemical measurements of **Fe(Cpy)$_2$(deeb)|ITO**. All experiments were perfomred in a 0.1 M TBAPF6 acetonitrile electrolyte. The dyed substrate was the working electrode, a platinum mesh was the counter electrode, and a Ag/Ag^+ pseudoreference was used and referenced to the $Fc^{0/+}$ potential and converted to NHE.

2.10 Quantum Yield of Injection Measurements

Comparative actinometry was carried out using Ru(bpy)$_2$(POEt$_3$bpy)|TiO2 (RuP) as the actinometer (ϕ_{inj} = 1). The observed bleach for RuP can be used to calculate the number of photons the complex absorbed.

$$S1)\ \ \Phi_{inj} = \Phi_{ref}\frac{\frac{\Delta A_{Sample}}{\Delta \varepsilon_{Sample}(1-10^{-A_{Sample}})}}{\frac{\Delta A_{Act}}{\Delta \varepsilon_{Act}(1-10^{-A_{Act}})}}$$

Equation S1 was used to calculate ϕ_{inj} for **Fe(Cpy)$_2$(deeb)|TiO$_2$.** ΔA_{Sample} and ΔA_{Act} define the magnitude of the observed bleach for **Fe(Cpy)$_2$(deeb)|TiO$_2$** and RuP, respectively. These values were extracted from nanosecond TAS at 50 ns (the earliest point of time

resolution on our instrument). $\Delta\varepsilon_{Sample}$ and $\Delta\varepsilon_{Act}$ define the delta molar extinction coefficient at the wavelength observed for **Fe(Cpy)$_2$(deeb)|TiO$_2$** and RuP, respectively ($\Delta\varepsilon$Sample = -4500 $M^{-1}cm^{-1}$ at 555 nm and $\Delta\varepsilon$Act = -10000 $M^{-1}cm^{-1}$ at 450 nm). The $\Delta\varepsilon$ for **Fe(Cpy)$_2$(deeb)** was determined using solution-phase spectroelectrochemistry and $\Delta\varepsilon$ for RuP was determined elsewhere.[35] The quantity ($1-10^{-A}$) for both the sample defines the absorptance of each species and is extracted from the UV-Vis spectrum.

2. Synthesis

3.1 General Methods and Materials

If not otherwise stated, all solvents and reagents were purchased from commercial providers in 95% purity or greater and used as received. Dry solvents used for inert synthesis and spectroscopy were purchased from ACROS Organics in 99.9%+ purity, transferred into an N2-flushed glovebox and used without further purification. Inert manipulations were carried out in an MBraun glovebox workstation equipped with a -40°C freezer and flushed with N_2 in 5.0 quality purchased from mti and dried through a drierite™ column. All precursors, namely the ligand precursor HCpyPF$_6$ chromophore precursors [Fe(Cpy)$_2$(NCMe)$_2$](PF$_6$)$_2$,[5] and [Ru(tbbpy)$_2$(Cl)$_2$] as well as reference chromophore [Ru(tbp)$_2$(deeb)](PF$_6$)[36-38] were prepared following previously reported procedures.

3.3 Fabrication of Slides

Fluorine-doped tin oxide (FTO) glass was sourced from Hartford Glass Company. Nanoparticle suspensions of titanium dioxide (TiO_2)[39], tin oxide (SnO_2)[40], and tin-doped indium oxide (ITO)[41] were prepared according to literature procedures. Nanoparticle suspensions were cast to the FTO via doctor blading. The paste was dried in air for 2 hours before sintering in a tube furnace under an oxygen atmosphere at 450 degrees Celsius for 1 hour. The heated slides were cooled to room temperature gradually and stored in an oven at 130 degrees Celsius until use. Slides were dyed overnight in solutions of the desired chromophore and rinsed with acetonitrile before use.

3.4 Synthesis of Fe(Cpy)$_2$(deeb)

The synthesis of complex **C1** was carried out under argon atmosphere. The precursor **P1** (111 mg, 0.149 mmol, 746.26 g mol^{-1}) and ligand deeb (49 mg, 0.163 mmol, 300.31 g mol^{-1}) were placed in a round-bottom flask as solids. Acetone (30 mL) was added dropwise to the reaction mixture under vigorous stirring, causing the mixture to turn black immediately. The resulting suspension was stirred overnight at room temperature. The solvent was removed under reduced pressure, and the resulting black solid was purified by layering crystallisation. For this purpose, the product was dissolved in acetonitrile and carefully overlaid with diethyl ether. After two days, dark greenish-black crystals were isolated by filtration through a glass frit. The crystals were washed with diethyl ether and allowed to air-dry.

Yield: 83.4 mg (58.13 %)

Mass spectrometry m/z: 337,10200 ([Fe(Cpy)$_2$(deeb)]$^{2+}$)

^{1}H NMR (600 MHz, CD_3CN) δ(ppm): **N,N-axial:** 9.07 (s, 2H), 8.34 (d, 2H), 8.16 (d, 2H), 7.93 (d, 2H), 7.87 (t, 2H), 7.93 (d, 2H), 7.32 (d, 2H), 6.92, (dt, 2H), 6.85 (m, 2H), 4.47 (q, 2H, -CO$_2$C*H$_2$*CH$_3$), 2.75 (s, ImC*H$_3$*), 1.94 (q, -CO$_2$CH$_2$C*H$_3$*) ; **C,N-axial** 9.03 (s, 1H), 8.81 (s, 1H), 8.23 (d, 1H), 8.13 (m, 2H), 8.01 (2H), 7.93 (m, 1H), 7.89 (1H), 7.87 (m, 1H), 7.83 (d, 1H), 7.53

(d, 1H), 7.51 (d, 1H), 7.35 (1H), 7.24 (t, 1H), 7.20 (d, 1H), 7.08 (t, 1H), 7.86 (m, 1H), 4.48 (q, 2H), 4.41 (q, 2H), 1.43 (t, 1H), 1.37 (t, 1H); **C,C-axial:** 8.81 (s, 2H), 8.23 (t, 2H), 8.01 (t, 2H), 7.89 (m, 2H), 7.61 (dd, 2H), 7.53 (m, 2H), 7.40 (d, 2H), 7.34 (d, 2H), 7.15 (m, 2H), 4.44 (q, 4H), 1.42 (t, 6H).

^{13}C NMR (400 MHz, CD_3CN) δ (ppm): **N,N-axial:** 200.5, 164.6, 159.8, 157.8, 156.0,153.0, 140.7, 129.6, 127.4, 124.0, 123.5, 118.8, 112.8, 63.6, 35.94, 14.42; **C,N-axial:** 204.0, 202.2, 164.8, 164.4, 160.5, 159.2, 158.9, 157.0, 155.3, 155.0, 153.7, 152.4, 151.5, 141.9, 140.8, 139.3, 129.8, 129.5, 127.0, 125.8, 124.8, 123.9, 123.3, 119.6, 119.0, 113.0, 63.6, 63.4, 36.9, 36.3, 14.4, 14.4; **C,C-axial:** 203.1, 164,7, 164.3, 154.9, 141.7, 140.6, 139.9, 125.4, 124.7, 123.1, 118.7, 113.5, 113.3, 63.4, 36.3, 14.4.

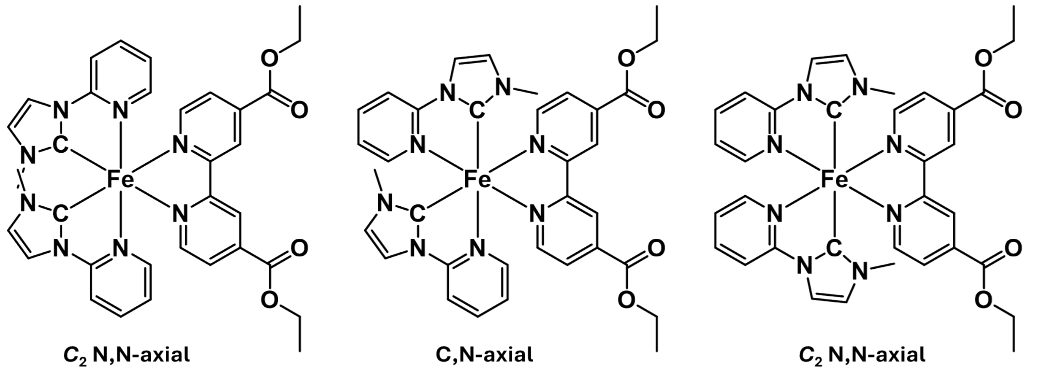

Figure S 1: ChemDraw representation of possible isomers of **Fe(Cpy)$_2$(deeb)**.

3.4 Synthesis of Ru-1

The ruthenium precursor [Ru(dtb)$_2$Cl$_2$] (190 mg, 0.269 mmol, 707.78 g mol^{-1}) and ligand deeb (90 mg, 0.300 mmol, 300.13 g mol^{-1}) were dissolved in a 1:1 mixture of ethanol and water (70 mL) mixture and heated at reflux for 8 h. During this period, the solution gradually changed color from deep purple to red-orange. All volatiles were subsequently removed under reduced pressure and the residue taken up in ethanol. An aqueous solution of NH_4PF_6 (10 mM) was prepared and added dropwise under stirring to the solution of the complex. The resulting suspension was filtered through a glass frit and washed with water (2 x 10 mL) and diethyl ether (2 x 10 mL). The resulting brownish-red powder was dissolved in acetonitrile and carefully layered with diethyl ether. Within a week, crystallization afforded well-formed crystals which were isolated by filtration and washed with diethyl ether (3 x 20 mL). Yield: 200.5 mg (60.91%)

1H-NMR (ACN-d3, 400 MHz, 298 K): δ (ppm): 9.05 – 9.04 (d, 1H), 8.53 – 8.49 (dd, 2H), 7.94 – 7.92 (d, 1H), 7.88 – 7.85 (dd, 1H), 7.58 – 7.54 (m, 2H), 7.48 – 7.45 (dd, 1H) 7.39 – 7.36 (dd, 1H) 4.52 – 4.42 (m, 2H, CH_2), 1.46 – 1.41 (m, 21H, CH_3)

Mass spectrometry m/z: 469.20281 ([Ru(dtb)$_2$(deeb)]$^{2+}$)

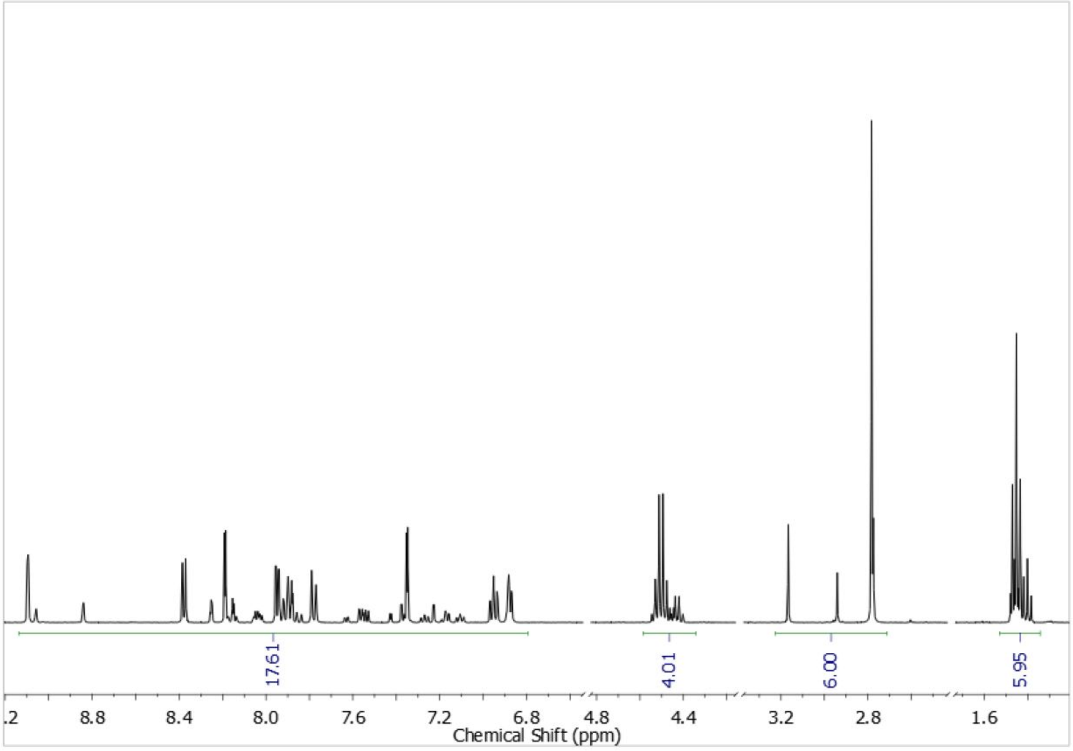

Figure S 2: ^{1}H-NMR of **Fe(Cpy)$_2$(deeb)** in ACN-d$_3$, full spectrum.

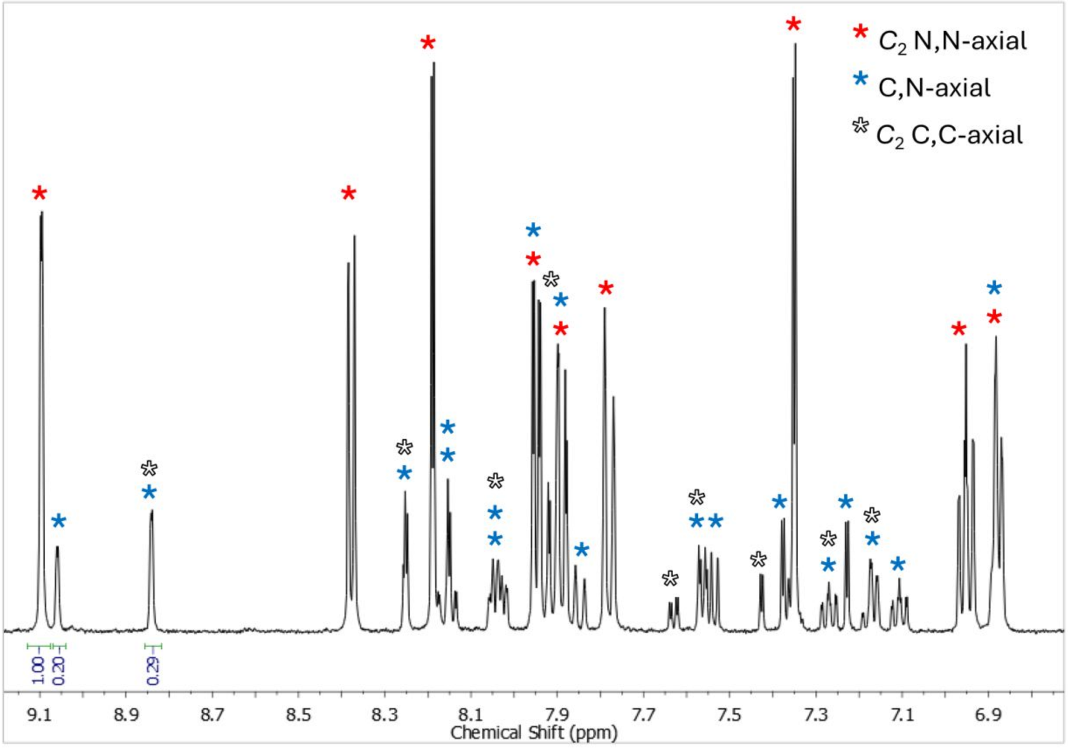

Figure S 3: ^{1}H-NMR of **Fe(Cpy)$_2$(deeb)** in ACN-d$_3$, low-field resonances corresponding to aromatic protons.

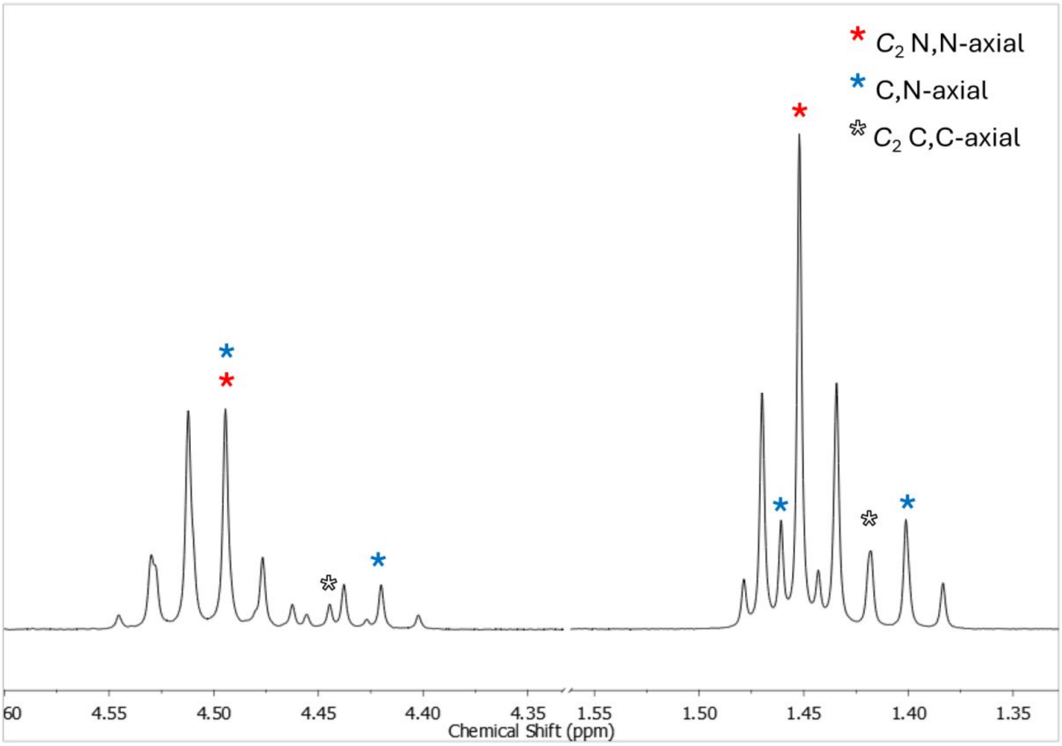

Figure S 4: ^{1}H-NMR of **Fe(Cpy)$_2$(deeb)** in ACN-d$_3$, aliphatic resonances corresponding to ethyl ester protons.

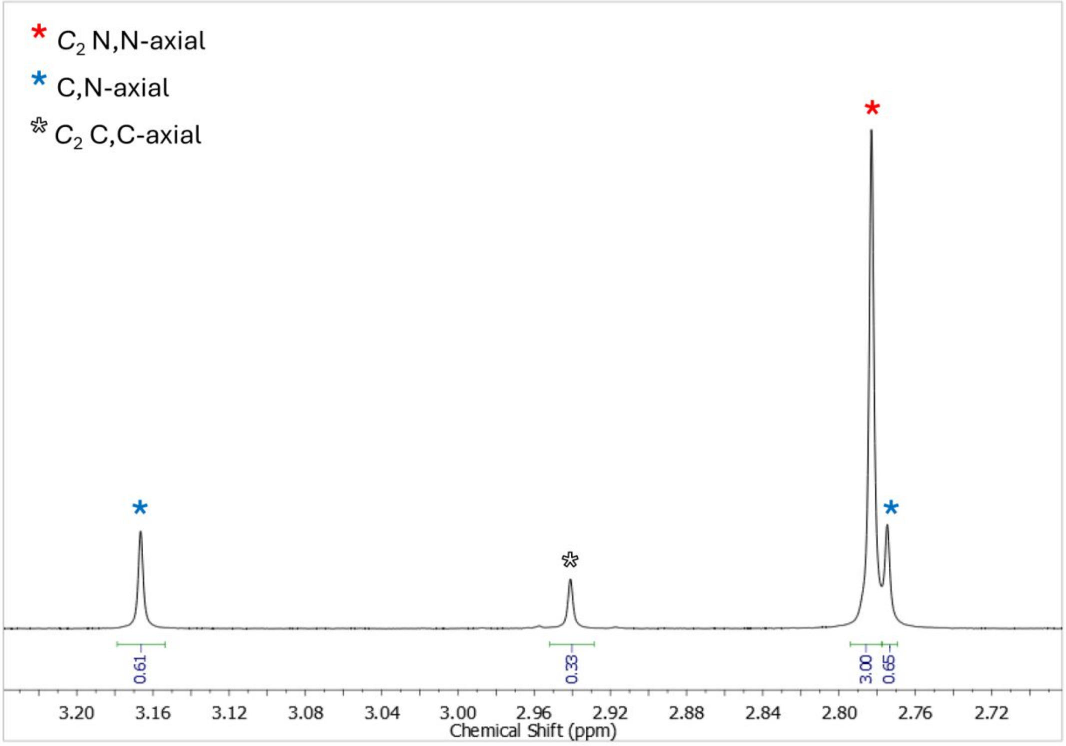

Figure S 5: ^{1}H-NMR of **Fe(Cpy)$_2$(deeb)** in ACN-d$_3$, aliphatic protons corresponding to methylimidazole.

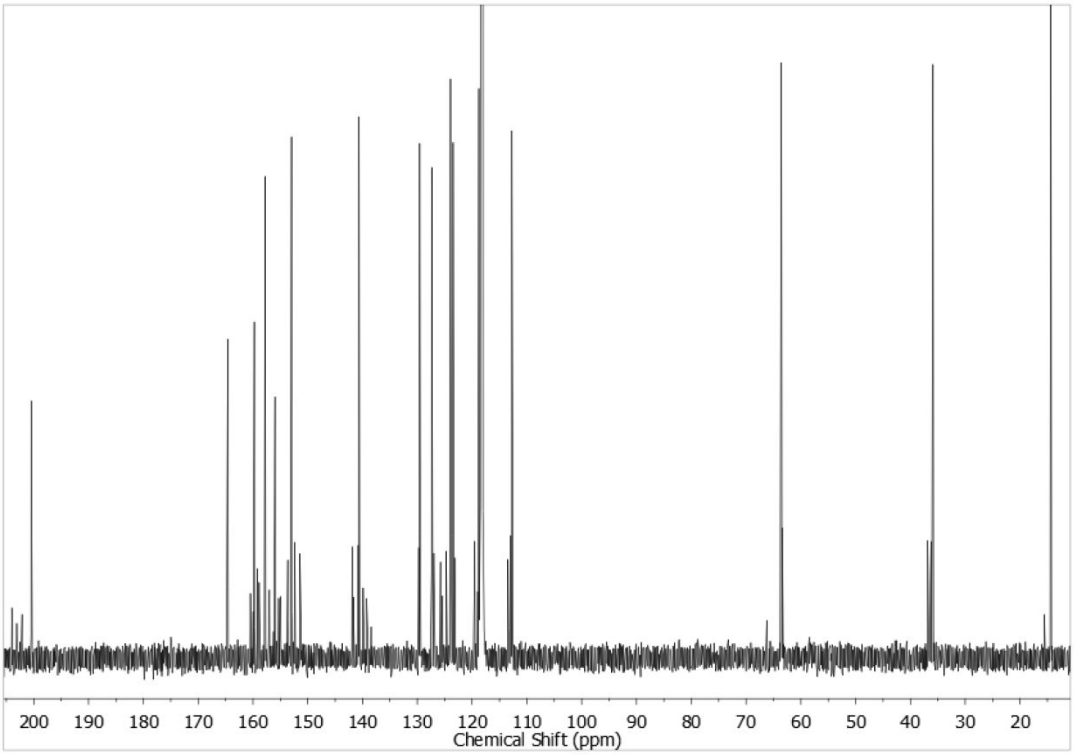

Figure S 6: ^{1}H-NMR of **Fe(Cpy)$_2$(deeb)** in ACN-d$_3$, full spectrum.

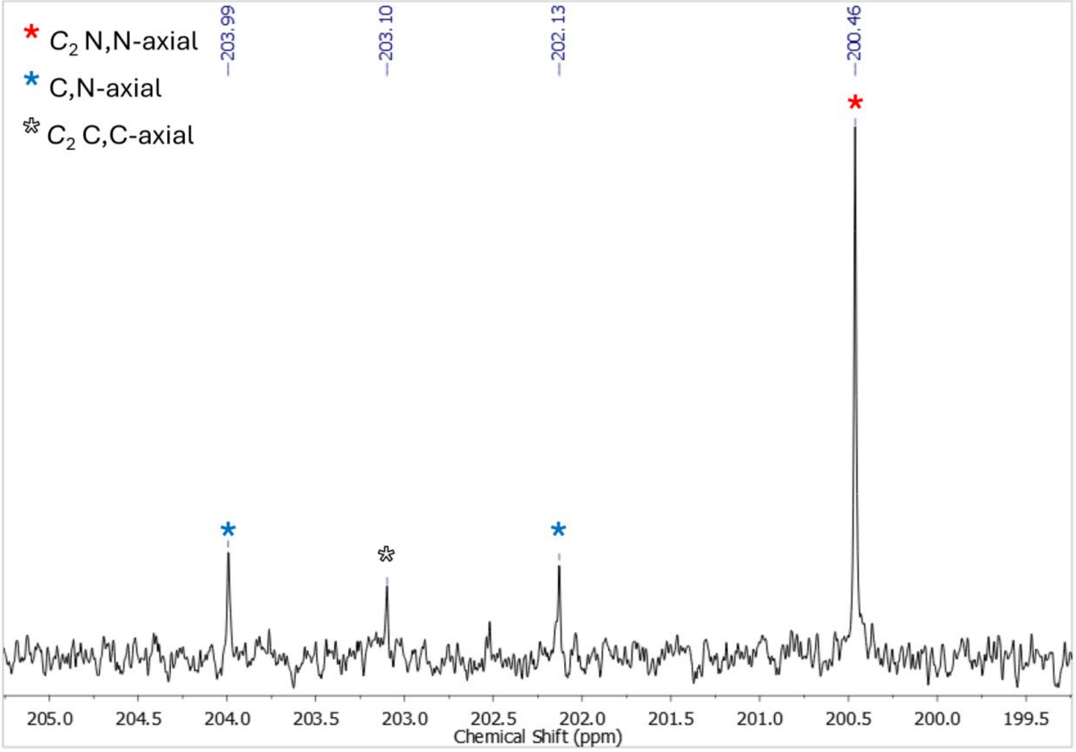

Figure S 7: ^{13}C-NMR of **Fe(Cpy)$_2$(deeb)** (both isomers) in in ACN-d$_3$, carbene resonances.

3.5 Mass Spectrometry

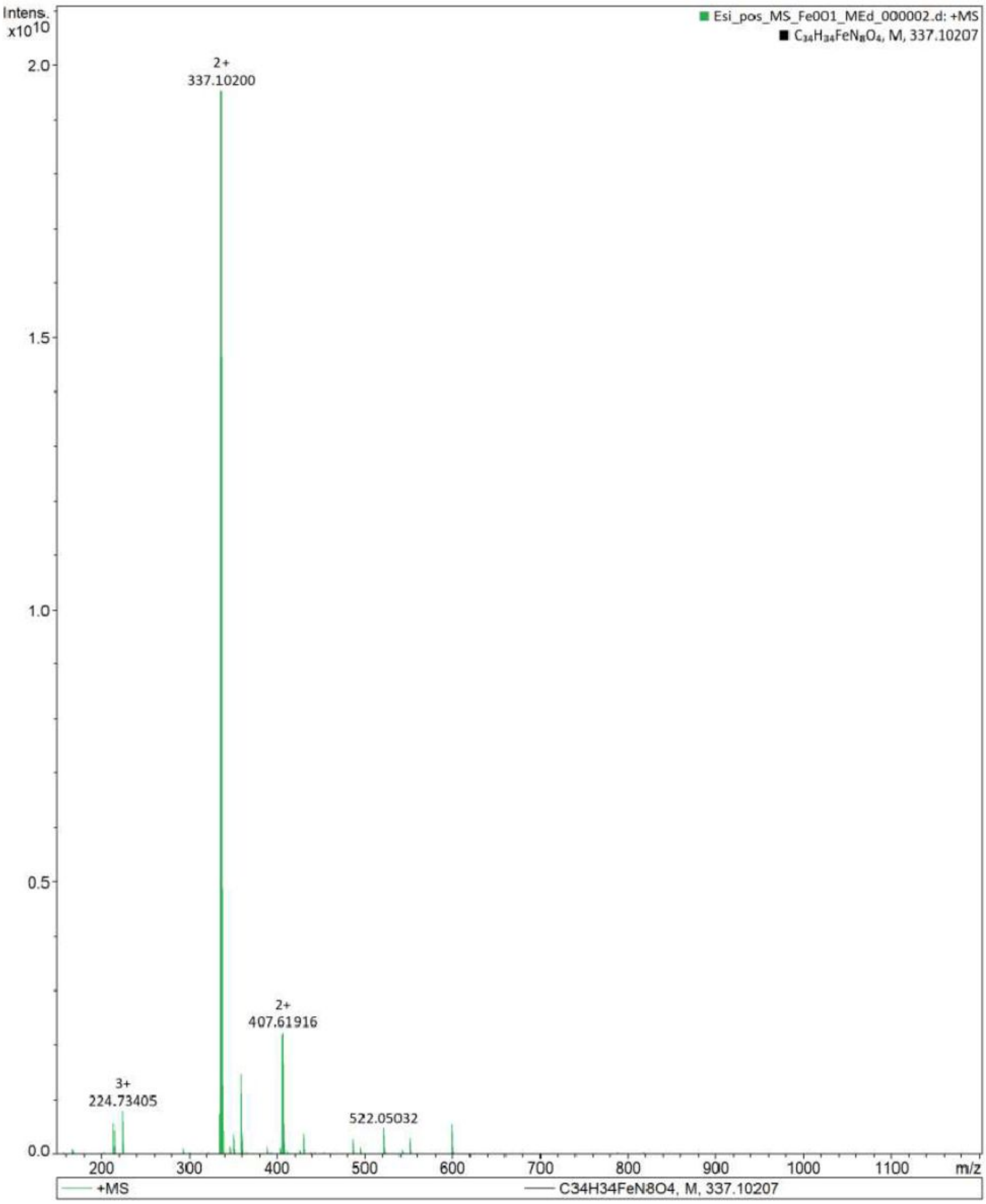

Figure S 8: HR mass spectrum of **$Fe(Cpy)_2(deeb)$**

3.6 Crystallography

Table S 1: Crystal data and structure refinement for CCDC #2503159

Identification code	$Fe(Cpy)_2(deeb)$
Empirical formula	$C_{36}H_{37}F_{12}FeN_9O_4P_2$
Formula weight	1005.53
Temperature/K	150.0
Crystal system	orthorhombic
Space group	*Aea*2
a/Å	15.7974(9)
b/Å	30.4734(16)
c/Å	17.8315(16)
α/°	90
β/°	90
γ/°	90
Volume/Å^3	8584.1(10)
Z	8
ρ_{calc}g/cm^3	1.556
μ/mm^{-1}	0.529
F(000)	4096.0
Crystal size/mm^3	0.07 × 0.04 × 0.03
Radiation	MoKα (λ = 0.71073)
2Θ range for data collection/°	3.694 to 55.22
Index ranges	-20 ≤ h ≤ 20, -39 ≤ k ≤ 39, -23 ≤ l ≤ 23
Reflections collected	95951
Independent reflections	9907 [R_{int} = 0.2344, R_{sigma} = 0.1029]
Data/restraints/parameters	9907/1/582
Goodness-of-fit on F^2	1.052
Final R indexes [I>=2σ (I)]	R_1 = 0.0851, wR_2 = 0.1705
Final R indexes [all data]	R_1 = 0.1190, wR_2 = 0.1874
Largest diff. peak/hole / e Å^{-3}	0.74/-0.48
Flack parameter	0.025(19)

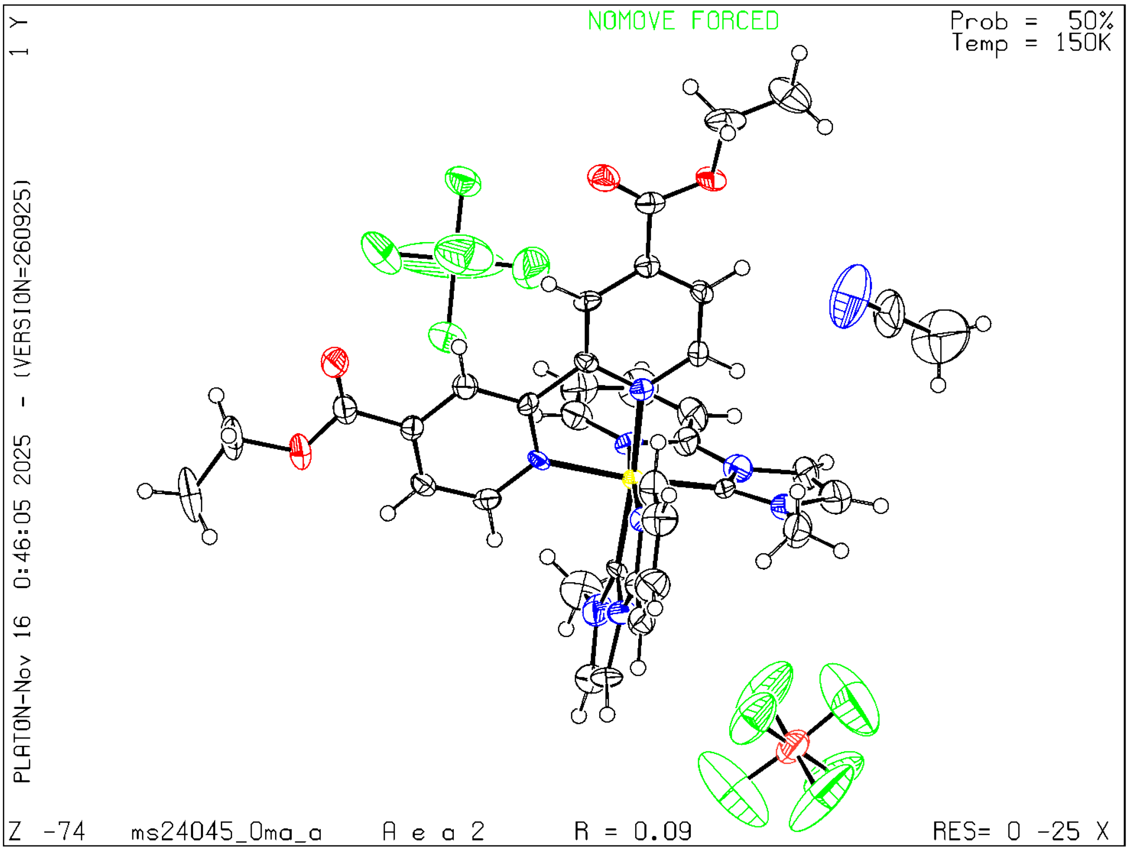

Figure S 9: ORTEP representation of **Fe(Cpy)$_2$(deeb)**

Table S 2: Crystal data and structure refinement for CCDC #2503158

Identification code	**Ru-1**
Empirical formula	$C_{55}H_{70.5}F_{12}N_{6.5}O_{4.5}P_2Ru$
Formula weight	1285.68
Temperature/K	150.0
Crystal system	triclinic
Space group	$P\overline{1}$
a/Å	17.215(5)
b/Å	19.567(5)
c/Å	21.305(7)
α/°	114.807(13)
β/°	104.067(14)
γ/°	100.101(10)
Volume/Å^3	5997(3)
Z	4
ρ_{calc}g/cm^3	1.424
μ/mm^{-1}	0.402
F(000)	2656.0
Crystal size/mm^3	0.05 × 0.05 × 0.05
Radiation	MoKα (λ = 0.71073)
2Θ range for data collection/°	3.472 to 54.926
Index ranges	-22 ≤ h ≤ 22, -25 ≤ k ≤ 25, -27 ≤ l ≤ 27
Reflections collected	136078
Independent reflections	27364 [R_{int} = 0.0547, R_{sigma} = 0.0381]
Data/restraints/parameters	27364/0/1415
Goodness-of-fit on F^2	1.024
Final R indexes [I>=2σ (I)]	R_1 = 0.0557, wR_2 = 0.1428
Final R indexes [all data]	R_1 = 0.0687, wR_2 = 0.1527
Largest diff. peak/hole / e Å^{-3}	1.54/-0.84

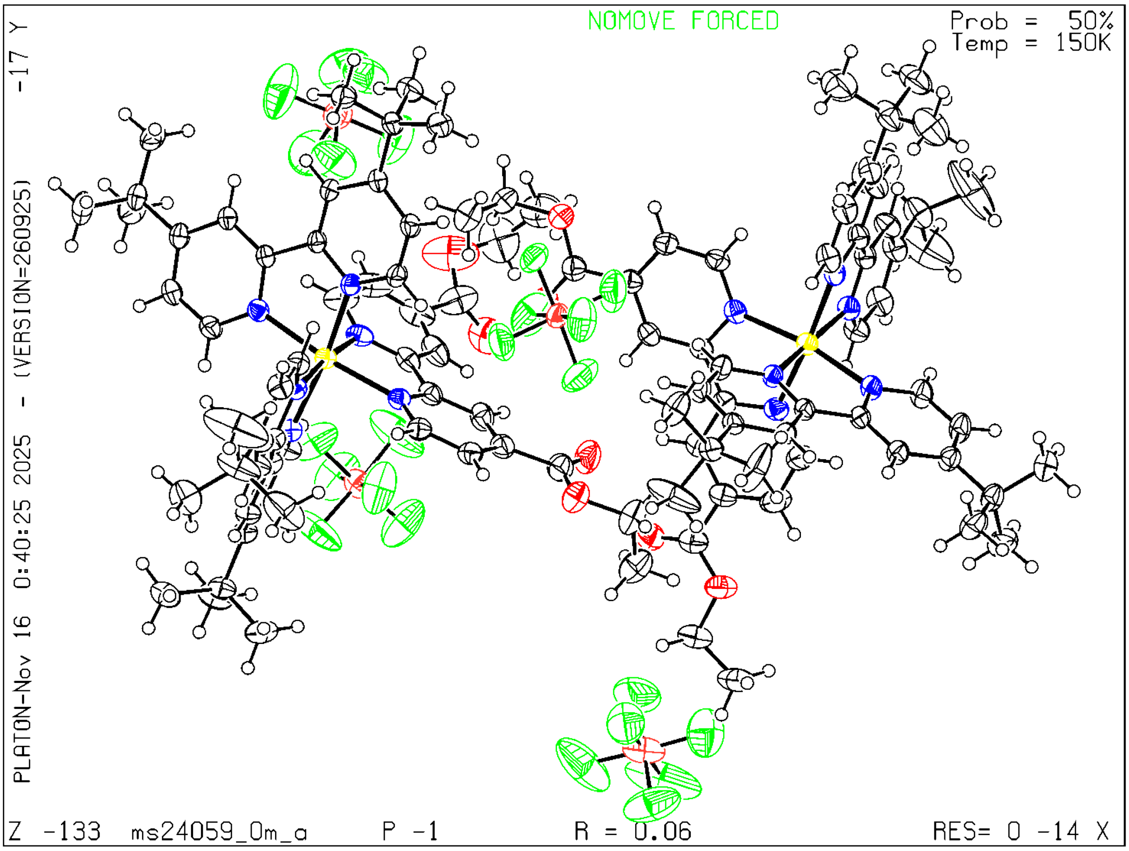

Figure S 10: ORTEP representation of **Ru-1**.

3. Cyclic Voltammetry

Table S 3: Redox processes observed by cyclic voltammetry; concentration of the respective complexes was 1mM in acetonitrile with 0.1M $TBAPF_6$, internal *pseudo* reference Fc/Fc^+

process	4th reduction vs. Fc/Fc^+	3rd reduction vs. Fc/Fc^+	2nd reduction vs. Fc/Fc^+	1st reduction vs. Fc/Fc^+	1st oxidation vs. Fc/Fc^+
**Fe-1**	-/-	-/-	-2.00 V (rev)	**-1.47 V** (rev)	+0.48 V (rev)
**Ru-1**	-2.44 V (rev)	-2.16 V (rev	-1.94 V (rev)	**-1.43 V** (rev)	0.88 V (rev)

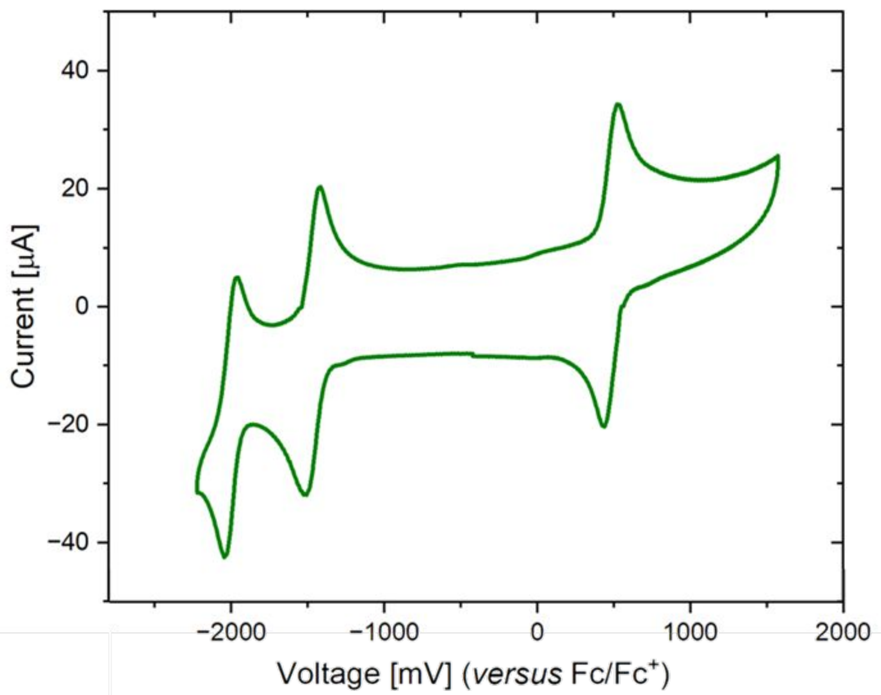

Figure S 11: Cyclic voltammogram of **$Fe(Cpy)_2(deeb)$**, 1mM in acetonitrile with 0.1M $TBAPF_6$.

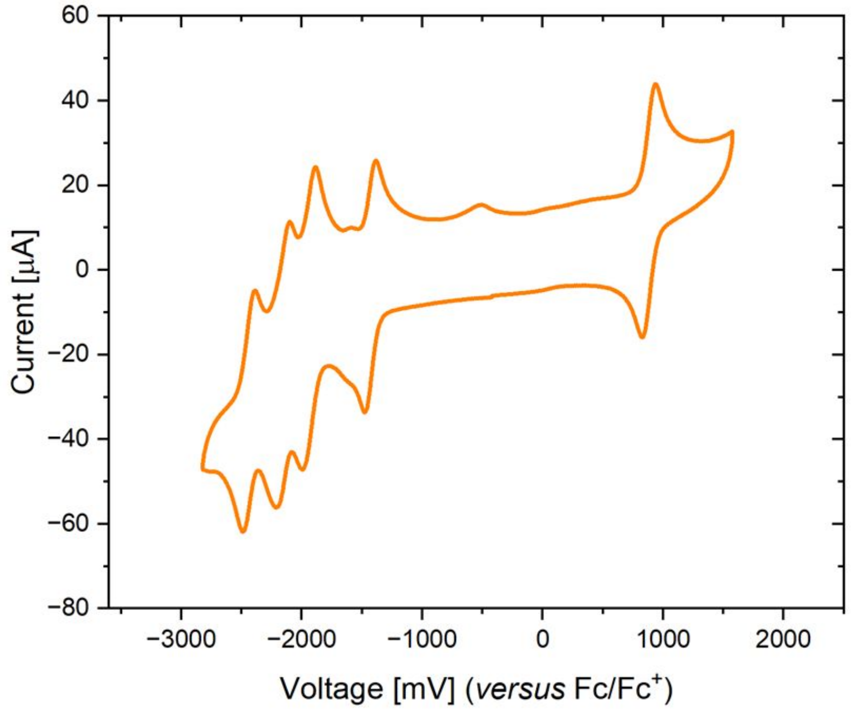

Figure S 12: Cyclic voltammogram of **Ru-1**, 1mM in acetonitrile with 0.1M $TBAPF_6$

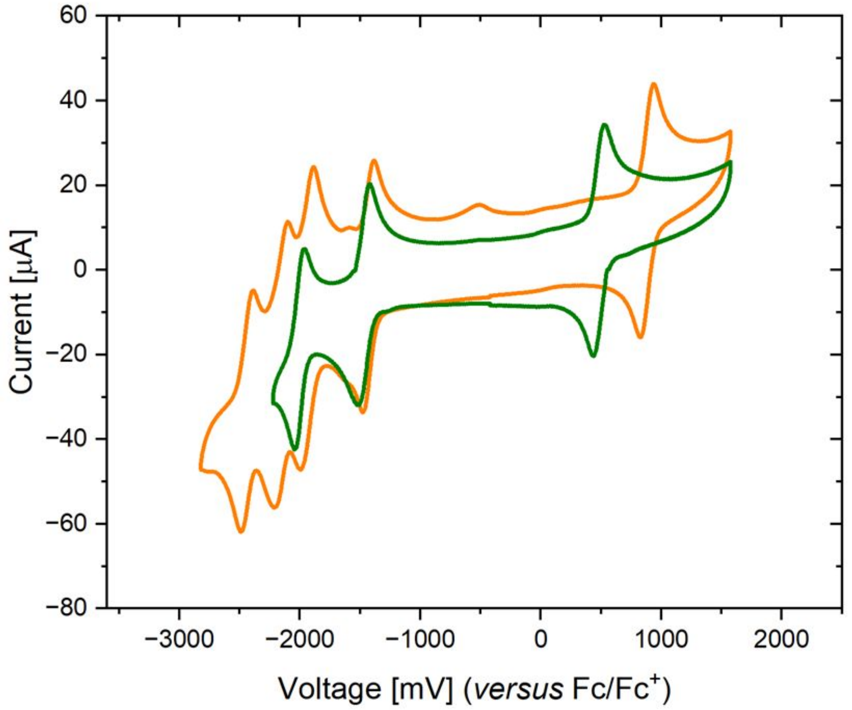

Figure S 13: Cyclic voltammograms of **Fe(Cpy)(deeb)** and **Ru-1** overlayed, both measured as 1mM solutions in acetonitrile with 0.1M $TBAPF_6$.

4. Steady-state UV/Vis Spectroscopy

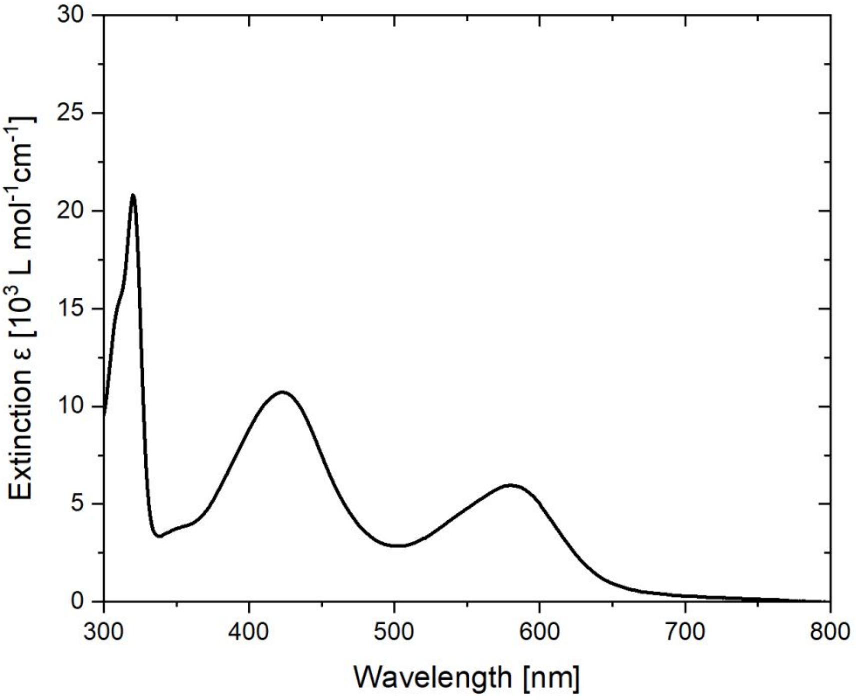

Figure S 14: UV-Vis spectrum of **Fe(Cpy)$_2$(deeb)** in acetonitrile.

5. Computational Results

5.1 B3LYP

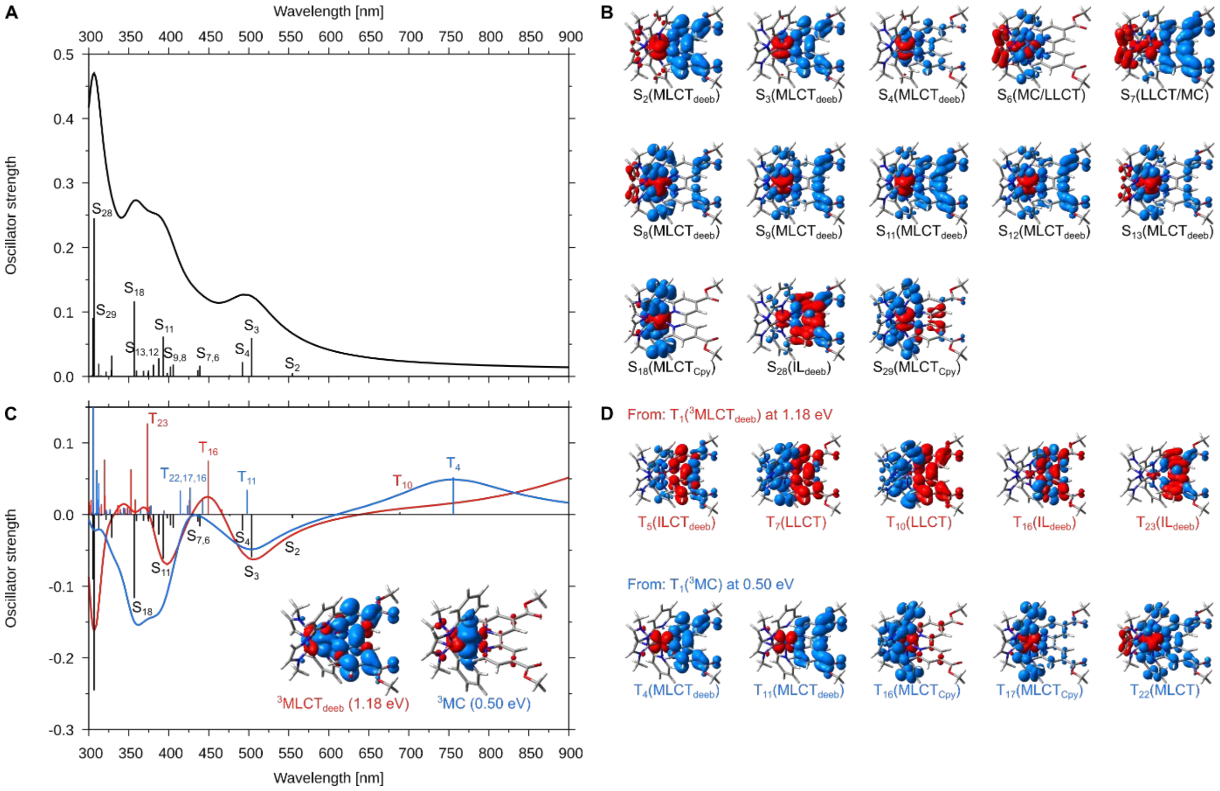

Figure S 15: **A**, simulated electronic absorption spectrum of **Fe(Cpy)$_2$(deeb)** as obtained at the B3LYP/def2-svp level of theory in acetonitrile. Key electronic transitions from the singlet ground state (S_0) are indicated and visualized by charge density difference plots in **B**. Charge transfer occurs from red to blue. **C**, simulated transient absorption spectra as obtained from the fully equilibrated lowest energy $^3MLCT_{deeb}$ state (red) and from the fully relaxed lowest energy ^3MC state (blue), see spin densities and relative energies. Key spin- and dipole-allowed electronic transitions from the respective triplet ground state (T_1) are indicated and visualized by charge density difference plots in **D**.

Table S 4: Calculated electronic absorption spectrum of **Fe(Cpy)$_2$(deeb)** as obtained at the B3LYP/def2-svp level of theory in acetonitrile. Electronic properties of dipole-allowed singlet-singlet (left) and the 20 lowest-energy singlet-triplet transitions (right) are summarized, i.e., electronic characters, excitation energies, excitation wavelengths, oscillator strengths and spin contaminations.

Transition $S_0 \rightarrow S_i$	Character	ΔE_{0i} / eV	λ_i / nm	f	$\langle \hat{S}^2 \rangle$	Transition $S_0 \rightarrow T_i$	Character	ΔE_{0i} / eV	λ_i / nm	$\langle \hat{S}^2 \rangle$
S_2	$MLCT_{deeb}$	2.24	555	0.0050	0.00	T_1	MC	1.53	812	0.00
S_3	$MLCT_{deeb}$	2.46	504	0.0598	0.00	T_2	MC	1.76	704	0.00
S_4	$MLCT_{deeb}$	2.52	492	0.0223	0.00	T_3	$MLCT_{deeb}$	1.91	649	0.00
S_6	MC/LLCT	2.83	439	0.0166	0.00	T_4	$MLCT_{deeb}$	2.00	621	0.00
S_7	LLCT/MC	2.84	436	0.0098	0.00	T_5	MC	2.04	607	0.00
S_8	$MLCT_{deeb}$	3.06	406	0.0186	0.00	T_6	$MLCT_{deeb}$	2.08	596	0.00
S_9	$MLCT_{deeb}$	3.0838	402	0.0153	0.00	T_7	MC/LLCT	2.14	578	0.00
S_{11}	$MLCT_{deeb}$	3.15	393	0.0617	0.00	T_8	MC	2.40	516	0.00
S_{12}	$MLCT_{deeb}$	3.20	388	0.0280	0.00	T_9	MC	2.49	498	0.00
S_{13}	$MLCT_{deeb}$	3.26	381	0.0181	0.00	T_{10}	$MLCT_{deeb}$/LLCT	2.73	454	0.00
S_{18}	$MLCT_{Cpy}$	3.47	357	0.1167	0.00	T_{11}	MLCT	2.81	442	0.00
S_{28}	IL_{deeb}	4.04	307	0.2453	0.00	T_{12}	$MLCT_{deeb}$/LLCT	2.91	426	0.00
S_{29}	$MLCT_{Cpy}$	4.06	305	0.0906	0.00	T_{13}	$MLCT_{deeb}$	2.92	425	0.00
						T_{14}	IL_{deeb}/$MLCT_{deeb}$	3.01	411	0.00
						T_{15}	$MLCT_{deeb}$/LLCT	3.09	401	0.00
						T_{16}	$MLCT_{deeb}$	3.09	401	0.00
						T_{17}	MLCT	3.11	398	0.00
						T_{18}	MLCT	3.15	394	0.00
						T_{19}	$MLCT_{deeb}$	3.27	379	0.00
						T_{20}	$MLCT_{deeb}$	3.28	378	0.00

Table S 5: Electronic characters, as visualized by means of charge density difference plots, of dipole-allowed singlet-singlet transitions and 20 lowest-energy singlet-triplet transitions as obtained at the B3LYP/def2-svp level of theory in acetonitrile. Charge transfer occurs from red to blue.

Singlet-singlet transitions (Franck-Condon point, S_0 structure)			
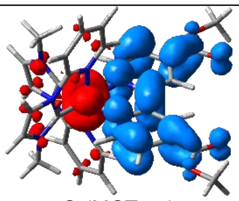	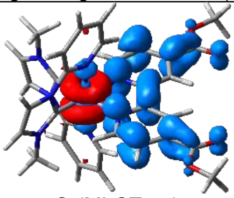	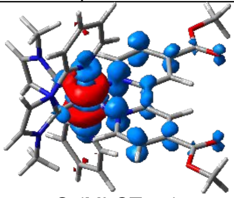	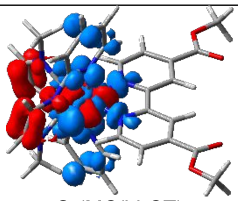
S_2(MCT$_{deeb}$)	S_3(MLCT$_{deeb}$)	S_4(MLCT$_{deeb}$)	S_6(MC/LLCT)
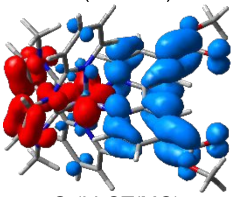	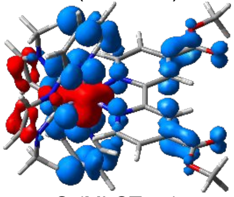	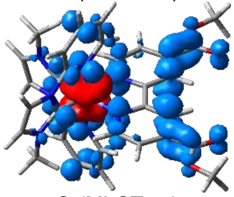	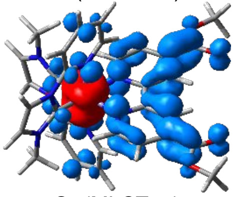
S_7(LLCT/MC)	S_8(MLCT$_{deeb}$)	S_9(MLCT$_{CPY}$)	S_{11}(MLCT$_{Cpy}$)
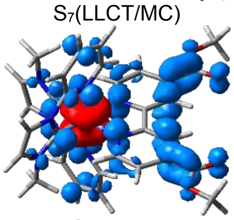	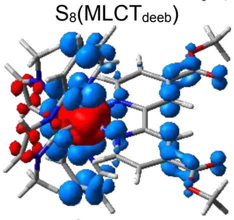	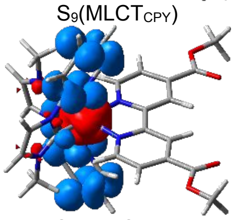	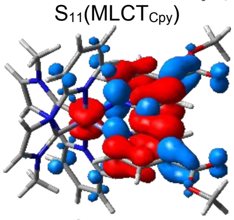
S_{12}(IL$_{deeb}$)	S_{13}(IL$_{deeb}$)	S_{18}(MLCT$_{Cpy}$)	S_{28}(IL$_{deeb}$)
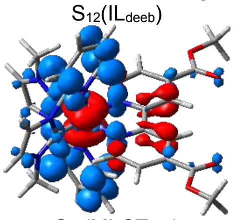			
S_{29}(MLCT$_{Cpy}$)			

Singlet-triplet transitions (Franck-Condon point, S_0 structure)			
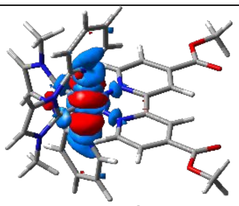	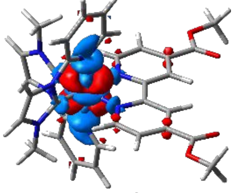	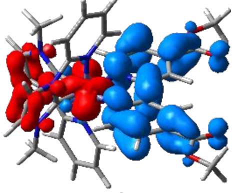	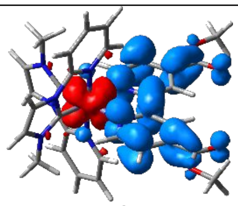
T_1(MC)	T_2(MC)	T_3(MLCT$_{deeb}$)	T_4(MLCT$_{deeb}$)
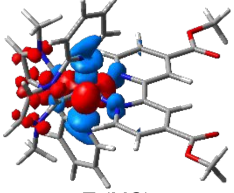	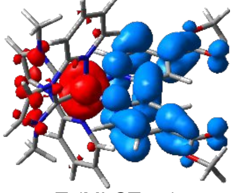	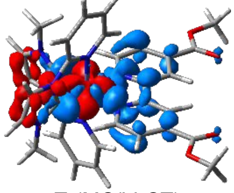	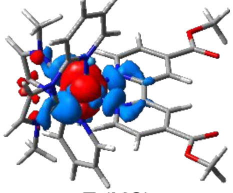
T_5(MC)	T_6(MLCT$_{deeb}$)	T_7(MC/LLCT)	T_8(MC)
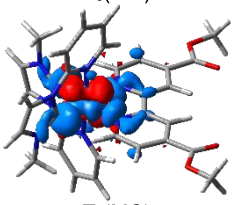	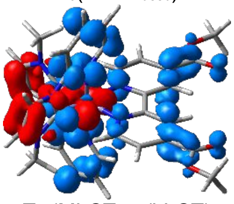	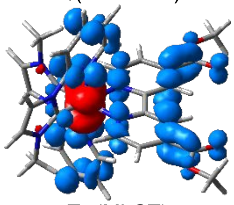	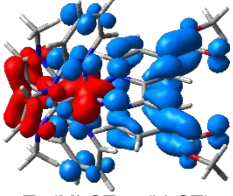
T_9(MC)	T_{10}(MLCT$_{deeb}$/LLCT)	T_{11}(MLCT)	T_{12}(MLCT$_{deeb}$/LLCT)
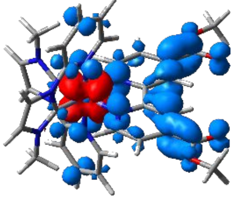	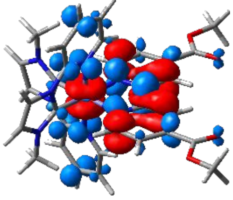	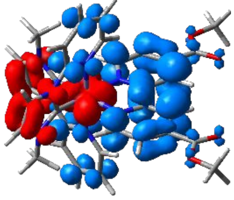	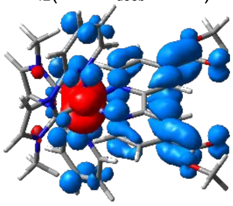

T_{13}(MLCT$_{deeb}$) T_{14}(IL$_{deeb}$/MLCT$_{deeb}$) T_{15}(MLCT$_{deeb}$/LLCT) T_{16}(MLCT$_{deeb}$)

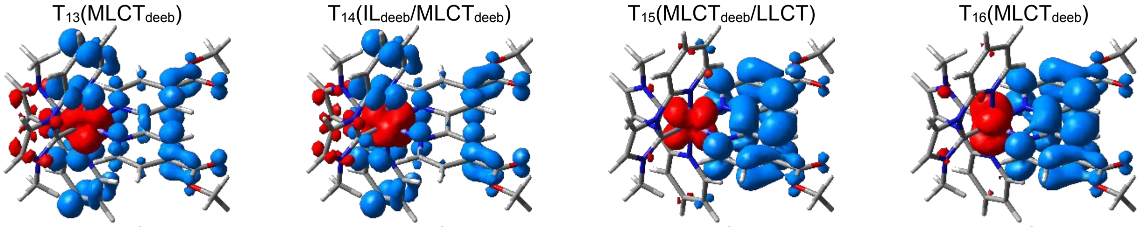

T_{17}(MLCT) T_{18}(MLCT) T_{19}(MLCT$_{deeb}$) T_{20}(MLCT$_{deeb}$)

Table S 6: Calculated spin- and dipole-allowed triplet-triplet transitions contributing to the excited-state absorption of **Fe(Cpy)₂(deeb)** within the fully relaxed ^{3}MLCT structure (left) and within the fully relaxed ^{3}MC (right) structure. All results were obtained at the B3LYP/def2-svp level of theory in acetonitrile.

**^{3}MLCT$_{deeb}$ equilibrium structure (1.18 eV)**						**^{3}MC equilibrium structure (0.50 eV)**					
Transition $T_1 \rightarrow T_i$	Character	ΔE_{1i} / eV	λ_i / nm	f	$\langle \hat{S}^2 \rangle$	Transition $T_1 \rightarrow T_i$	Character	ΔE_{1i} / eV	λ_i / nm	f	$\langle \hat{S}^2 \rangle$
T_5	ILCT$_{deeb}$	1.06	1168	0.0911	2.14	T_4	MLCT$_{deeb}$	1.64	756	0.0521	3.38
T_7	LLCT	1.24	1002	0.0344	2.03	T_{11}	MLCT$_{deeb}$	2.49	498	0.0346	3.13
T_{10}	LLCT	1.80	689	0.0031	2.03	T_{16}	MLCT$_{Cpy}$	2.80	442	0.0201	3.10
T_{16}	IL$_{deeb}$	2.76	449	0.0751	2.15	T_{17}	MLCT$_{Cpy}$	2.91	427	0.0381	2.89
T_{23}	IL$_{deeb}$	3.32	373	0.1272	2.30	T_{22}	MLCT	2.99	414	0.0335	2.86

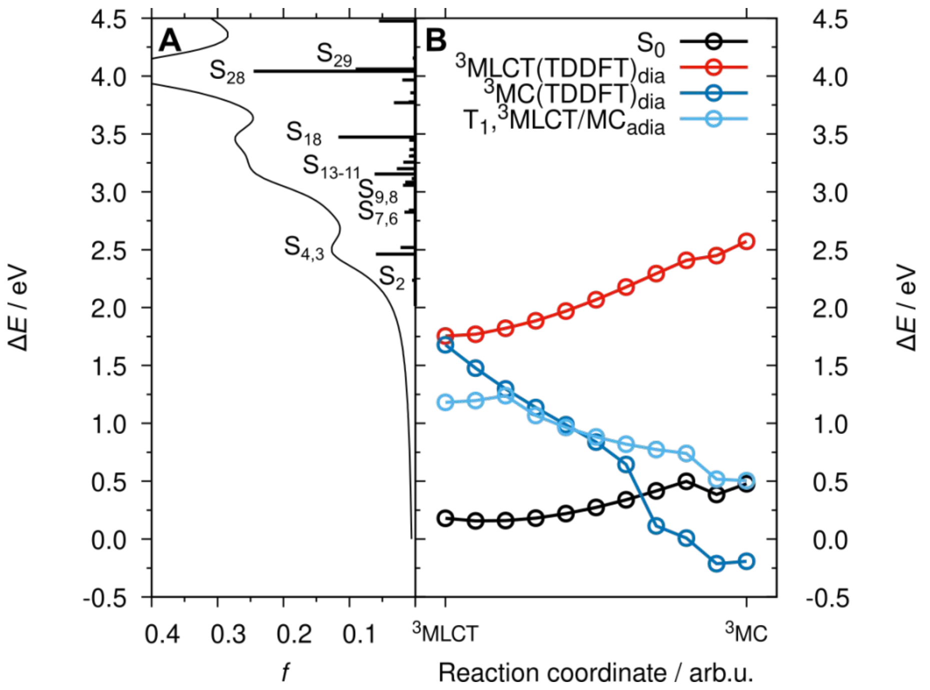

Figure S 16: **A**, simulated electronic absorption spectrum of **Fe(Cpy)$_2$(deeb)** as obtained at the B3LYP/def2-svp level of theory in acetonitrile. Key electronic transitions from the singlet ground state (S_0) are indicated. **B**, potential energy curves (PECs) of the singlet ground state (S_0, black), lowest-energy triplet state (T_1, light blue) as obtained by (restricted and unrestricted) DFT along a linear-interpolated internal coordinate (LIIC), which connects the fully relaxed $^3MLCT_{deeb}$ and ^3MC equilibria. The electronic character of T_1 changes along the LIIC from $^3MLCT_{deeb}$ (left) to ^3MC (right). Further, (diabatic) PECs are shown for the lowest-energy $^3MLCT_{deeb}$ (red) and ^3MC (dark blue) states along the reaction coordinate as obtained at the TDDFT level of theory (singlet-triplet transitions). Notably, TDDFT fails to describe the ^3MC upon partial dissociation (Cpy ligand), see negative energy in the vicinity of the relaxed ^3MC structure.

5.2 B3LYP10

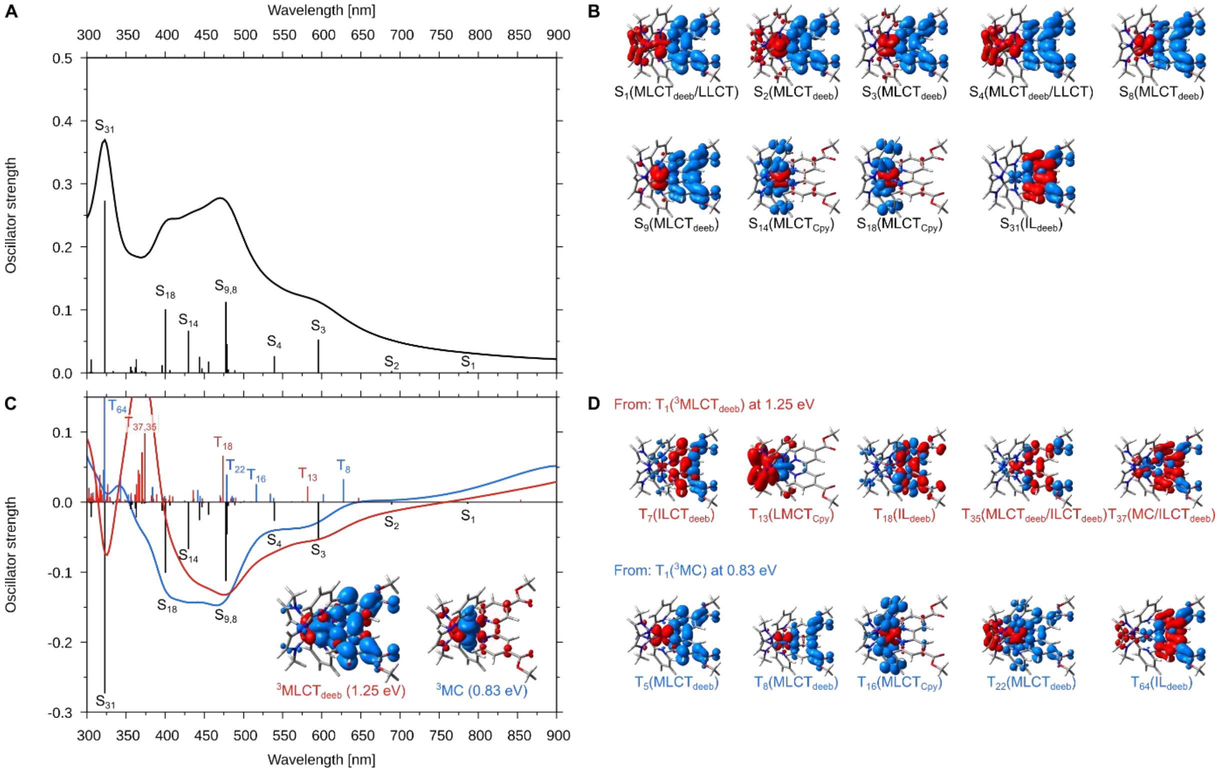

Figure S 17: **A**, simulated electronic absorption spectrum of **Fe(Cpy)$_2$(deeb)** as obtained at the B3LYP10/def2-svp level of theory in acetonitrile. Key electronic transitions from the singlet ground state (S_0) are indicated and visualized by charge density difference plots in **B**. Charge transfer occurs from red to blue. **C**, simulated transient absorption spectra as obtained from the fully equilibrated lowest energy $^3MLCT_{deeb}$ state (red) and from the fully relaxed lowest energy ^3MC state (blue), see spin densities and relative energies. Key spin- and dipole-allowed electronic transitions from the respective triplet ground state (T_1) are indicated and visualized by charge density difference plots in **D**.

Table S 7: Calculated electronic absorption spectrum of **Fe-1** as obtained at the B3LYP10/def2-svp level of theory in acetonitrile. Electronic properties of dipole-allowed singlet-singlet (left) and the 20 lowest-energy singlet-triplet transitions (right) are summarized, i.e., electronic characters, excitation energies, excitation wavelengths, oscillator strengths and spin contaminations.

Transition $S_0 \rightarrow S_i$	Character	ΔE_{0i} / eV	λ_i / nm	f	$\langle \hat{S}^2 \rangle$	Transition $S_0 \rightarrow T_i$	Character	ΔE_{0i} / eV	λ_i / nm	$\langle \hat{S}^2 \rangle$
S_1	$MLCT_{deeb}$/LLCT	1.58	786	0.0024	0.00	T_1	$MLCT_{deeb}$	1.45	855	0.00
S_2	$MLCT_{deeb}$	1.80	689	0.0033	0.00	T_2	$MLCT_{deeb}$	1.54	804	0.00
S_3	$MLCT_{deeb}$	2.08	596	0.0531	0.00	T_3	$MLCT_{deeb}$	1.66	748	0.00
S_4	$MLCT_{deeb}$/LLCT	2.30	539	0.0269	0.00	T_4	MC	1.94	641	0.00
S_8	$MLCT_{deeb}$	2.59	479	0.0461	0.00	T_5	$MLCT_{deeb}$	2.18	568	0.00
S_9	$MLCT_{deeb}$	2.60	478	0.1127	0.00	T_6	MC	2.22	558	0.00
S_{14}	$MLCT_{Cpy}$	2.89	430	0.0671	0.00	T_7	MC/LLCT	2.26	549	0.00
S_{18}	$MLCT_{Cpy}$	3.10	400	0.1011	0.00	T_8	MC	2.30	538	0.00
S_{31}	IL_{deeb}	3.84	323	0.2733	0.00	T_9	LLCT/MC	2.40	517	0.00
						T_{10}	$MLCT_{deeb}$/MC	2.44	509	0.00
						T_{11}	LLCT/MC	2.46	504	0.00
						T_{12}	$MLCT_{deeb}$	2.49	498	0.00
						T_{13}	$MLCT_{Cpy}$	2.55	487	0.00
						T_{14}	$MLCT_{Cpy}$	2.56	485	0.00
						T_{15}	$MLCT_{Cpy}$	2.65	468	0.00
						T_{16}	$MLCT_{deeb}$	2.69	462	0.00
						T_{17}	$MLCT_{Cpy}$	2.70	459	0.00
						T_{18}	$MLCT_{deeb}$	2.72	456	0.00
						T_{19}	$MC/ILCT_{Cpy}$	2.80	442	0.00
						T_{20}	$MC/ILCT_{Cpy}$	3.02	410	0.00

Table S 8: Electronic characters, as visualized by means of charge density difference plots, of dipole-allowed singlet-singlet transitions and 20 lowest-energy singlet-triplet transitions as obtained at the B3LYP10/def2-svp level of theory in acetonitrile. Charge transfer occurs from red to blue.

Singlet-singlet transitions (Franck-Condon point, S_0 structure)

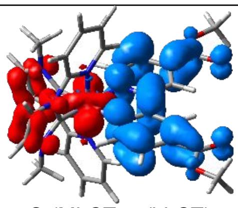

$S_1(MLCT_{deeb}/LLCT)$

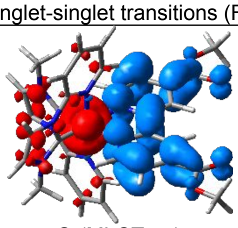

$S_2(MLCT_{deeb})$

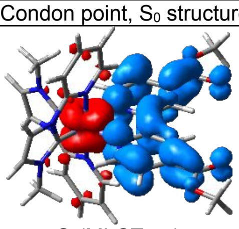

$S_3(MLCT_{deeb})$

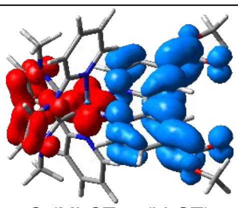

$S_4(MLCT_{deeb}/LLCT)$

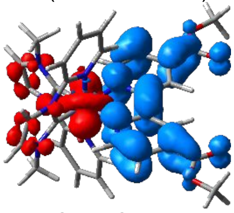

$S_8(MLCT_{deeb})$

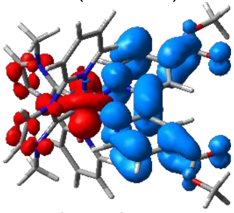

$S_9(MLCT_{deeb})$

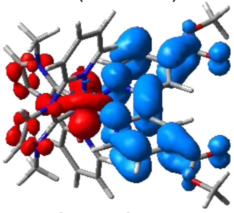

$S_{14}(MLCT_{Cpy})$

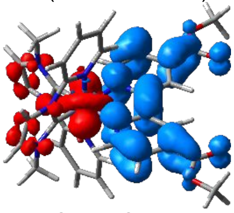

$S_{18}(MLCT_{Cpy})$

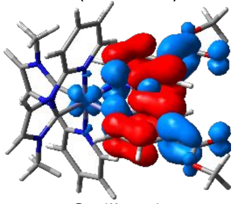

$S_{31}(IL_{deeb})$

Singlet-triplet transitions (Franck-Condon point, S_0 structure)

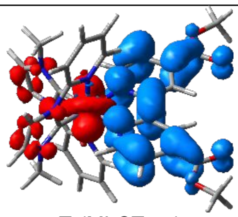

$T_1(MLCT_{deeb})$

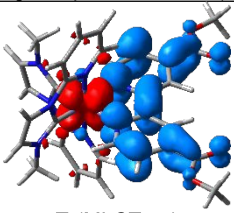

$T_2(MLCT_{deeb})$

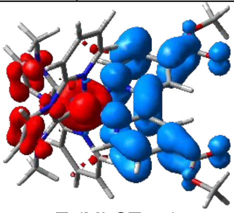

$T_3(MLCT_{deeb})$

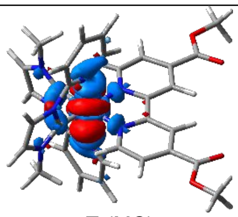

$T_4(MC)$

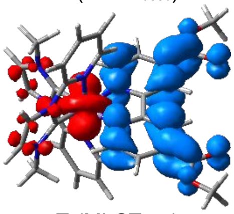

$T_5(MLCT_{deeb})$

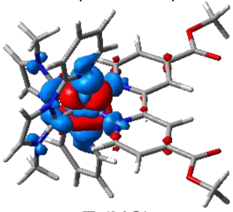

$T_6(MC)$

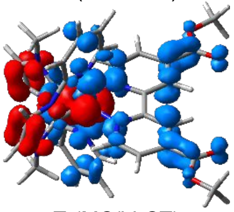

$T_7(MC/LLCT)$

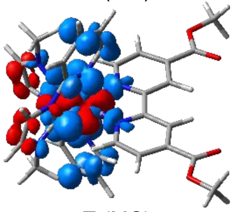

$T_8(MC)$

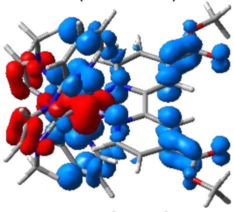

$T_9(LLCT/MC)$

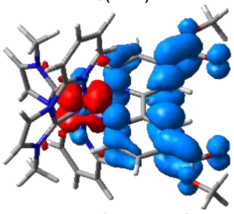

$T_{10}(MLCT_{deeb}/MC)$

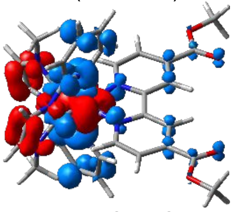

$T_{11}(LLCT/MC)$

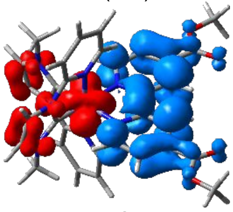

$T_{12}(MLCT_{deeb})$

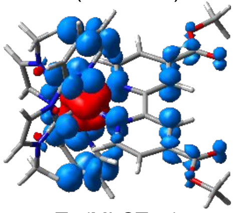

$T_{13}(MLCT_{Cpy})$

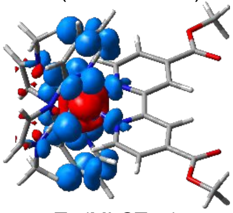

$T_{14}(MLCT_{Cpy})$

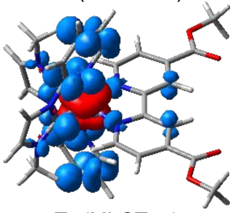

$T_{15}(MLCT_{Cpy})$

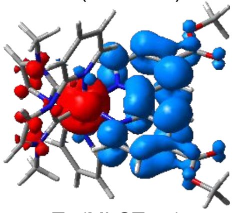

$T_{16}(MLCT_{deeb})$

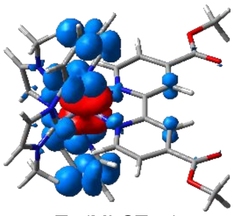
$T_{17}(MLCT_{Cpy})$

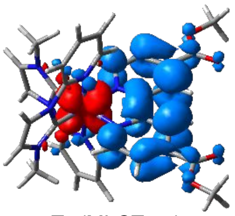
$T_{18}(MLCT_{deeb})$

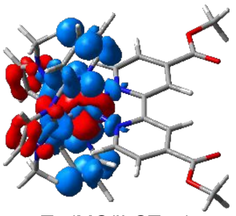
$T_{19}(MC/ILCT_{Cpy})$

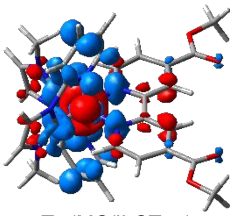
$T_{20}(MC/ILCT_{Cpy})$

Table S 9: Calculated spin- and dipole-allowed triplet-triplet transitions contributing to the excited-state absorption of **Fe(Cpy)$_2$(deeb)** within the fully relaxed ^{3}MLCT structure (left) and within the fully relaxed ^{3}MC (right) structure. All results were obtained at the B3LYP10/def2-svp level of theory in acetonitrile.

**^{3}MLCT$_{deeb}$ equilibrium structure (1.25 eV)**						**^{3}MC equilibrium structure (0.83 eV)**					
Transition $T_1 \rightarrow T_i$	Character	ΔE_{1i} / eV	λ_i / nm	f	$\langle \hat{S}^2 \rangle$	Transition $T_1 \rightarrow T_i$	Character	ΔE_{1i} / eV	λ_i / nm	f	$\langle \hat{S}^2 \rangle$
T_7	ILCT$_{deeb}$	1.08	1148	0.0118	2.02	T_5	MLCT$_{deeb}$	1.35	916	0.0575	3.40
T_{13}	LMCT$_{Cpy}$	2.13	581	0.0220	2.07	T_8	MLCT$_{deeb}$	1.98	628	0.0330	2.83
T_{18}	IL$_{deeb}$	2.62	473	0.0661	2.06	T_{16}	MLCT$_{Cpy}$	2.40	516	0.0258	3.13
T_{35}	MLCT$_{deeb}$/ILCT$_{deeb}$	3.32	374	0.0983	2.28	T_{22}	MLCT$_{deeb}$	2.59	479	0.0395	2.66
T_{37}	MC$_{deeb}$/ILCT$_{deeb}$	3.35	370	0.0709	2.17	T_{64}	IL$_{deeb}$	3.85	322	0.1871	2.31

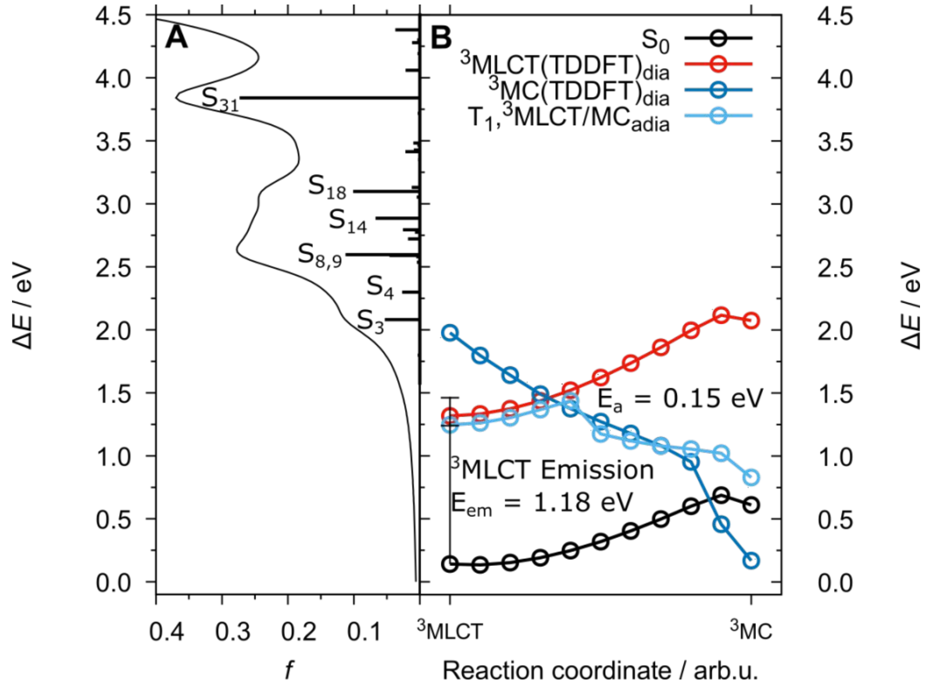

Figure S 18: **A**, simulated electronic absorption spectrum of **Fe(Cpy)$_2$(deeb)** as obtained at the B3LYP10/def2-svp level of theory in acetonitrile. Key electronic transitions from the singlet ground state (S_0) are indicated. **B**, potential energy curves (PECs) of the singlet ground state (S_0, black), lowest-energy triplet state (T_1, light blue) as obtained by (restricted and unrestricted) DFT along a linear-interpolated internal coordinate (LIIC), which connects the fully relaxed $^3MLCT_{deeb}$ and ^3MC equilibria. The electronic character of T_1 changes along the LIIC from $^3MLCT_{deeb}$ (left) to ^3MC (right). Further, (diabatic) PECs are shown for the lowest-energy $^3MLCT_{deeb}$ (red) and ^3MC (dark blue) states along the reaction coordinate as obtained at the TDDFT level of theory (singlet-triplet transitions). Notably, TDDFT fails to describe the ^3MC upon partial dissociation (Cpy ligand), see negative energy in the vicinity of the relaxed ^3MC structure.

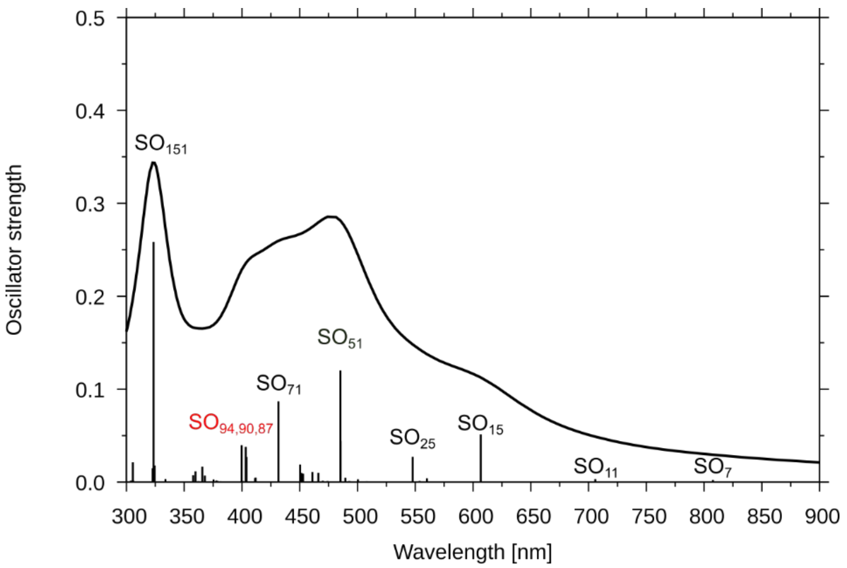

Figure S 19: Simulated electronic absorption spectrum of **Fe(Cpy)$_2$(deeb)** as obtained at the scalar-relativistic B3LYP10/def2-svp level of theory in acetonitrile. Key electronic transitions from the spin-orbit ground state (SO_0) are indicated. Highlighted transitions, mostly of $MLCT_{Cpy}$ character (red), feature sizeable triplet contributions.

Table S 10: Spin-orbit coupling (SOCs in cm^{-1}) matrix elements at the Franck-Condon point between optically accessible singlet excited states (highlighted, bold) and spin forbidden triplet states of **$Fe(Cpy)_2(deeb)$**. Energies (in eV) and oscillator strengths of the respective singlet spin-free transitions (from S_0) are indicated.

			T_1	T_2	T_3	T_4	T_5	T_6	T_7	T_8	T_9	T_{10}	T_{11}	T_{12}	T_{13}	T_{14}	T_{15}	T_{16}	T_{17}	T_{18}	T_{19}	T_{20}	T_{21}	T_{22}	T_{23}	T_{24}	T_{25}
	f	E_{0i} / eV	1.40	1.49	1.62	1.94	2.14	2.23	2.23	2.29	2.36	2.40	2.44	2.44	2.51	2.53	2.61	2.64	2.67	2.68	2.78	3.00	3.02	3.03	3.06	3.07	3.09
S_0	-	0.00	97	29	35	354	121	128	404	258	144	167	52	71	36	114	129	26	37	84	213	6	252	13	64	125	128
S_3	0.053	2.04	151	4	131	49	17	48	66	56	35	84	38	44	2	32	22	10	10	16	21	14	12	11	13	8	32
S_4	0.032	2.26	27	10	44	17	105	97	71	26	45	107	31	3	60	39	16	8	9	14	33	9	15	6	9	7	5
S_8	0.050	2.55	2	2	7	31	146	97	29	39	49	40	19	49	34	33	44	16	20	21	6	5	6	2	4	1	26
S_9	0.123	2.55	22	9	20	47	97	28	41	30	92	117	34	21	4	4	4	29	4	60	25	5	22	5	4	1	4
S_{14}	0.088	2.87	6	79	16	97	13	87	56	59	90	35	0	81	51	7	74	5	23	15	39	14	3	12	7	8	66
S_{18}	0.094	3.08	25	30	3	45	27	6	53	17	61	6	6	37	24	15	35	1	11	15	10	32	102	12	6	16	119

5.3 TPSSh

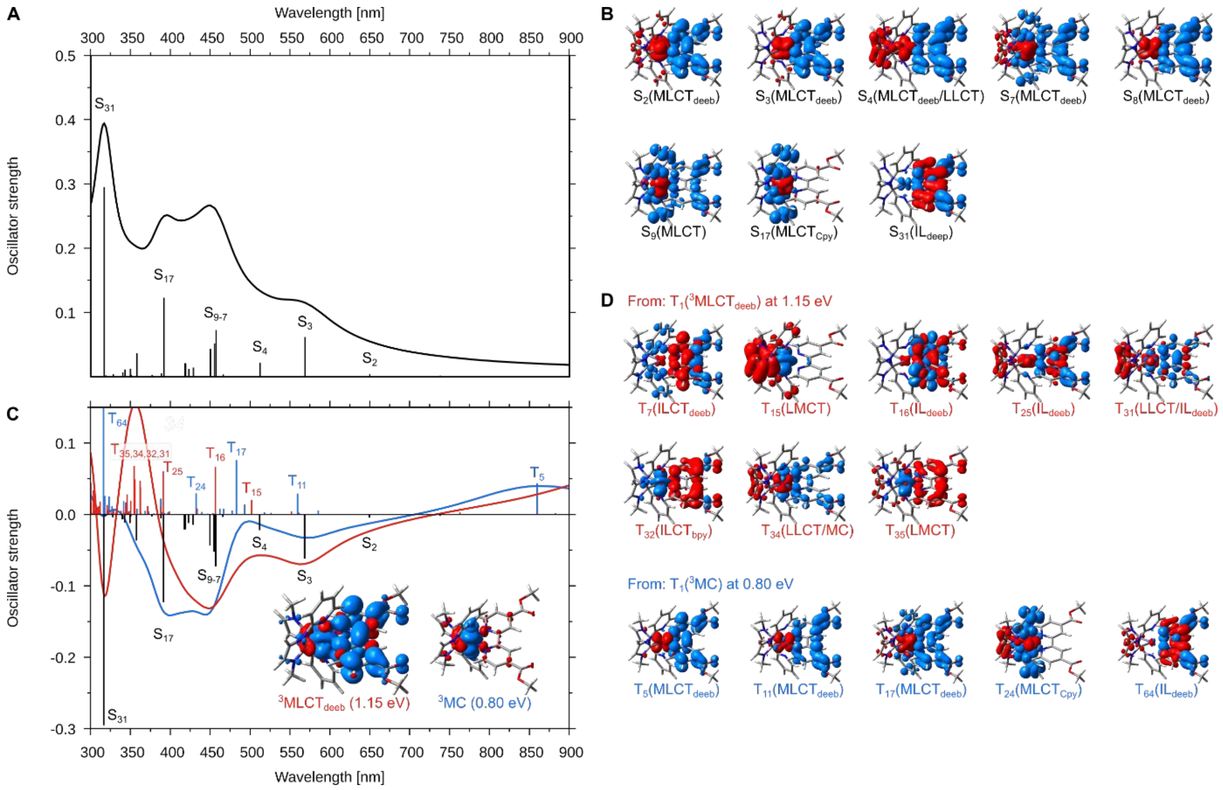

Figure S 20: **A**, simulated electronic absorption spectrum of **Fe(Cpy)$_2$(deeb)** as obtained at the TPSSh/def2-svp level of theory in acetonitrile. Key electronic transitions from the singlet ground state (S_0) are indicated and visualized by charge density difference plots in **B**. Charge transfer occurs from red to blue. **C**, simulated transient absorption spectra as obtained from the fully equilibrated lowest energy $^3MLCT_{deeb}$ state (red) and from the fully relaxed lowest energy ^3MC state (blue), see spin densities and relative energies. Key spin- and dipole-allowed electronic transitions from the respective triplet ground state (T_1) are indicated and visualized by charge density difference plots in **D**.

Table S 11: Calculated electronic absorption spectrum of **Fe(Cpy)$_2$(deeb)** as obtained at the TPSSh/def2-svp level of theory in acetonitrile. Electronic properties of dipole-allowed singlet-singlet (left) and the 20 lowest-energy singlet-triplet transitions (right) are summarized, i.e., electronic characters, excitation energies, excitation wavelengths, oscillator strengths and spin contaminations.

Transition $S_0 \rightarrow S_i$	Character	ΔE_{0i} / eV	λ_i / nm	f	$\langle \hat{S}^2 \rangle$	Transition $S_0 \rightarrow T_i$	Character	ΔE_{0i} / eV	λ_i / nm	$\langle \hat{S}^2 \rangle$
S_2	$MLCT_{deeb}$	1.91	650	0.0044	0.00	T_1	$MLCT_{deeb}$	1.54	804	0.00
S_3	$MLCT_{deeb}$	2.19	568	0.0619	0.00	T_2	$MLCT_{deeb}$/MC	1.60	775	0.00
S_4	$MLCT_{deeb}$/LLCT	2.42	512	0.0219	0.00	T_3	MC	1.73	718	0.00
S_7	$MLCT_{deeb}$	2.71	457	0.0727	0.00	T_4	$MLCT_{deeb}$	1.74	713	0.00
S_8	$MLCT_{deeb}$	2.73	455	0.0520	0.00	T_5	MC	1.99	622	0.00
S_9	MLCT	2.76	450	0.0432	0.00	T_6	MC	2.20	563	0.00
S_{17}	$MLCT_{Cpy}$	3.17	392	0.1230	0.00	T_7	MC	2.22	558	0.00
S_{31}	IL_{deeb}	3.91	317	0.2952	0.00	T_8	$MLCT_{deeb}$/LLCT	2.32	534	0.00
						T_9	$MLCT_{deeb}$	2.45	505	0.00
						T_{10}	MLCT/MC	2.53	491	0.00
						T_{11}	MLCT/MC	2.54	489	0.00
						T_{12}	$MLCT_{deeb}$	2.55	485	0.00
						T_{13}	MLCT	2.60	476	0.00
						T_{14}	$MLCT_{deeb}$/LLCT	2.66	465	0.00
						T_{15}	$MLCT_{Cpy}$	2.76	449	0.00
						T_{16}	$MLCT_{Cpy}$	2.77	447	0.00
						T_{17}	$MLCT_{Cpy}$	2.84	436	0.00
						T_{18}	$MLCT_{deeb}$	2.85	435	0.00
						T_{19}	$MLCT_{deeb}$	2.88	430	0.00
						T_{20}	IL_{deeb}	2.97	418	0.00

Table S 12: Electronic characters, as visualized by means of charge density difference plots, of dipole-allowed singlet-singlet transitions and 20 lowest-energy singlet-triplet transitions as obtained at the TPSSh/def2-svp level of theory in acetonitrile. Charge transfer occurs from red to blue.

Singlet-singlet transitions (Franck-Condon point, S_0 structure)			
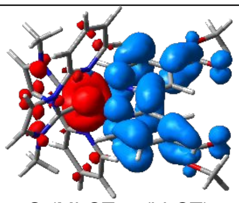 S_2(MLCT$_{deeb}$/LLCT)	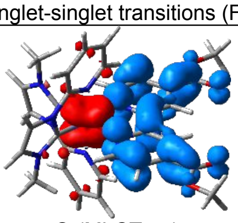 S_3(MLCT$_{deeb}$)	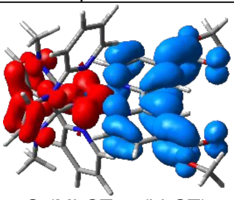 S_4(MLCT$_{deeb}$/LLCT)	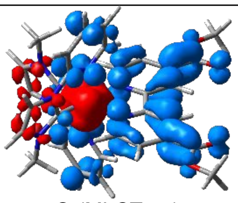 S_7(MLCT$_{deeb}$)
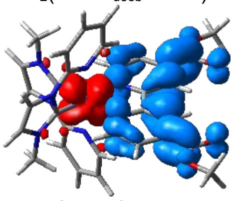 S_8(MLCT$_{deeb}$)	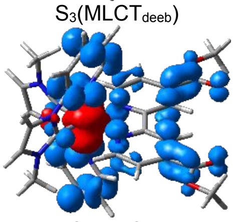 S_9(MLCT)	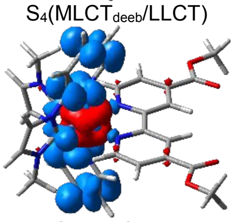 S_{17}(MLCT$_{Cpy}$)	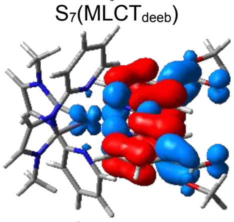 S_{31}(IL$_{deeb}$)
**Singlet-triplet transitions (Franck-Condon point, S_0 structure)**			
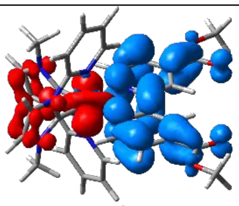 T_1(MLCT$_{deeb}$)	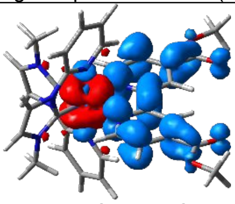 T_2(MLCT$_{deeb}$/MC)	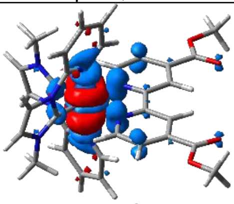 T_3(MC)	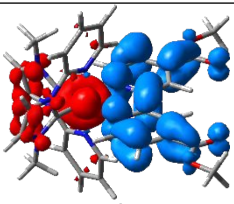 T_4(MLCT$_{deeb}$)
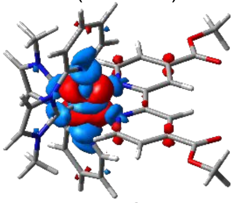 T_5(MC)	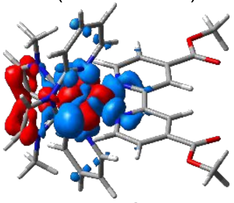 T_6(MC)	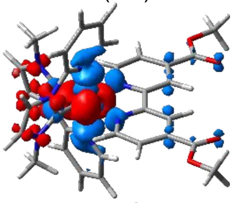 T_7(MC)	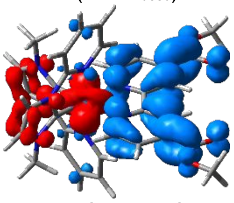 T_8(MLCT$_{deeb}$/LLCT)
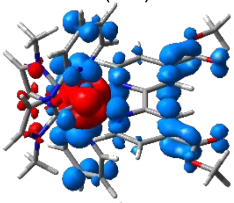 T_9(MLCT$_{deeb}$)	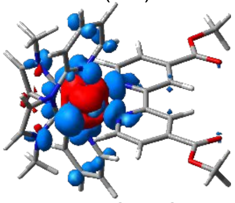 T_{10}(MLCT/MC)	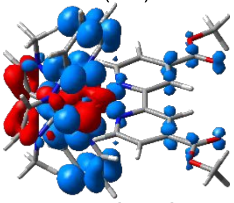 T_{11}(MLCT/MC)	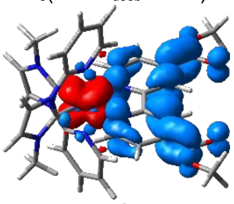 T_{12}(MLCT$_{deeb}$)
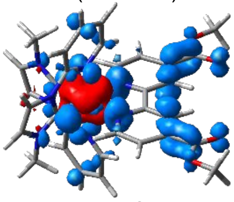 T_{13}(MLCT)	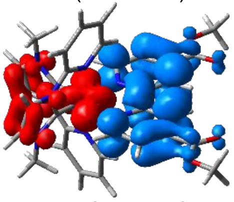 T_{14}(MLCT$_{deeb}$/LLCT)	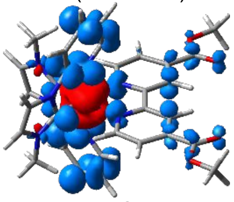 T_{15}(MLCT$_{Cpy}$)	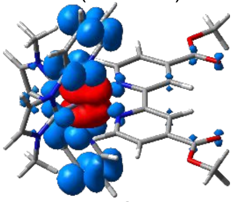 T_{16}(MLCT$_{Cpy}$)
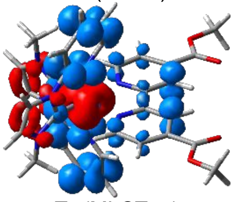 T_{17}(MLCT$_{Cpy}$)	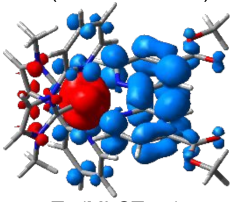 T_{18}(MLCT$_{deeb}$)	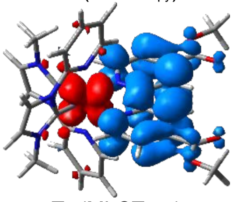 T_{19}(MLCT$_{deeb}$)	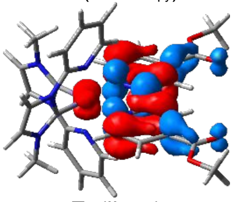 T_{20}(IL$_{deeb}$)

Table S 13: Calculated spin- and dipole-allowed triplet-triplet transitions contributing to the excited-state absorption of **Fe(Cpy)$_2$(deeb)** within the fully relaxed $^3MLCT_{deeb}$ structure (left) and within the fully relaxed ^3MC (right) structure. All results were obtained at the TPSSh/def2-svp level of theory in acetonitrile.

**$^3MLCT_{deeb}$ equilibrium structure (1.15 eV)**						**^3MC equilibrium structure (0.80 eV)**					
Transition $T_1 \rightarrow T_i$	Character	ΔE_{1i} / eV	λ_i / nm	f	$\langle \hat{S}^2 \rangle$	Transition $T_1 \rightarrow T_i$	Character	ΔE_{1i} / eV	λ_i / nm	f	$\langle \hat{S}^2 \rangle$
T_7	$ILCT_{deeb}$	1.14	1089	0.0915	2.04	T_5	$MLCT_{deeb}$	1.44	860	0.0438	3.54
T_{15}	LMCT	2.47	502	0.0204	2.11	T_{11}	$MLCT_{deeb}$	2.22	560	0.0291	3.20
T_{16}	IL_{deeb}	2.72	457	0.0665	2.15	T_{17}	$MLCT_{deeb}$	2.57	483	0.0761	2.66
T_{25}	IL_{deeb}	3.17	391	0.0603	2.30	T_{24}	$MLCT_{Cpy}$	2.87	432	0.0297	2.91
T_{31}	$LLCT/IL_{deeb}$	3.42	362	0.0380	2.44	T_{64}	IL_{deeb}	3.92	316	0.2161	2.28
T_{32}	$ILCT_{deeb}$	3.42	362	0.0472	2.29						
T_{34}	LMCT	3.49	356	0.0491	2.29						
T_{35}	LLCT	3.50	355	0.0679	2.14						

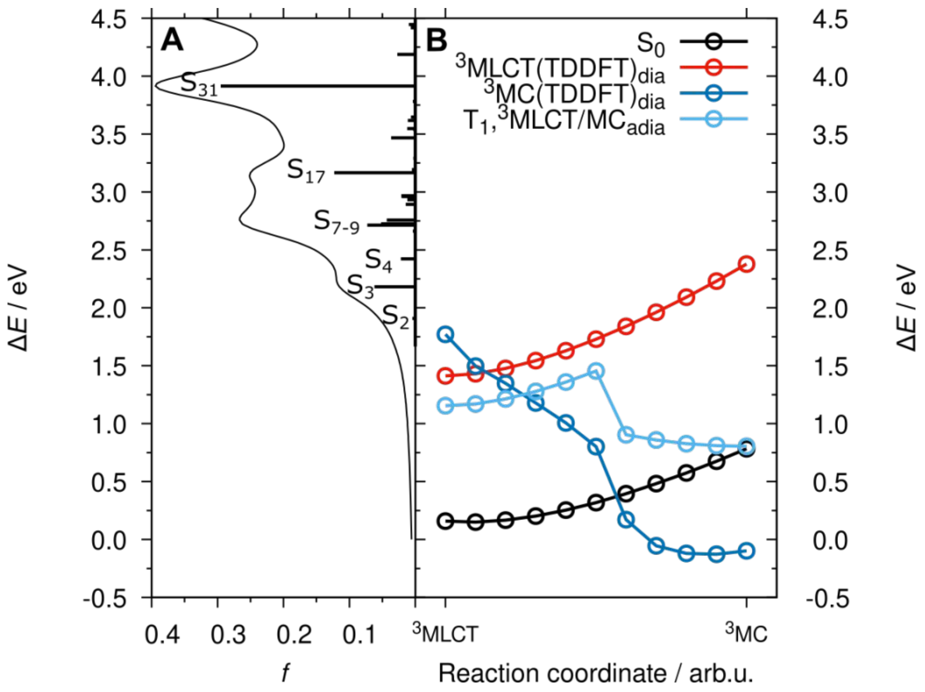

Figure S 21: **A**, simulated electronic absorption spectrum of **Fe(Cpy)$_2$(deeb)** as obtained at the TPSSh/def2-svp level of theory in acetonitrile. Key electronic transitions from the singlet ground state (S_0) are indicated. **B**, potential energy curves (PECs) of the singlet ground state (S_0, black), lowest-energy triplet state (T_1, light blue) as obtained by (restricted and unrestricted) DFT along a linear-interpolated internal coordinate (LIIC), which connects the fully relaxed $^3MLCT_{deeb}$ and ^3MC equilibria. The electronic character of T_1 changes along the LIIC from $^3MLCT_{deeb}$ (left) to ^3MC (right). Further, (diabatic) PECs are shown for the lowest-energy $^3MLCT_{deeb}$ (red) and ^3MC (dark blue) states along the reaction coordinate as obtained at the TDDFT level of theory (singlet-triplet transitions). Notably, TDDFT fails to describe the ^3MC upon partial dissociation (CPY ligand), see negative energy in the vicinity of the relaxed ^3MC structure.

6. Raman Spectroscopy

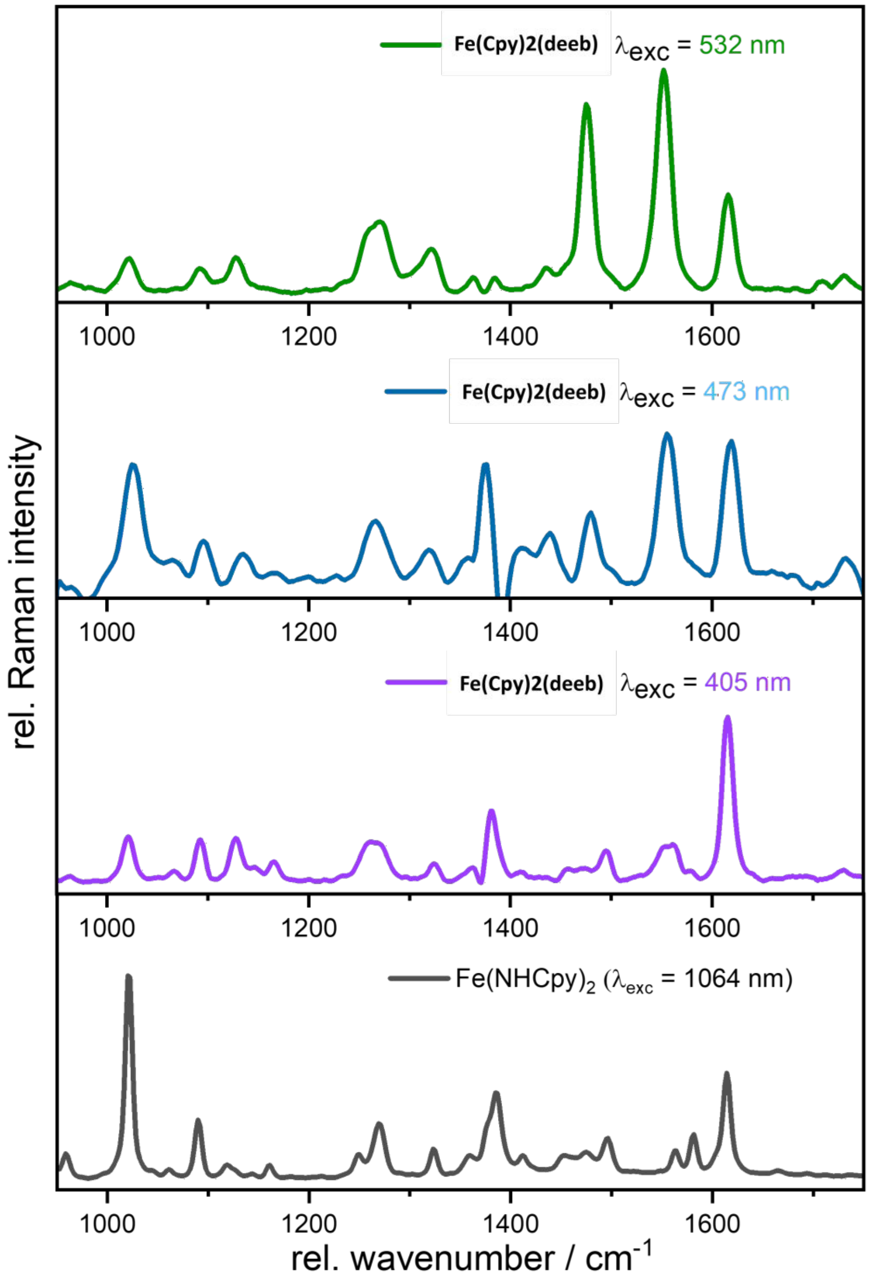

Figure S 22: A-C: Resonance Raman spectra of the complex **Fe(cpy)$_2$(deeb)** in acetonitrile at the excitation wavelengths at 532 (A), 473 (B) and 405 nm (C). D: Non-resonant Raman spectrum of the solid homoleptic $[Fe(NHCpy)_2(NCMe)_2]^{2+}$ complex which serves as reference compound for band assignment.

7. TAS Excited-State Lifetime Kinetic Fits

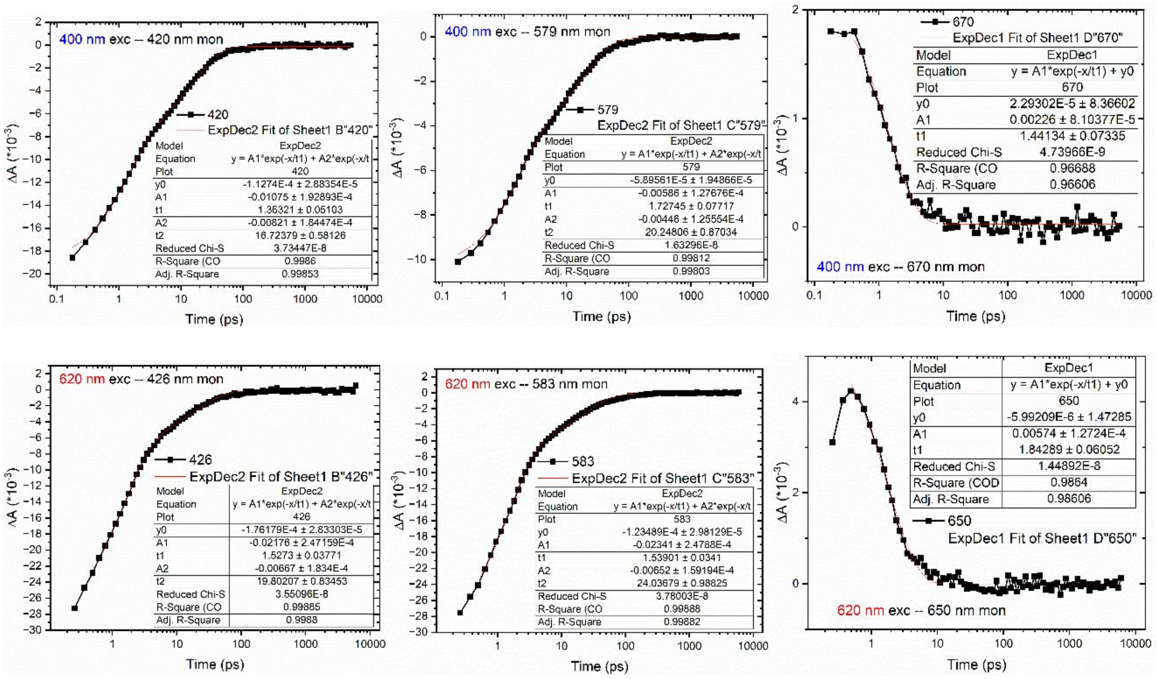

Figure S 23: Ultrafast kinetic data for **Fe(Cpy)₂(deeb)** in acetonitrile collected using either 400 nm (top) or 620 nm (bottom) excitation. The kinetics monitored at 420/426 nm and 579/583 nm were fit to a biexponential model, those monitored at 650/670 nm were fit to a single exponential model. To one significant figure, the kinetic data is well described by a 2 ps component and a 20 ps component.

8. Solution Spectroelectrochemistry

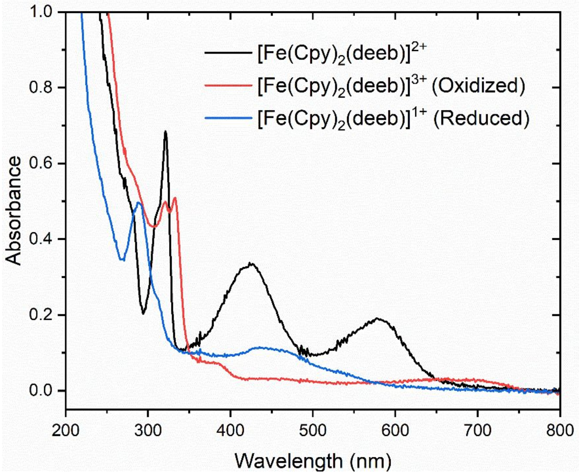

Figure S 24: UV-Vis spectrum of **$Fe(Cpy)_2(deeb)$** in 0.1 M $TBAPF_6$ acetonitrile (black) plotted against the singly oxidized (red) and singly reduced (blue) forms.

9 Deprotection of deeb

9.1 Method 1

A 0.10 mL aliquot of an acetonitrile solution of $Fe(Cpy)_2(deeb)$ (~1 mM) was transferred to a 10 mL microwave reactor tube equipped with a stir bar. To the microwave tube was added

0.30 mL of deionized water, 0.30 mL of 200 proof ethanol, and 18 ml of a 0.10 M aqueous solution of K_2CO_3. The faint green solution was microwaved at 80 degrees Celsius for 30 minutes resulting in a faint red solution. The solution was neutralized with dropwise addition of 0.1 M HCl until the pH reached 5, resulting in a greenish solution. The solvent was then stripped under reduced pressure and redissolved in minimal acetonitrile for use in dye loading.

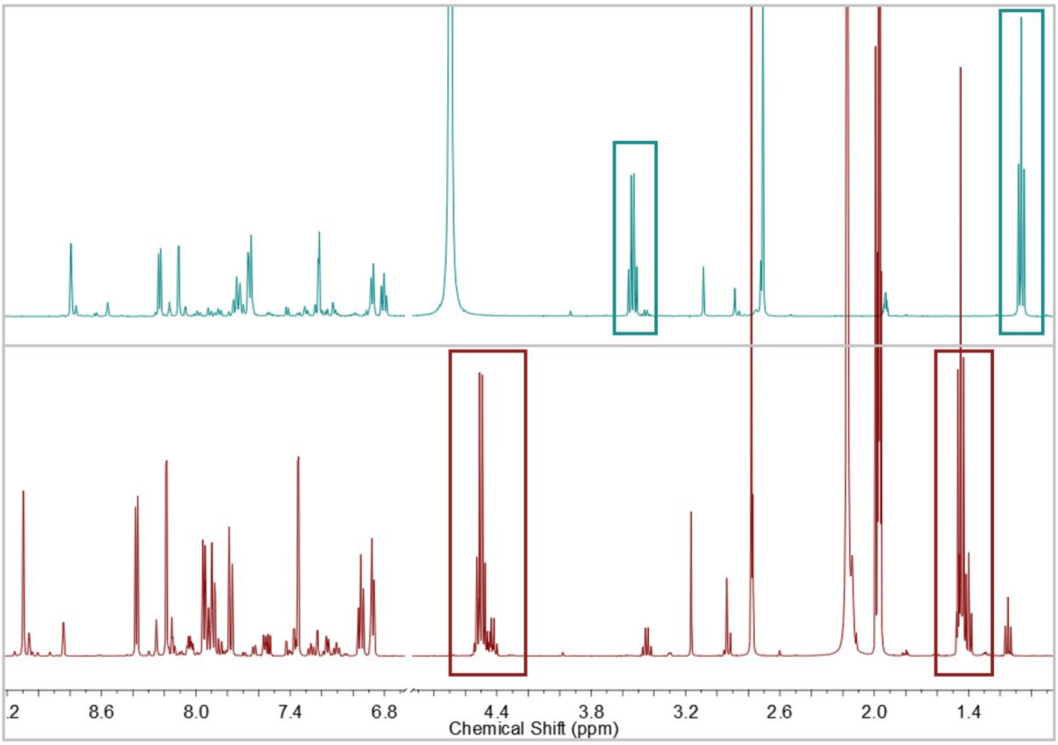

Figure S 25: ^{1}H-NMR spectrum of **Fe(Cpy)$_2$(deeb)** (bottom) and the deprotected analogue, **Fe(Cpy)$_2$(dcb)** (top). 400 MHz in CD_3CN; frames highlight ethyl functions before deprotection (bottom), and free ethanol (top) after deprotection.

9.2 Method 2

A small portion of solid **Fe(Cpy)$_2$(deeb)** was added to a vial with ~3 mL H_2O. The solid was weakly soluble and resulted in a faint green solution with solid floating freely therein. The solution was then heated overnight at 60 degrees Celsius resulting in a homogeneous, reddish solution.

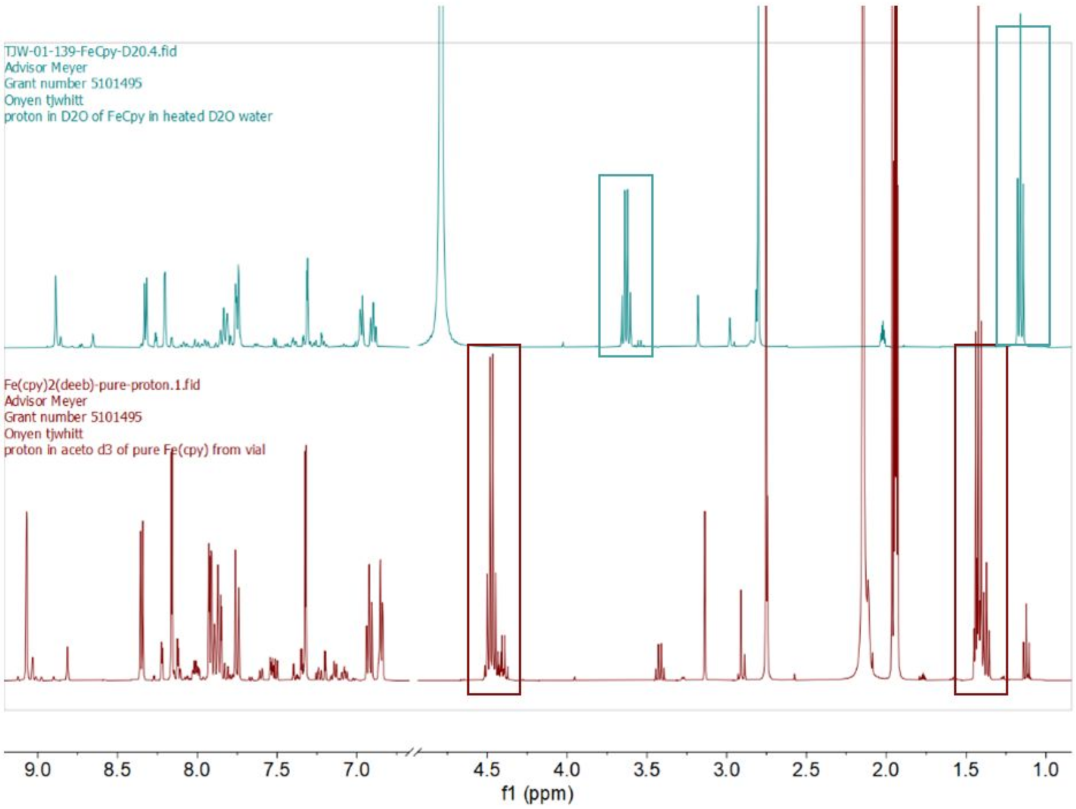

Figure S 26: ^{1}H-NMR spectrum of **Fe(Cpy)$_2$(deeb)** (bottom, CD_3CN) and the deprotected analogue, **Fe(Cpy)$_2$(dcb)** (top, D_2O). 400 MHz; frames highlight ethyl functions before deprotection (bottom), and free ethanol (top) after deprotection.

10. Acid/Base Absorbance Spectrum

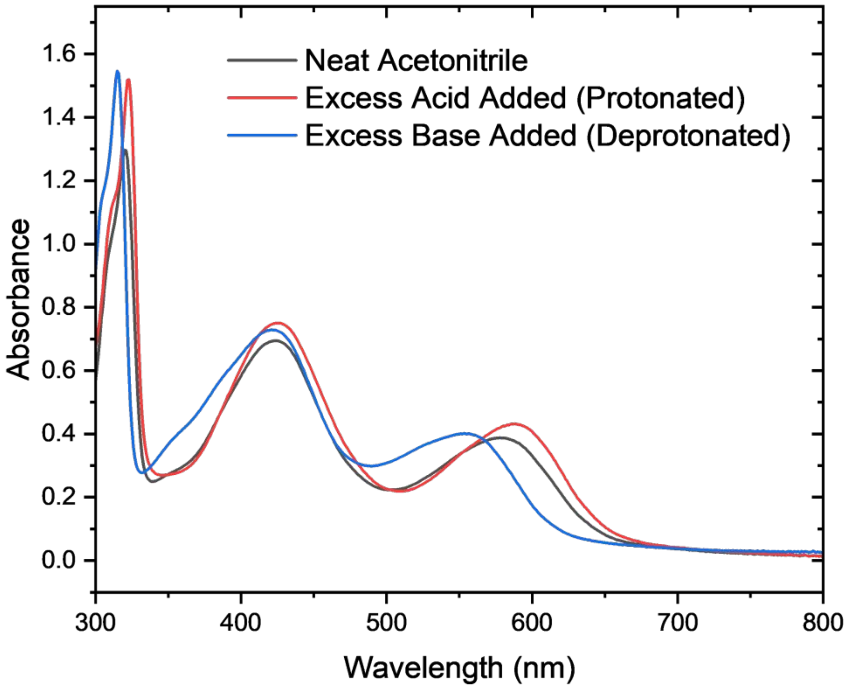

Figure S 27: UV-Vis spectrum of **Fe(Cpy)$_2$(dcb)** in neat acetonitrile (black), and with added 1 M aq. HCl (red) and added 1% NaOH aq. (blue).

11. ITO Electrochemistry

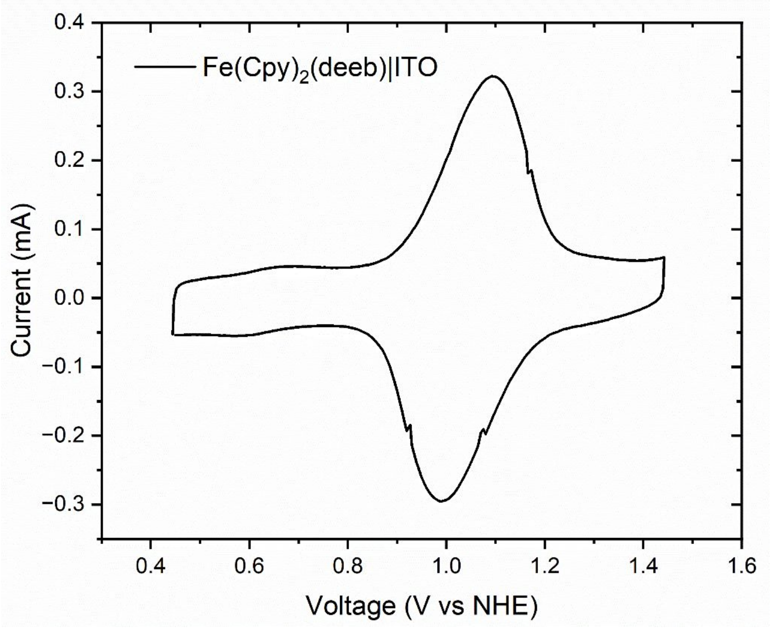

Figure S 28: Cyclic voltammogram of **$Fe(Cpy)_2(dcb)$|ITO** measured in 0.1 M $TBAPF_6$ acetonitrile at a 50 mV/s scan rate.

12. ITO Spectroelectrochemistry

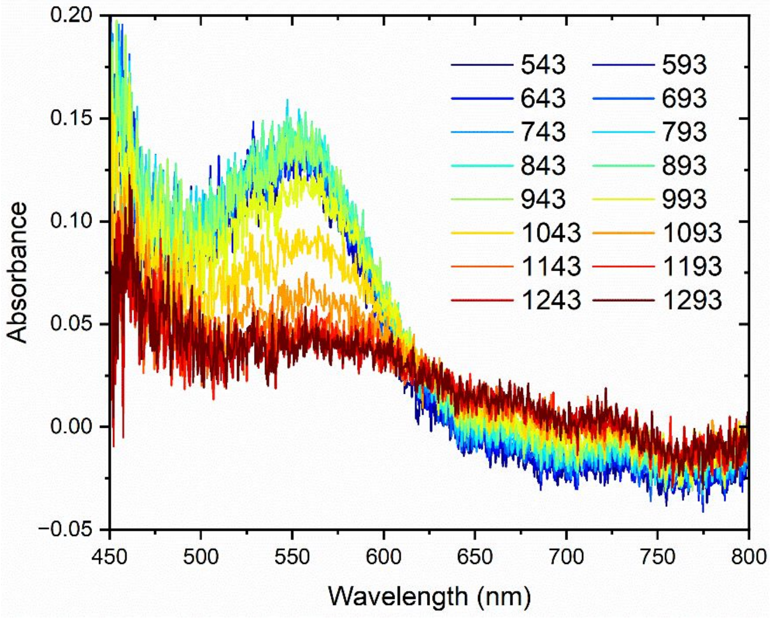

Figure S 29: UV-Vis absorbance of **$Fe(Cpy)_2(dcb)$|ITO** in 0.1 M $TBAPF_6$ acetonitrile at a range of applied potentials (mV, vs NHE).

13. TAS Fluence Dependent Recombination Rate Constants

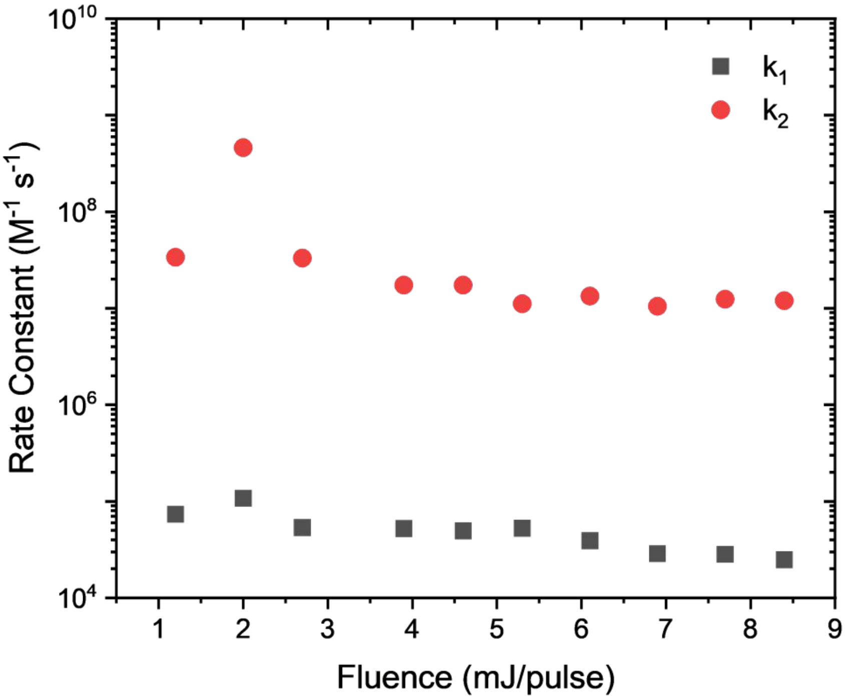

Figure S 30: Recombination rate constants extracted using **equation 1** plotted against the fluence of the laser excitation used.

14. DSSC illustration

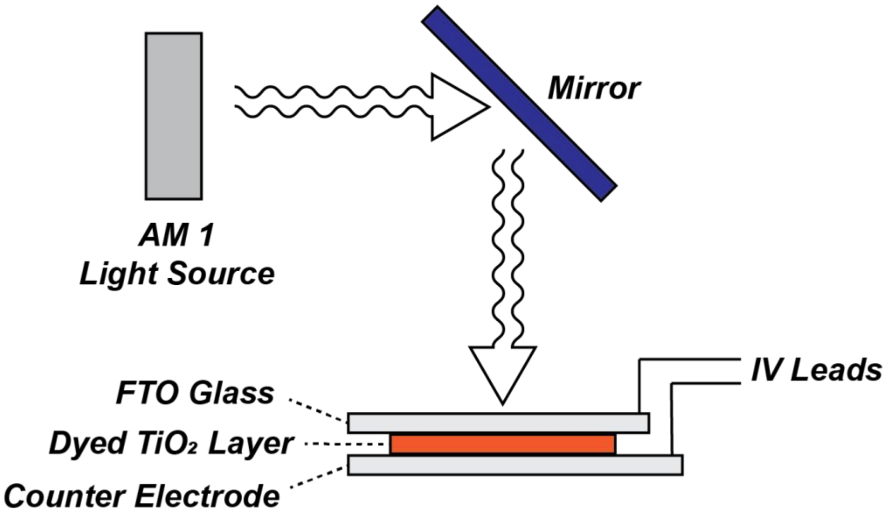

Figure S 31: An illustration detailing the set up used in all operating DSSCs.

14. Photodiode Illustration

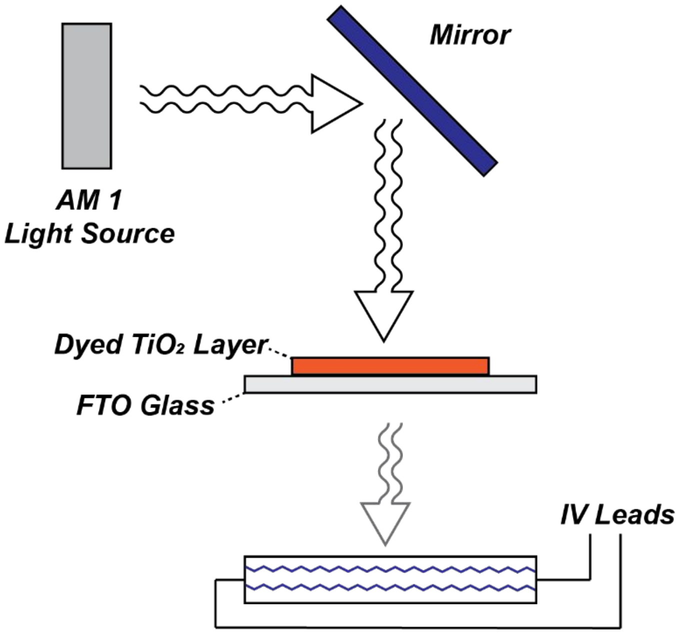

Figure S 32: An illustration detailing how a photodiode was used to determine the fraction of white light absorbed by a given sensitized thin film.

15. References

(1) Sheldrick, G. M. Crystal structure refinement with SHELXL. *Acta Crystallogr C Struct Chem* **2015**, *71* (Pt 1), 3-8. DOI: 10.1107/s2053229614024218 From NLM.
(2) Sheldrick, G. M. A short history of SHELX. *Acta Crystallogr A* **2008**, *64* (Pt 1), 112-122. DOI: 10.1107/s0108767307043930 From NLM.
(3) Macrae, C. F.; Edgington, P.; McCabe, P.; Pidcock, E.; Shields, G. P.; Taylor, R.; Towler, M.; Streek, J. V. d. Mercury: visualization and analysis of crystal structures. *Journal of Applied Crystallography* **2006**, *39*, 453-457.
(4) *Gaussian 16 Rev. C.01*; Wallingford, CT, 2016. (accessed.
(5) Witas, K.; Nair, S. S.; Maisuradze, T.; Zedler, L.; Schmidt, H.; Garcia-Porta, P.; Rein, A. S. J.; Bolter, T.; Rau, S.; Kupfer, S.; et al. Beyond the First Coordination Sphere─Manipulating the Excited-State Landscape in Iron(II) Chromophores with Protons. *Journal of the American Chemical Society* **2024**, *146* (29), 19710-19719. DOI: 10.1021/jacs.4c00552.
(6) Becke, A. D. Density-functional thermochemistry. III. The role of exact exchange. *The Journal of Chemical Physics* **1993**, *98* (7), 5648-5652. DOI: 10.1063/1.464913 (acccessed 11/11/2025).
(7) Lee, C.; Yang, W.; Parr, R. G. Development of the Colle-Salvetti correlation-energy formula into a functional of the electron density. *Physical Review B* **1988**, *37* (2), 785-789. DOI: 10.1103/PhysRevB.37.785.
(8) Tao, J.; Perdew, J. P.; Staroverov, V. N.; Scuseria, G. E. Climbing the Density Functional Ladder: Nonempirical Meta--Generalized Gradient Approximation Designed for Molecules and Solids. *Physical Review Letters* **2003**, *91* (14), 146401. DOI: 10.1103/PhysRevLett.91.146401.
(9) Staroverov, V. N.; Scuseria, G. E.; Tao, J.; Perdew, J. P. Comparative assessment of a new nonempirical density functional: Molecules and hydrogen-bonded complexes. *The Journal of Chemical Physics* **2003**, *119* (23), 12129-12137. DOI: 10.1063/1.1626543 (acccessed 11/11/2025).
(10) Grimme, S.; Ehrlich, S.; Goerigk, L. Effect of the damping function in dispersion corrected density functional theory. *Journal of Computational Chemistry* **2011**, *32* (7), 1456-1465. DOI: https://doi.org/10.1002/jcc.21759.
(11) Marenich, A. V.; Cramer, C. J.; Truhlar, D. G. Universal Solvation Model Based on Solute Electron Density and on a Continuum Model of the Solvent Defined by the Bulk Dielectric Constant and Atomic Surface Tensions. *The Journal of Physical Chemistry B* **2009**, *113* (18), 6378-6396. DOI: 10.1021/jp810292n.
(12) Scholes, G. D.; Curutchet, C.; Mennucci, B.; Cammi, R.; Tomasi, J. How Solvent Controls Electronic Energy Transfer and Light Harvesting. *The Journal of Physical Chemistry B* **2007**, *111* (25), 6978-6982. DOI: 10.1021/jp072540p.
(13) Zobel, J. P.; Kruse, A.; Baig, O.; Lochbrunner, S.; Bokarev, S. I.; Kühn, O.; González, L.; Bokareva, O. S. Can range-separated functionals be optimally tuned to predict spectra and excited state dynamics in photoactive iron complexes? *Chemical Science* **2023**, *14* (6), 1491-1502, 10.1039/D2SC05839A. DOI: 10.1039/D2SC05839A.
(14) Wegeberg, C.; Häussinger, D.; Kupfer, S.; Wenger, O. S. Controlling the Photophysical Properties of a Series of Isostructural d6 Complexes Based on Cr0, MnI, and FeII. *Journal of the American Chemical Society* **2024**, *146* (7), 4605-4619. DOI: 10.1021/jacs.3c11580.
(15) Schmidt, H.; Oglou, R. C.; Tunçer, H. O.; Ghobadi, T. G. U.; Tekir, Ş.; Sertcelik, K. N. O.; Ibrahim, A.; Döhler, L.; Özçubukçu, S.; Kupfer, S.; et al. A Heterodox Approach for Designing Iron Photosensitizers: Pentacyanoferrate(II) Complexes with

Monodentate Bipyridinium/Pyrazinium-Based Acceptor Ligands. *Inorganic Chemistry* **2025**, *64* (14), 7079-7087. DOI: 10.1021/acs.inorgchem.5c00412.
(16) Yaltseva, P.; Maisuradze, T.; Prescimone, A.; Kupfer, S.; Wenger, O. S. Structural Control of Metal-Centered Excited States in Cobalt(III) Complexes via Bite Angle and π–π Interactions. *Journal of the American Chemical Society* **2025**, *147* (32), 29444-29456. DOI: 10.1021/jacs.5c09616.
(17) Neese, F. The ORCA program system. *WIREs Computational Molecular Science* **2012**, *2* (1), 73-78. DOI: https://doi.org/10.1002/wcms.81.
(18) Pantazis, D. A.; Neese, F. All-electron scalar relativistic basis sets for the 6p elements. *Theoretical Chemistry Accounts* **2012**, *131* (11), 1292. DOI: 10.1007/s00214-012-1292-x.
(19) Shillito, G. E.; Hall, T. B. J.; Preston, D.; Traber, P.; Wu, L.; Reynolds, K. E. A.; Horvath, R.; Sun, X. Z.; Lucas, N. T.; Crowley, J. D.; et al. Dramatic Alteration of 3ILCT Lifetimes Using Ancillary Ligands in [Re(L)(CO)3(phen-TPA)]n+ Complexes: An Integrated Spectroscopic and Theoretical Study. *Journal of the American Chemical Society* **2018**, *140* (13), 4534-4542. DOI: 10.1021/jacs.7b12868.
(20) Zedler, L.; Kupfer, S.; Schmidt, H.; Dietzek-Ivanšić, B. Oxidation-state sensitive light-induced dynamics of Ruthenium-4H-Imidazole complexes. *Chemistry – A European Journal* **2024**, *30* (13), e202303079. DOI: https://doi.org/10.1002/chem.202303079.
(21) Yang, G.; Blechschmidt, L.; Zedler, L.; Zens, C.; Witas, K.; Schmidt, M.; Esser, B.; Rau, S.; Shillito, G. E.; Dietzek-Ivanšić, B.; et al. Excited State Branching Processes in a Ru(II)-Based Donor–Acceptor–Donor System. *Chemistry – A European Journal* **2025**, *31* (31), e202404671. DOI: https://doi.org/10.1002/chem.202404671.
(22) Koch, A.; Kinzel, D.; Dröge, F.; Gräfe, S.; Kupfer, S. Photochemistry and Electron Transfer Kinetics in a Photocatalyst Model Assessed by Marcus Theory and Quantum Dynamics. *The Journal of Physical Chemistry C* **2017**, *121* (30), 16066-16078. DOI: 10.1021/acs.jpcc.7b02812.
(23) Shillito, G. E.; Rau, S.; Kupfer, S. Plugging the 3MC Sink in RuII-Based Photocatalysts. *ChemCatChem* **2023**, *15* (4), e202201489. DOI: https://doi.org/10.1002/cctc.202201489.
(24) Yang, G.; Shillito, G. E.; Zens, C.; Dietzek-Ivanšić, B.; Kupfer, S. The three kingdoms—Photoinduced electron transfer cascades controlled by electronic couplings. *The Journal of Chemical Physics* **2023**, *159* (2). DOI: 10.1063/5.0156279 (acccessed 2/24/2026).
(25) Yang, G.; Shillito, G. E.; Seeber, P.; Wenger, O. S.; Kupfer, S. Unraveling the photoredox chemistry of a molecular ruby. *Chemical Science* **2025**, *16* (39), 18113-18125, 10.1039/D5SC05170C. DOI: 10.1039/D5SC05170C.
(26) Staniszewska, M.; Kupfer, S.; Guthmuller, J. Theoretical Investigation of the Electron-Transfer Dynamics and Photodegradation Pathways in a Hydrogen-Evolving Ruthenium–Palladium Photocatalyst. *Chemistry – A European Journal* **2018**, *24* (43), 11166-11176. DOI: https://doi.org/10.1002/chem.201801698.
(27) Staniszewska, M.; Kupfer, S.; Guthmuller, J. Effect of the Catalytic Center on the Electron Transfer Dynamics in Hydrogen-Evolving Ruthenium-Based Photocatalysts Investigated by Theoretical Calculations. *The Journal of Physical Chemistry C* **2019**, *123* (26), 16003-16013. DOI: 10.1021/acs.jpcc.9b03621.
(28) Zens, C.; Friebe, C.; Schubert, U. S.; Richter, M.; Kupfer, S. Tailored Charge Transfer Kinetics in Precursors for Organic Radical Batteries: A Joint Synthetic-Theoretical Approach. *ChemSusChem* **2023**, *16* (2), e202202340. DOI: https://doi.org/10.1002/cssc.202202340.

(29) Wang, J.-Z.; Zhou, J.-P.; Wang, Y.; Miao, N.-x.; Guo, Z.-Q.; Lei, Y.-X. The surface reactivity and structural properties of anatase TiO2 (001), (100), (101) and (105) surface researched with DFT. *Proceedings of the National Academy of Sciences, India Section A: Physical Sciences* **2019**, *89* (1), 193-197. DOI: 10.1007/s40010-017-0466-2.
(30) Hjorth Larsen, A.; Jørgen Mortensen, J.; Blomqvist, J.; Castelli, I. E.; Christensen, R.; Dułak, M.; Friis, J.; Groves, M. N.; Hammer, B.; Hargus, C.; et al. The atomic simulation environment—a Python library for working with atoms. *Journal of Physics: Condensed Matter* **2017**, *29* (27), 273002. DOI: 10.1088/1361-648X/aa680e.
(31) Bannwarth, C.; Caldeweyher, E.; Ehlert, S.; Hansen, A.; Pracht, P.; Seibert, J.; Spicher, S.; Grimme, S. Extended tight-binding quantum chemistry methods. *WIREs Computational Molecular Science* **2021**, *11* (2), e1493. DOI: https://doi.org/10.1002/wcms.1493.
(32) Neese, F. Approximate second-order SCF convergence for spin unrestricted wavefunctions. *Chemical Physics Letters* **2000**, *325* (1), 93-98. DOI: https://doi.org/10.1016/S0009-2614(00)00662-X.
(33) Kupfer, S. Computational details: Hot-carrier injection and millisecond charge separation from a robust heteroleptic iron(II) chromophore immobilized on TiO2. Zenodo, 2025, DOI 10.5281/zenodo.17592435.
(34) Garakyaraghi, S.; Danilov, E. O.; McCusker, C. E.; Castellano, F. N. Transient Absorption Dynamics of Sterically Congested Cu(I) MLCT Excited States. *The Journal of Physical Chemistry A* **2015**, *119* (13), 3181-3193. DOI: 10.1021/acs.jpca.5b00901.
(35) Loague, Q. R.; Heidari, M.; Mann, H. J.; Danilov, E. O.; Castellano, F. N.; Galoppini, E.; Meyer, G. J. Structural Gating Enhances Long-Distance Light-Driven Interfacial Electron Transfer. *ACS Central Science* **2024**, *10* (11), 2132-2144. DOI: 10.1021/acscentsci.4c01106.
(36) Rau, S.; Schäfer, B.; Grüßing, A.; Schebesta, S.; Lamm, K.; Vieth, J.; Görls, H.; Walther, D.; Rudolph, M.; Grummt, U. W.; et al. Efficient synthesis of ruthenium complexes of the type (R-bpy)2RuCl2 and [(R-bpy)2Ru(L–L)]Cl2 by microwave-activated reactions (R: H, Me, tert-But) (L–L: substituted bibenzimidazoles, bipyrimidine, and phenanthroline). *Inorganica Chimica Acta* **2004**, *357* (15), 4496-4503. DOI: https://doi.org/10.1016/j.ica.2004.07.007.
(37) Schwalbe, M.; Schäfer, B.; Görls, H.; Rau, S.; Tschierlei, S.; Schmitt, M.; Popp, J.; Vaughan, G.; Henry, W.; Vos, J. G. Synthesis and Characterisation of Poly(bipyridine)ruthenium Complexes as Building Blocks for Heterosupramolecular Arrays. *European Journal of Inorganic Chemistry* **2008**, *2008* (21), 3310-3319. DOI: https://doi.org/10.1002/ejic.200701303.
(38) Beauvilliers, E. E.; Meyer, G. J. Evidence for Cation-Controlled Excited-State Localization in a Ruthenium Polypyridyl Compound. *Inorganic Chemistry* **2016**, *55* (15), 7517-7526. DOI: 10.1021/acs.inorgchem.6b00876.
(39) Heimer, T. A.; D'Arcangelis, S. T.; Farzad, F.; Stipkala, J. M.; Meyer, G. J. An Acetylacetonate-Based Semiconductor−Sensitizer Linkage. *Inorganic Chemistry* **1996**, *35* (18), 5319-5324. DOI: 10.1021/ic960419j.
(40) Chappel, S.; Zaban, A. Nanoporous SnO2 electrodes for dye-sensitized solar cells: improved cell performance by the synthesis of 18nm SnO2 colloids. *Solar Energy Materials and Solar Cells* **2002**, *71* (2), 141-152. DOI: https://doi.org/10.1016/S0927-0248(01)00050-2.
(41) Heidari, M.; Loague, Q.; Bangle, R. E.; Galoppini, E.; Meyer, G. J. Reorganization Energies for Interfacial Electron Transfer across Phenylene

Ethynylene Rigid-Rod Bridges. *ACS Applied Materials & Interfaces* **2022**, *14* (30), 35205-35214. DOI: 10.1021/acsami.2c07151.
